# Genome-wide methylation profiling identified novel differentially hypermethylated biomarker *MPPED2* in colorectal cancer

**DOI:** 10.1186/s13148-019-0628-y

**Published:** 2019-03-07

**Authors:** Simeng Gu, Shujuan Lin, Ding Ye, Sangni Qian, Danjie Jiang, Xiaocong Zhang, Qilong Li, Jinhua Yang, Xiaojiang Ying, Zhenjun Li, Mengling Tang, Jianbing Wang, Mingjuan Jin, Kun Chen

**Affiliations:** 1 Department of Epidemiology and Biostatistics, Zhejiang University School of Public Health, 866 Yuhangtang Road, Hangzhou, 310058 China; 2 Department of Epidemiology and Biostatistics, Zhejiang Chinese Medical University School of Public Health, 548 Binwen Road, Hangzhou, 310053 China; 3 Jiashan Institute of Cancer Prevention and Treatment, 345 Jiefangdong Road, Jiashan, 314100 China; 4 Department of Anorectal Surgery, Shaoxing People’s Hospital, 568 Zhongxingbei Road, Shaoxing, 312000 China; 5 Cancer Institute, the Second Affiliated Hospital, Zhejiang University School of Medicine, 88 Jiefang Road, Hangzhou, 310009 China

**Keywords:** Epigenetics, DNA methylation, Colorectal cancer, EPIC, MPPED2

## Abstract

**Background:**

Epigenetic alternation is a common contributing factor to neoplastic transformation. Although previous studies have reported a cluster of aberrant promoter methylation changes associated with silencing of tumor suppressor genes, little is known concerning their sequential DNA methylation changes during the carcinogenetic process. The aim of the present study was to address a genome-wide search for identifying potentially important methylated changes and investigate the onset and pattern of methylation changes during the progression of colorectal neoplasia.

**Methods:**

A three-phase design was employed in this study. In the screening phase, DNA methylation profile of 12 pairs of colorectal cancer (CRC) and adjacent normal tissues was analyzed by using the Illumina MethylationEPIC BeadChip. Significant CpG sites were selected based on a cross-validation analysis from The Cancer Genome Atlas (TCGA) database. Methylation levels of candidate CpGs were assessed using pyrosequencing in the training dataset (tumor lesions and adjacent normal tissues from 46 CRCs) and the validation dataset (tumor lesions and paired normal tissues from 13 hyperplastic polyps, 129 adenomas, and 256 CRCs). A linear mixed-effects model was used to examine the incremental changes of DNA methylation during the progression of colorectal neoplasia.

**Results:**

The comparisons between normal and tumor samples in the screening phase revealed an extensive CRC-specific methylomic pattern with 174,006 (21%) methylated CpG sites, of which 22,232 (13%) were hyermethylated and 151,774 (87%) were hypomethylated. Hypermethylation mostly occurred in CpG islands with an overlap of gene promoters, while hypomethylation tended to be mapped far away from functional regions. Further cross validation analysis from TCGA dataset confirmed 265 hypermethylated promoters coupling with downregulated gene expression. Among which, hypermethylated changes in *MEEPD2* promoter was successfully replicated in both training and validation phase. Significant hypermethylation appeared since precursor lesions with an extensive modification in CRCs. The linear mixed-effects modeling analysis found that a cumulative pattern of *MPPED2* methylation changes from normal mucosa to hyperplastic polyp to adenoma, and to carcinoma (*P* < 0.001).

**Conclusions:**

Our findings indicate that epigenetic alterations of *MPPED2* promoter region appear sequentially during the colorectal neoplastic progression. It might be able to serve as a promising biomarker for early diagnosis and stage surveillance of colorectal tumorigenesis.

**Electronic supplementary material:**

The online version of this article (10.1186/s13148-019-0628-y) contains supplementary material, which is available to authorized users.

## Introduction

Colorectal cancer (CRC) is the third most common diagnosed cancer and the fourth leading cause of cancer death worldwide [1]. It results from a series of changes at both genetic and epigenetic levels. Despite a large number of genetic alterations have so far been described, little has found their way in clinical practice. An in-depth understanding of epigenetic regulatory network might do work in screening, diagnosis, and treatment of CRC.

At epigenetic level, DNA methylation changes is one of the hallmark events in carcinogenesis, characterized by global hypomethylation and paradoxical gene-specific hypermethylation [2, 3]. Hypomethylation is primarily involved in chromosomal instability and global loss of imprinting, while gene-specific hypermethylation anchors to promoters, it causes transcriptional silencing of tumor suppressor genes and consequently sets the stage for neoplastic transformation. Deregulated DNA methylation is well known to be associated with CRC [4, 5]. The first evidence of DNA methylation contributing to CRC was presented by Goelz et al. [6], in which a global loss in DNA methylation was identified. Then, extensive efforts have been made in identifying aberrant hypermethylation in promoters of CRC-related suppressor genes, such as *APC*, *SFRP2*, *SEPT*, and *CDH1* [7–9]. Remarkably, a near universal phenomenon that gene-specific hypermethylation occurred in both pre-neoplastic and neoplastic phase of colorectal cancer was observed [10, 11]. This might shed light on the process of tumorigenesis. However, few studies have defined precisely the hierarchy of methylation events during the transformation from normal epithelial cells to malignant cells in colorectal carcinogenesis.

It is becoming increasingly apparent that the occurrence of molecular alterations could be found not only in tumor tissue but also in histological normal-appearing tissue adjacent to the tumor [12–14]. The presence of such molecular alterations in histological normal-appearing tissue is commonly known as field cancerization or field effect [15]. This has been thought to constitute the earliest clone in the carcinogenesis process. In colorectal carcinogenesis, a few number of gene-specific hypermethylation events have been reported in normal-appearing colonic mucosa from CRC patients (such as *APC*, *DKKI*, *MGMT*, *CDKN2A*, and *SFRP4*) [16–18]. In addition, previous studies have also demonstrated the effect of aberrant DNA methylation on suppressor genes such as *MINT1*, *MINT31*, *SLC5A8*, and *MGMT*, during adenoma-carcinoma sequence [19–21]. These findings suggest that the presence of field effect in methylation might be a useful intermediate biomarker in etiologic studies. A better understanding of when these epigenetic tags occur and how they take part in colorectal progression may represent a practical opportunity for colorectal cancer risk assessment.

Therefore, the aim of the present study was to investigate the altered DNA methylome for identification of methylation biomarkers in CRC. Additionally, we hypothesized that field effect due to DNA methylation might appear since the normal-appearing colonic mucosa adjacent to tumor tissue. A quantitative model was introduced to give a full scope of changes in epigenetic pattern during the carcinogenic process.

## Methods

### Study design and study population

**Fig. 1:**
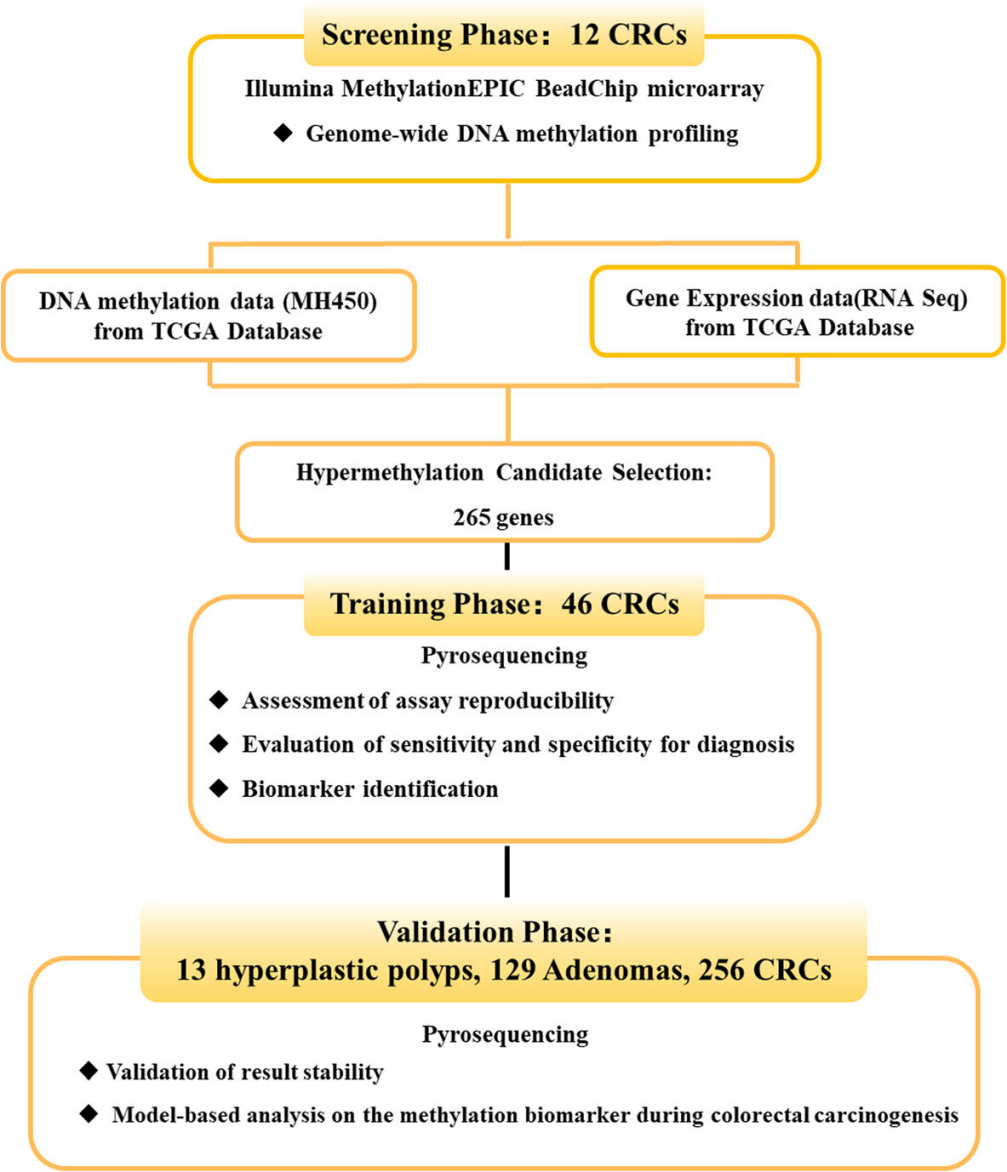
Study flowchart illustrating the design of the study

This study has been processed through three phases. In the screening phase, a genome-wide methylation scan on cancerous and paired normal tissues from 12 CRC patients was performed, followed by a cross-validation analysis including transcriptome and DNA methylome data from the publicly available database. Then, in the training phase, 46 pairs of CRC tissue samples were tested for a given list of candidates to assess the reproducibility of Illumina MethylationEPIC BeadChip (EPIC) platform. Thirteen hyperplastic polyps, 129 adenomas, and 256 CRCs were collected into the validation phase, in which only potential genes with a high discriminative performance in the training phase were further testified. In order to evaluate the onset of colorectal neoplastic progression, we defined and evaluated methylation-changing patterns in the transition from normal mucosa related to low risk and high risk of CRC, to hyperplastic polyp, and to adenoma and carcinoma in the validation phase. The definitions for low- and high-risk normal mucosa were as follows: normal colon mucosa from individuals who had no history of CRC was considered as low-risk normal mucosa; normal colon mucosa from those who have primary solid CRCs, which might be at an increased risk of metachronous CRCs, was considered as high-risk normal mucosa. A flowchart is shown in Fig. 1.

The enrollment of CRC cases was based on Shaoxing People’s Hospital between 2015 and 2017. Participants with colorectal polyps (hyperplastic polyp or adenoma) were drawn from a population-based cohort since 1989 in Jiashan County, Zhejiang Province, China. It has been described in detail previously [22, 23]. For each participant, a histologically confirmed colorectal lesion (hyperplastic polyp, adenoma, or carcinoma) and a distant normal mucosa sample were obtained. Individuals were excluded according to the following criteria: (1) familial adenomatous polyposis (FAP), (2) a history of CRC, and (3) preoperative anticancer treatment. Tumor stage was determined according to the American Joint Committee on Cancer (AJCC) TNM staging system of CRC. A complete list of participants included in each phase is shown in Table 1.

**Table 1:** Characteristics of the study population

Characteristics	Screening phase	Training phase	Validation phase		
	CRCs	CRCs	Hyperplastic polyps	Adenomas	CRCs
	(*n* = 12)	(*n* = 46)	(*n* = 13)	*(n* = 129)	(*n* = 256)
Age, mean ± SD	63.50 ± 4.15	64.11 ± 9.03	59.62 ± 5.99	62.14 ± 7.21	63.51 ± 9.55
Gender					
Male	7	23	6	79	159
Female	5	23	7	50	97
Location					
Colon	6	21	8	110	114
Rectal	6	25	5	19	142
Stage					
I	3	11			28
II	3	12			123
III	3	12			95
IV	3	11			10

*SD* standard deviation

Every participant signed an informed consent prior to study participation. The study was approved by the Medical Ethics Committee of Zhejiang University School of Medicine.

### DNA extraction and bisulfite conversion

DNA from fresh-frozen samples was extracted using DNA tissue Kit (Omega Bio-Tek, Norcross, GA, USA). DNA was used for bisulfite conversion, which was performed using EZ DNA Methylation-Gold Kit (Zymo Research, Irvine, CA, USA). All procedures were conducted according to the manufacturer’s instructions.

### Genome-wide DNA methylation analysis

Genome-wide DNA methylation analysis was performed using a recently developed EPIC BeadChip (Illumina, San Diego, CA, USA), which covers more than 850,000 CpG sites for methylation study. Raw fluorescence intensities were loaded in BeadStudio software to field *β* values, which represent the methylation score of each CpG site. They range from 0 (non-methylated) to 1 (fully methylated). Prior to identification of differentially methylated probes, preprocessing steps, including data filtering, correction, and normalization, were implemented. The probe call rate at least 95% coverage per sample and probes detection *P* value < 0.01 were required, otherwise they should be excluded. In addition, probes on X and Y chromosomes were also removed. Background correction, dye-bias, and beta-mixture quantile normalization (BMIQ) procedures were performed using Bioconductor packages [24, 25]. All 12 paired CRC samples included into the screening phase passed the quality control from EPIC platform with a probe call rate > 99%, and 844,711 probes out of 853,307 were included in the following analysis.

Comparisons between cancerous and paired normal tissues were performed using the Illumina Methylation Analyzer (IMA) package. Differentially methylated CpG sites were identified when *β* difference > 0.2 and adjusted *P* value (Benjamini-Hochberg method, FDR) < 0.05.

### External validation of the candidate biomarker genes

We downloaded data from the Cancer Genome Atlas (TCGA) project (https://cancergenome.nih.gov) to validate our results. DNA methylation data consisting of 351 samples (38 normal, 313 tumor) was generated using Illumina HumanMethylation450 (HM450) BeadChip. The same processing procedures and filtering criteria as described above were applied to identify methylation differences between cancerous and normal tissues. Meanwhile, gene expression data, including 51 normal and 641 tumor tissue samples, was obtained from Illumina RNA sequencing (RNA-Seq) platform. After averaging the within-array replicate spots and removing genes that are zero in all libraries, preprocessing data was normalized using trimmed mean of Mvalues (TMM) method. We tested the differentially expressed genes between cancerous and normal tissues with a literature-based filtering criterion (FDR < 0.05 and fold change > 2) [26–28]. Two packages named edgeR and limma were used for data preprocessing, normalization, and differential expression analysis of TCGA samples.

### Validation of methylation status by pyrosequencing

DNA methylation level of particular CpG sites located in the promoter region of candidate genes was quantified by pyrosequencing. Median of individual CpG values represented the DNA methylation status of each gene. A primer set is shown in Additional file 1: Table S1. Pyrosequencing reactions and quantification of DNA methylation were ran on the Pyromark Q96 MD pyrosequencing system (QIAGEN, Valencia, CA, USA).

In order to ensure accuracy and authenticity of our pyrosequencing results, quality control measures were implemented as follows: (1) 1% agarose gel Electrophoresis was chose to test the quality of the extracted DNA. (2) A sample set that included serial dilutions of fully methylated and non-methylated DNAs (Human Methylated & Non-methylated DNA Set, Zymo Research, Freiburg im Breisgau, Germany) (0%, 25%, 50%, 75%, and 100%) was used to control DNA standards. (3) No template controls were included in each experimental run. In addition, all samples were mixed across plates.

### Statistical analysis

Data were summarized as mean, standard deviation (SD), median, 5th and 95th percentiles for continuous variables, and absolute and relative frequencies for categorical variables. Differences in the distribution of genome methylation pattern were tested by Pearson *χ*^2^ test.

Differences in methylation and gene expression between cancerous and normal tissues were tested using paired Student’s *t* test and empirical analysis based on the negative binomial distributions [29], respectively. A three-step approach was used to identify biomarkers associated with colorectal carcinogenesis. In the screening phase, we reanalyzed these differentially methylated probes with a set of strict filtering criteria, including (1) *β* difference > 0.35 and FDR < 0.05, (2) hypermethylated CpG sites were annotated in both traditional promoter regions (TSS1500, TSS200, 5′UTR and first exon) and CpG islands (CGIs). After the cross-validation analysis using DNA methylation microarray and RNA-Seq data from TCGA database, five top genes showing consistently hypermethylated changes in promoters were selected into the training phase. To verify the accuracy and specificity of these five candidates as a signature, the discriminative performance of selected candidates was assessed by receiver operating characteristic (ROC) curves, and the area under the ROC curve (AUC), sensitivity, and specificity at the optimal cut-offs were calculated. Only potential genes with a high discriminative performance (sensitivity > 0.80; specificity > 0.90) [30] were further selected into the validation phase. Based on the conception of field effect, linear mixed-effects modeling analysis was used to describe the methylation changes in the transition from colorectal normal mucosa from individuals without a history of cancer (*n* = 142) to normal mucosa surrounding CRC tissue (*n* = 256), to hyperplastic polyp (*n* = 13), to adenoma (*n* = 129), and to carcinoma (*n* = 256). Correlated errors resulting from within-individual comparisons were assessed by setting up with a random-effect variable for non-independent measurements from matched lesion and normal samples. All these tests were two-sided. *P* value < 0.05 was considered statistically significant. Statistical analyses were performed using R software (Version 3.3.2).

## Results

### Analysis of global methylation profiles in colorectal cancer

**Fig. 2:**
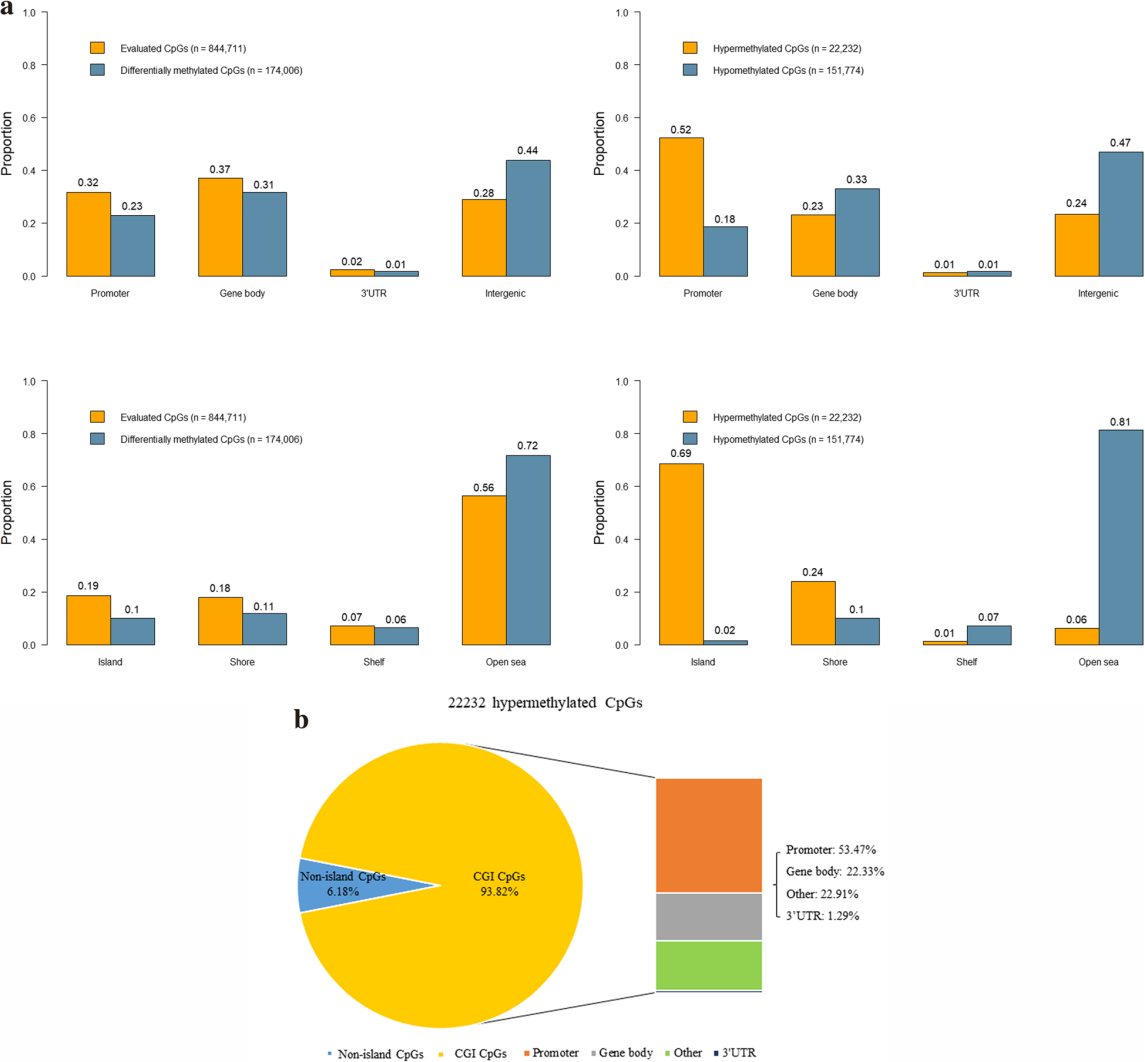
Differentially methylated probes between CRC tissues and adjacent normal tissues in the screening phase. **a** Classification of the differentially methylated probes according to gene content and CpG islands. **b** A zoom-in view of CGI-specific hypermethylation

In the screening phase, analysis of the differential methylation between 12 pairs of tumor tissues and adjacent normal tissues from CRC patients identified a total of 174,006 (21%) CpG sites to be differentially methylated. They were mainly detected in the region of low CpGs (124,917/174,006 CpG sites 72% vs. 477,028/844,711 CpG sites, 56%, *P* < 0.0001), commonly named “open sea,” and far from gene promoters (134,228/174,006 CpG sites, 77% vs. 576,610/844,711 CpG sites, 68%, *P* < 0.0001), in comparison with the reference distribution of probes on EPIC platform (Fig. 2a left panel).

Among the identified differentially methylated CpG sites, 22,232 (13%) were significantly hypermethylated and 151,774 (87%) were significantly hypomethylated. An overview of the frequency of all hyper- and hypomethylated CpG sites classified by CGI and gene content is shown in Fig. 2a (right panel). Significant hypomethylation was predominantly observed in open sea (123,542/151,774 CpG sites, 81%) and intergenic regions (71,100/151,774 CpG sites, 47%), while most significant hypermethylation was specifically located around CGIs (20,857/22,232 CpG sites, 94%) and gene promoters (11,636/22,232 CpG sites, 52%). Then, the overlap of CGI hypermethylation and genomic locations was examined in detail. Approximately, 53% (11,151 CpG sites) of CGI hypermethylation overlapped specifically with gene promoters (Fig. 2b).

### Cross-validation analysis with TCGA data

Considering that promoter-associated hypermethylation can trigger transcriptional silencing of the target gene, we merged the promoter methylation with RNA-Seq gene expression data by using data from TCGA and further identified a list of 265 genes undergoing promoter hypermethylation during colorectal tumor progression. As shown in Additional file 2: Table S2, this collection of genes showed an overlap between hypermethylation and downregulated expression in TCGA database.

**Fig. 3:**
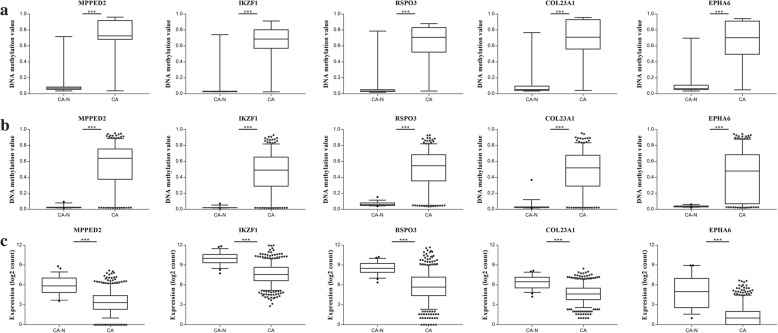
Methylation and expression levels of five selected genes in the screening phase. **a** Methylation levels of *MPPED2*, *IKZF1*, *RSPO3*, *COL23A1*, and *EPHA6* in CRC tissues and paired normal tissues using EPIC array. **b** Methylation levels of *MPPED2*, *IKZF1*, *RSPO3*, *COL23A1*, and *EPHA6* in CRC tissues and adjacent normal tissues using TCGA HM450 data. **c** mRNA expression of *MPPED2*, *IKZF1*, *RSPO3*, *COL23A1*, and *EPHA6* in CRC tissues and adjacent normal tissues using TCGA RNA-Seq data. ***P < 0.001. CA-N, CRC surrounding normal tissue; CA, primary CRC

**Fig. 4:**
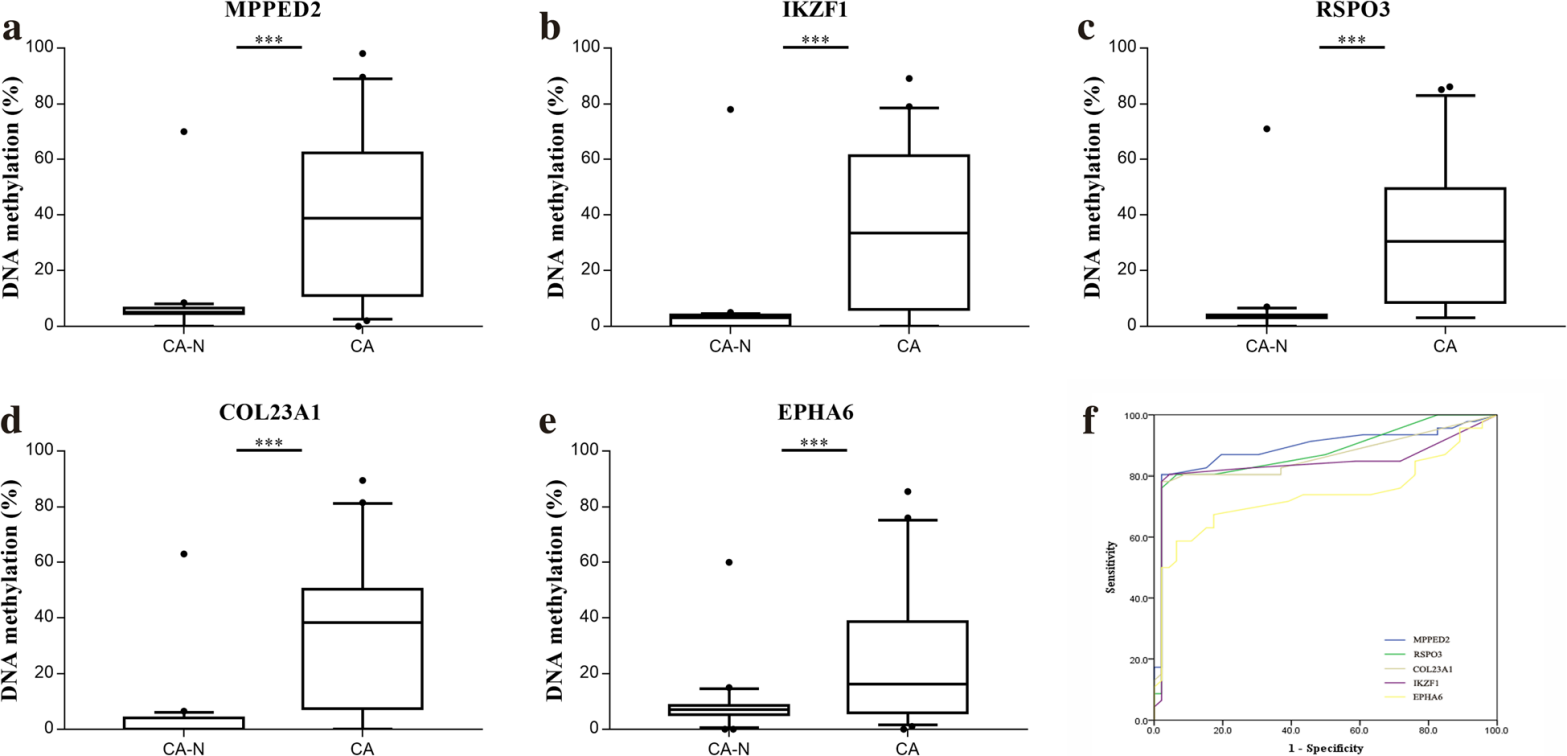
Pyrosequencing analyses of five selected candidates in training phase. Pyrosequencing results are shown for methylation levels of **a** *MPPED2*, **b** *IKZF1*, **c** *RSPO3*, **d** *COL23A1*, and **e** *EPHA6*. **f** The discriminative ability of the five selected genes between CRC tissues and adjacent normal tissues by ROC analysis. ***P < 0.001. CA-N, CRC surrounding normal tissue; CA, primary CRC

Among the list of CpG sites, we focused on five sites mapping the promoter regions of their own genes for following training analysis (Fig. 3). Three of them were novel hypermethylated genes (cg11855526 in *MPPED2*, cg25437410 in *COL23A1*, and cg15093079 in *EPHA6*) ranking on the top, which were considered as candidate biomarkers. The remaining two (cg20078466 in *IKZF1* and cg07279933 in *RSPO3*), were chosen based on the publications, which were considered as the reference. Figure 4 presents the position of candidate gene promoters in relation to the respective CGIs.

### Validation of promoter methylation status using pyrosequencing

**Fig. 5:**
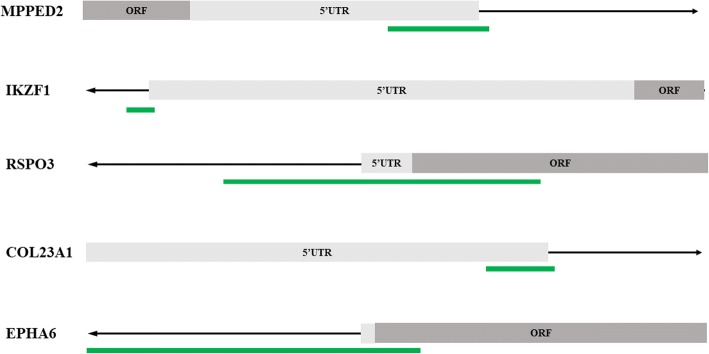
Schematic representation of the relative position between selected genes and corresponding CpG islands. The orientation of each gene is indicated by the arrow. CpG islands are shown as green bars. 5′UTR or open reading frame (ORF) are shown as gray boxes

In the training phase, DNA methylation status of the above five candidate genes in 46 CRCs and paired normal tissues was detected by pyrosequencing. Box plots showing the distribution of *β* values of these genes’ methylation status are shown in Fig. 5a–e. All five genes demonstrated significantly differential hypermethylation. The increase in methylation status between cancerous and paired normal mucosa was 33.35%, 30.54%, 27.28%, 31.62%, and 18.18% for *MPPED2*, *IKZF1*, *RSPO3*, *COL23A1*, and *EPHA6*, respectively (all *P* < 0.001). Further Pearson correlation analysis showed a strong correlation between the methylation level of *MPPED2* and that of *RSPO3* (*R* = 0.81, *P* < 0.001) (Additional file 3: Figure S1b).

ROC curve analyses revealed that methylation status of each individual genes significantly distinguished primary carcinoma from normal mucosa, as measured by sensitivity, specificity, and AUC value (*MPPED2*: sensitivity 0.804, specificity 0.978, AUC 0.890; *IKZF1*: sensitivity 0.761, specificity 0.978, AUC 0.875; *RSPO3*: sensitivity 0.783, specificity 0.978, AUC 0.858; *COL23A1*: sensitivity 0.783, specificity 0.978, AUC 0.840; *EPHA6*: sensitivity 0.587, specificity 0.935, AUC 0.736) (Fig. 5f, Additional file 4: Table S3). Among them, *MPPED2* presented a high discriminative performance. Therefore, it was chosen for further validation study.

### Analysis of aberrant methylation pattern of *MPPED2* during neoplastic progression of colorectal cancer

**Fig. 6:**
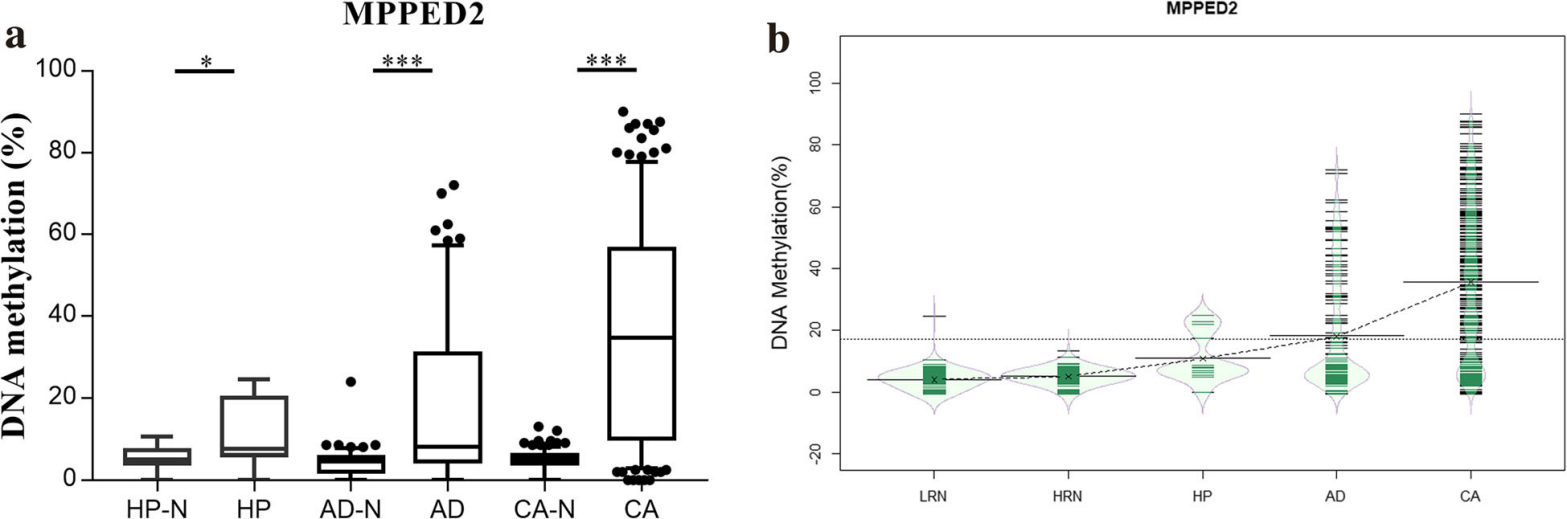
DNA methylation level of *MPPED2* examined by pyrosequencing in validation phase. **a** Methylation level of *MPPED2* in colorectal lesions (hyperplastic polyps, adenoma, and carcinoma) and paired normal tissues. All the three groups showed significant difference (*P* < 0.05). **b** Bean plots representing methylation levels of *MPPED2* in low-risk normal mucosa, high-risk normal mucosa, hyperplastic polyp, adenoma, and CRC. The distributions of individual observations are shown as small horizontal lines in each scatter plot together with density distribution (diamond-shaped profile), mean value of each group subset (solid horizontal line), and overall mean value (solid horizontal line across all subsets). *P < 0.05; ***P < 0.001. HP-N, hyperplastic polyp surrounding normal tissue; HP, hyperplastic polyp; AD-N, adenoma surrounding normal tissue; AD, adenoma; CA-N, CRC surrounding normal tissue; CA, primary CRC. LRN, low-risk normal tissue; HRN, high-risk normal tissue

In order to define the *MPPED2* methylation changes during colorectal neoplastic progression, DNA methylation status among 796 colorectal tissue samples, including colorectal lesions and paired normal tissues from 13 hyperplastic polyps, 129 adenomas, and 256 primary carcinomas, was assessed during the validation phase. As shown in Fig. 6a, the methylation status of *MPPED2* increased significantly in each colorectal lesion groups (all *P* < 0.05), in comparison with their respective normal groups. The methylation changes also increased quantitatively with the neoplastic progression (5.58% for hyperplastic polyps; 14.5% for adenomas; 30.56% for carcinomas). To examine the hierarchy of methylation events during colorectal neoplastic progression, the linear mixed-effects modelling analysis was performed. We found that *MPPED2* methylation pattern showed a stepwise increase from low-risk normal mucosa to high-risk normal mucosa, to hyperplastic polyp, to adenoma, and to carcinoma (*P* < 0.001). A direct overview of this tendency was noted by intensity coefficients (4.07 for low-risk normal mucosa; 5.02 for high-risk normal mucosa; 10.86 for hyperplastic polyp; 17.95 for adenoma; 35.58 for carcinoma, respectively) (Fig. 6b). We also examined the interaction between methylation status and clinical characteristics. However, these results did not present any statistical significance (Additional file 3: Figure S1).

## Discussion

In this study, we performed a comprehensive epigenomic profiling assessment in CRC patients for screening promising biomarkers and carried out a model-based analysis to determine the hierarchy of DNA methylation changes during the carcinogenic process. Our results showed global alterations in DNA methylation patterns in CRC tissues with a large number of differentially methylated CpG sites, compared to their adjacent normal tissues. We also found that the pattern of differential methylation showed opposite characteristics between hypomethylation and hypermethylation across gene and island distribution. Analyses on both TCGA data and internal datasets provided the confirmatory evidence for our findings. Linear mixed-effects modeling analysis revealed that DNA methylation levels of *MPPED2* increased sequentially from normal to hyperplastic polyp, to adenoma, and to carcinoma.

As observed in many cancers [31–33], we found a common methylated pattern with 174,006 (21%) differentially methylated CpG sites while comparing CRC tissues to matched normal tissues, suggesting the widespread aberrations in methylation status during cancer development. Remarkably, we found that a majority of the identified CpG sites were hypomethylation rather than hypermethylation, which were different from what have been shown in general array-based studies [34–37]. This could be explained by the increased genome coverage of the EPIC array. To be specific, the Illumina 27 K BeadChip (27 K) array targeted about 27,000 CpG sites and interrogated at least one site per gene with a bias towards CGIs. The second-generation product, HM450 array, provided more than 485,000 probes focusing on 99% of RefSeq genes, while a large number of new probes in EPIC array solve the limited interrogation in non-CGI regions before. Therefore, our findings with EPIC platform might provide a more unbiased and reasonable map of CRC epigenome.

CGI hypermethylation in promoter of specific genes has been linked to epigenetic transcriptional silencing, which appears to be crucial in the early stage of carcinogenesis. It is worthwhile to understand cancer-specific promoter hypermethylation with transcriptional repression in CRC. Therefore, we extended this analysis by performing cross-validation analysis using DNA methylation microarray and RNA-Seq data from TCGA database. Within 649 genes, 1666 CGI promoter CpG sites were further confirmed to be hypermethylated. However, not all the expression of these genes were significantly downregulated in CRCs compared with normal mucosa. The result indicates the fact that many epigenetic alterations in cancers are passengers that may occur as a consequence of or in association with carcinogenesis [38]. Moreover, multiple regulation mechanisms may implicate in gene expression, which could obscure a direct view of correlations between DNA methylation and gene expression. Although much remains unknown concerning the involvement of multiple regulation mechanisms in human cancers, it is feasible to pinpoint crucial genes that are suspectful for methylation-associated gene inactivation.

Among the cluster of candidate genes characterized by epigenetic silencing, five genes, named *MPPED2*, *COL23A1*, *EPHA6, RSPO3*, and *IKZF1*, were identified as of potential interest for CRC. They all have been reported to be associated with human cancers, which support our findings a possible role in the pathogenesis of CRC.

*MPPED2* gene encodes a new metallophosphoesterase protein in mammals, and it normally regulates many essential cellular functions including differentiation, proliferation, and apoptosis [39, 40]. It is expressed ubiquitously in most human tissues, and the downregulated expression of *MPPED2* has been reported in several malignant tumors, including oral squamous cell carcinoma [40], papillary thyroid carcinoma [41], and cervical cancer [42]. Notably, recent evidence indicates that the tumor-suppressing activity of *MPPED2* could be epigenetically modified by DNA methylation [43]. Liguori et al. found that *MPPED2* expression significantly increased after the treatment with demethylation agent. In vivo, *MPPED2* could serve a function of anti-proliferation, which showed that the biochemical activity of *MPPED2* was beneficial for cancer suppression [39]. The above findings support the hypothesis that hypermethylation-induced down-expression is the most likely mechanism of *MPPED2* silencing during carcinogenesis.

Investigation of the other genes of possible biological significance in CRC identified that *EPHA6* was described as the tumor suppressor gene according to its role in the regulation of angiogenesis process. It has been found to be downregulated in colorectal cancer [44, 45]. *COL23A1* is known to be one of the transmembrane collagens. Survival analysis showed significant prognostic implications of *COL23A1* expression in non-small cell lung cancer [46] and prostate cancer [47]. As for *RSPO3*, our result is consistent with previous findings that *RSPO3* could be transcriptionally downregulated by promoter hypermethylation in CRC [35]. *IKZF1* was described as a tumor suppressor gene based on its role in regulation of cellular proliferation [48, 49]. It has been shown to be hypermethylated in CRC [50] and acute lymphoblastic leukemia [51]. Though there existed a strong correlation of methylation levels between *MPPED2* and *RSPO3*, no definite mechanism could explain this phenomenon. One recent computational study [52] showed that both *MPPED2* and *RSPO3* play a role in carcinogenetic process across cancer types, which suggests that they might be part of the active processes underlying colorectal carcinogenesis.

It should be mentioned that some of the current hypotheses highlight that the regulatory role of epigenetic events could be implicated in histologically normal tissues surrounding cancer lesions, which is known as the field cancerization effect [53, 54]. DNA methylation has been shown to be involved in field effect in a variety of tissues, including esophageal mucosa in Barrett’s esophagus [55], normal-appearing gastric mucosa [56], and colonic mucosa effected by ulcerative colitis [57], highlighting the possible role of epigenetic alterations in cancer development. To answer the question when methylation levels of candidates occur significant changes during colorectal cancer progression, a linear mixed-effects model was used to combine the type of tissue, within- and between-individual comparisons, and methylation changes of candidate biomarkers [58]. The model allowed us to quantify the methylation changes in the transition from normal colonic mucosa to hyperplastic polyp, to adenoma, and to carcinoma, while taking into account within-individual comparisons. In this study, cumulative methylation alternations of *MPPED2* promoter showed a significant association with the progression of colorectal neoplasia, suggesting that aberrant methylation is an early event and occurs sequentially during colorectal neoplastic progression. It might be a useful biomarker for early detection and risk assessment in colorectal cancer.

Array-based analysis is a useful tool for genome-wide DNA methylation screening. The new-generation microarray, EPIC platform provides substantially increased genomic coverage than prior studies, therefore allowing identification of novel methylated CpG sites that have not been previously revealed. Although reports using EPIC platform are still scarce, evaluation analysis performed by Pidsley et al. [59], Solomon et al. [60], and Kling et al. [61] found that data from HM450, EPIC, and whole-genome bisulphite sequencing (WGBS) were highly reproducible across technical and biological replicates. However, one recent study [62] demonstrated that data from HM450 platform showed general congruence with data obtained from the same probes using the EPIC platform. Nevertheless, the combined application of data from EPIC and existed HM450 platform are encouraged for identifying new important insights in genomic regulation in disease states.

There are several limitations in this study. First, matched expression analysis was unavailable, which did not allow us to give a directly causal inference between DNA methylation and gene expression. Nevertheless, integration from the published database pinpointed the potential negative associations between DNA methylation and gene expression in CRC tissues. Second, for hyperplastic polyps, the sample size in validation phase was modest, resulting in a less widespread density shape in linear mixed-effects model analysis. Last, although we found that aberrant methylation of *MPPED2* could serve as an amplifiable signal in colorectal tumorigenesis, external validations consisting of one or more dataset from different institutions are still needed to evaluate the performance and potential clinical value of *MPPED2* in CRC. Furthermore, it might be instructive to use different sample types, such as stool or plasma as well. Taken together, although we successfully assessed epigenetic alterations of *MPPED2* promoter hypermethylation during the colorectal neoplastic progression, further studies with the diversity of study population and sample types, and larger sample size are warranted to validate the above findings and elucidate the corresponding mechanism in colorectal carcinogenesis.

## Conclusion

In summary, our work gave a detailed assessment of DNA methylation pattern with over 850,000 CpG probes for CRCs and revealed epigenetically regulated candidate genes in colorectal carcinogenesis. Specifically, our results provide the first evidence that tumor-specific hypermethylation in *MPPED2* promoter occurs frequently in CRCs and its increasing accumulation means the further development of colorectal neoplasia. The findings may offer an instructive clue for understanding the role of DNA methylation in the development of early colorectal neoplasia and provide promising signature for the use of epigenetic profiling in CRC detection and therapy decisions.

## Additional files

Additional file 1: **Table S1.** Pyrosequencing methylation assay Primers. (DOCX 13 kb)

Additional file 2: **Table S2.** A list of differentially hypermethylated and expressed genes identified by both EPIC array and TCGA database. (DOCX 173 kb)

Additional file 3: **Figure S1.** a Pearson correlation analysis between age and methylation level of *MPPED2*. b Pearson correlation analysis between methylation levels of five selected genes **c** Comparisons of methylation level of *MPPED2* in different stages and locations of CRC tissues in the validation phase. (TIF 628 kb)

Additional file 4: **Table S3.** Results of ROC curve analysis between CRC tissues and adjacent normal tissues. (DOCX 12 kb)

## Abbreviations

27 K: Illumina 27 K BeadChip
AD: Adenoma
AD-N: Adenoma surrounding normal tissue
AJCC: American Joint Committee on Cancer
AUC: Area under curve
BMIQ: Beta-mixture quantile normalization
CA: Primary CRC
CA-N: CRC surrounding normal tissue
CGI: CpG island
CRC: Colorectal cancer
EPIC: Illumina MethylationEPIC BeadChip
FAP: Familial adenomatous polyposis
FDR: Adjusted *P* value with Benjamini-Hochberg method
HM450: Illumina HumanMethylation450 BeadChip
HP: Hyperplastic polyp
HP-N: Hyperplastic polyp surrounding normal tissue
HRN: High-risk normal tissue
IMA: Illumina Methylation Analyzer
LRN: Low-risk normal tissue
ORF: Open reading frame
RNA-Seq: RNA sequencing
ROC: Receiver operating characteristic
SD: Standard deviation
TCGA: The Cancer Genome Atlas
