# Supplementary material for: Genome-wide methylation profiling identified novel differentially hypermethylated biomarker MPPED2 in colorectal cancer
Source: Clin Epigenetics. 2019 Mar 7;11:41. doi: 10.1186/s13148-019-0628-y (PMC6407227; doi:10.1186/s13148-019-0628-y)
Supplement: Supplementary file 1 — Table S1. Pyrosequencing methylation assay Primers. (DOCX 13 kb) [file 13148_2019_628_MOESM1_ESM.docx]

**Table S1.** Pyrosequencing methylation assay Primers

|  |  | Primers |  | Sequence |  |
| --- | --- | --- | --- | --- | --- |
| MPPED2 |  | Forward |  | TTAGGATTTTTTAGGAGGAGAGGA | |
|  |  | Reverse |  | CAACACAATACACCCAAACCTA | |
|  |  | Sequencing |  | TTTAGAGAGTTGTTTATTAGAAAGT | |
| IKZF1 |  | Forward |  | TGAAAGAAAGTTGGGAAGAGT | |
|  |  | Reverse |  | AAACTCCCTCTACCCTACCAAA | |
|  |  | Sequencing |  | GTTAGTAGGATATTTTAATAAGTGA | |
| RSPO3 |  | Forward |  | GGAAGGGAAGTATTATTGGGTTAT | |
|  |  | Reverse |  | AAAAACCCTACACTTACTTCT | |
|  |  | Sequencing |  | TTTATTATTTTGAATTTTATGGAA | |
| COL23A1 |  | Forward |  | GTTTGGGTTGGGGAAGTT |  |
|  |  | Reverse |  | CCTCTATACAAATAAACCCTACCA | |
|  |  | Sequencing |  | GGTTGGGGAAGTTTTAGA |  |
| EPHA6 |  | Forward |  | AGGGGAATTGTATTTATTATAGTG | |
|  |  | Reverse |  | ACAACCCCTCCCCTACTTTTAACT | |
|  |  | Sequencing |  | GGGGAGGGTAGTTGG | |
