# Supplementary material for: Genome-wide methylation profiling identified novel differentially hypermethylated biomarker MPPED2 in colorectal cancer
Source: Clin Epigenetics. 2019 Mar 7;11:41. doi: 10.1186/s13148-019-0628-y (PMC6407227; doi:10.1186/s13148-019-0628-y)
Supplement: Supplementary file 2 — Table S2. A list of differentially hypermethylated and expressed genes identified by both EPIC array and TCGA database. (DOCX 173 kb) [file 13148_2019_628_MOESM2_ESM.docx]

**Table S2.** A list of differentially hypermethylated and expressed genes identified by both our EPIC array and TCGA database

| CpG ID |  | CHR |  | Location |  | Gene symbol |  | 850K dataset | | | | | | |  | TCGA dataset | | | | | | |
| --- | --- | --- | --- | --- | --- | --- | --- | --- | --- | --- | --- | --- | --- | --- | --- | --- | --- | --- | --- | --- | --- | --- |
|  |  |  |  |  |  |  |  | Mean % methylation | | | | | | |  | Methylation | | |  | Expression | | |
|  |  |  |  |  |  |  |  | CA |  | CA-N |  | Δβ |  | FDR |  | Δβ |  | FDR |  | FC |  | FDR |
| cg17872757 |  | 11 |  | 128564180 |  | FLI1 |  | 0.842 |  | 0.054 |  | 0.788 |  | 6.75E-06 |  | 0.610 |  | 1.41E-55 |  | -1.298 |  | 1.09E-29 |
| cg10471437 |  | 16 |  | 28074462 |  | GSG1L |  | 0.816 |  | 0.037 |  | 0.779 |  | 5.19E-06 |  | 0.510 |  | 1.09E-28 |  | -2.813 |  | 1.91E-35 |
| cg11017065 |  | 11 |  | 128564874 |  | FLI1 |  | 0.839 |  | 0.074 |  | 0.765 |  | 8.46E-06 |  | 0.677 |  | 5.00E-64 |  | -1.298 |  | 1.09E-29 |
| cg11855526 |  | 11 |  | 30607068 |  | MPPED2 |  | 0.785 |  | 0.065 |  | 0.720 |  | 1.00E-05 |  | 0.519 |  | 1.08E-23 |  | -2.068 |  | 1.94E-34 |
| cg06995920 |  | 12 |  | 64062544 |  | DPY19L2 |  | 0.791 |  | 0.075 |  | 0.716 |  | 1.90E-05 |  | 0.498 |  | 4.31E-34 |  | -1.277 |  | 3.73E-10 |
| cg23977631 |  | 2 |  | 100938799 |  | LONRF2 |  | 0.782 |  | 0.076 |  | 0.706 |  | 9.34E-06 |  | 0.492 |  | 1.91E-56 |  | -3.768 |  | 7.89E-60 |
| cg04455164 |  | 12 |  | 64062564 |  | DPY19L2 |  | 0.742 |  | 0.039 |  | 0.704 |  | 4.13E-05 |  | 0.552 |  | 2.45E-33 |  | -1.277 |  | 3.73E-10 |
| cg13879483 |  | 12 |  | 95942907 |  | USP44 |  | 0.858 |  | 0.157 |  | 0.701 |  | 1.52E-05 |  | 0.653 |  | 1.12E-49 |  | -1.156 |  | 1.08E-10 |
| cg08996579 |  | 10 |  | 50817804 |  | SLC18A3 |  | 0.824 |  | 0.130 |  | 0.694 |  | 3.49E-05 |  | 0.470 |  | 7.27E-38 |  | -2.734 |  | 1.25E-18 |
| cg18683604 |  | 14 |  | 51561293 |  | TRIM9 |  | 0.746 |  | 0.062 |  | 0.684 |  | 5.44E-05 |  | 0.431 |  | 7.68E-17 |  | -2.341 |  | 8.97E-41 |
| cg05203877 |  | 14 |  | 70655686 |  | SLC8A3 |  | 0.721 |  | 0.038 |  | 0.683 |  | 2.34E-05 |  | 0.373 |  | 3.11E-17 |  | -1.531 |  | 2.95E-11 |
| cg20078466 |  | 7 |  | 50344331 |  | IKZF1 |  | 0.709 |  | 0.028 |  | 0.681 |  | 1.41E-05 |  | 0.438 |  | 5.11E-22 |  | -1.553 |  | 4.62E-27 |
| cg23207710 |  | 3 |  | 192126960 |  | FGF12 |  | 0.792 |  | 0.113 |  | 0.679 |  | 2.16E-05 |  | 0.469 |  | 1.93E-48 |  | -1.036 |  | 1.80E-10 |
| cg06072021 |  | 11 |  | 128564106 |  | FLI1 |  | 0.723 |  | 0.050 |  | 0.673 |  | 1.01E-04 |  | 0.568 |  | 2.32E-46 |  | -1.298 |  | 1.09E-29 |
| cg22538054 |  | 12 |  | 95941988 |  | USP44 |  | 0.731 |  | 0.060 |  | 0.671 |  | 1.40E-04 |  | 0.515 |  | 2.60E-35 |  | -1.156 |  | 1.08E-10 |
| cg06204922 |  | 12 |  | 5019933 |  | KCNA1 |  | 0.781 |  | 0.114 |  | 0.668 |  | 4.81E-05 |  | 0.449 |  | 1.41E-38 |  | -3.896 |  | 4.28E-50 |
| cg23931421 |  | 6 |  | 152957999 |  | SYNE1 |  | 0.727 |  | 0.059 |  | 0.668 |  | 1.80E-05 |  | 0.533 |  | 2.59E-34 |  | -1.507 |  | 6.57E-25 |
| cg03921753 |  | 16 |  | 28074670 |  | GSG1L |  | 0.745 |  | 0.079 |  | 0.667 |  | 1.30E-05 |  | 0.442 |  | 1.24E-39 |  | -2.813 |  | 1.91E-35 |
| cg19897940 |  | 11 |  | 128563685 |  | FLI1 |  | 0.725 |  | 0.059 |  | 0.666 |  | 1.03E-04 |  | 0.525 |  | 1.30E-38 |  | -1.298 |  | 1.09E-29 |
| cg00020052 |  | 2 |  | 68546899 |  | CNRIP1 |  | 0.822 |  | 0.156 |  | 0.666 |  | 2.49E-05 |  | 0.410 |  | 1.32E-48 |  | -1.478 |  | 1.55E-40 |
| cg07279933 |  | 6 |  | 127440413 |  | RSPO3 |  | 0.702 |  | 0.036 |  | 0.665 |  | 2.86E-05 |  | 0.430 |  | 7.15E-22 |  | -1.395 |  | 7.07E-11 |
| cg01950845 |  | 15 |  | 93632730 |  | RGMA |  | 0.722 |  | 0.059 |  | 0.663 |  | 1.60E-05 |  | 0.448 |  | 3.00E-23 |  | -2.430 |  | 1.53E-49 |
| cg11666087 |  | 1 |  | 49242513 |  | BEND5 |  | 0.701 |  | 0.038 |  | 0.663 |  | 8.82E-05 |  | 0.505 |  | 1.78E-30 |  | -2.571 |  | 3.20E-76 |
| cg23987444 |  | 10 |  | 50817810 |  | SLC18A3 |  | 0.770 |  | 0.115 |  | 0.654 |  | 3.28E-05 |  | 0.566 |  | 5.34E-43 |  | -2.734 |  | 1.25E-18 |
| cg01588438 |  | 8 |  | 67344553 |  | ADHFE1 |  | 0.800 |  | 0.146 |  | 0.653 |  | 9.17E-05 |  | 0.630 |  | 2.28E-99 |  | -2.859 |  | 1.19E-133 |
| cg18065361 |  | 8 |  | 67344588 |  | ADHFE1 |  | 0.709 |  | 0.055 |  | 0.653 |  | 1.37E-04 |  | 0.605 |  | 1.04E-66 |  | -2.859 |  | 1.19E-133 |
| cg13916740 |  | 19 |  | 56904997 |  | ZNF582 |  | 0.755 |  | 0.102 |  | 0.653 |  | 2.59E-05 |  | 0.483 |  | 2.73E-31 |  | -1.345 |  | 5.51E-20 |
| cg25437410 |  | 5 |  | 178017578 |  | COL23A1 |  | 0.713 |  | 0.060 |  | 0.653 |  | 1.16E-04 |  | 0.436 |  | 2.32E-19 |  | -1.089 |  | 4.67E-14 |
| cg19283840 |  | 8 |  | 67344642 |  | ADHFE1 |  | 0.789 |  | 0.143 |  | 0.646 |  | 8.98E-05 |  | 0.509 |  | 2.07E-75 |  | -2.859 |  | 1.19E-133 |
| cg19868631 |  | 7 |  | 54609776 |  | VSTM2A |  | 0.827 |  | 0.184 |  | 0.643 |  | 2.16E-05 |  | 0.454 |  | 3.01E-34 |  | -5.178 |  | 1.84E-132 |
| cg17859110 |  | 20 |  | 41818770 |  | PTPRT |  | 0.741 |  | 0.098 |  | 0.643 |  | 2.40E-05 |  | 0.579 |  | 6.52E-39 |  | -1.704 |  | 3.19E-16 |
| cg27304110 |  | 1 |  | 114695695 |  | SYT6 |  | 0.723 |  | 0.082 |  | 0.640 |  | 4.59E-05 |  | 0.445 |  | 2.34E-26 |  | -1.841 |  | 2.36E-13 |
| cg18848688 |  | 5 |  | 38557386 |  | LIFR |  | 0.711 |  | 0.071 |  | 0.640 |  | 1.19E-04 |  | 0.415 |  | 9.46E-21 |  | -3.382 |  | 6.98E-134 |
| cg25884711 |  | 7 |  | 24323840 |  | NPY |  | 0.781 |  | 0.142 |  | 0.639 |  | 2.72E-05 |  | 0.483 |  | 1.71E-51 |  | -2.903 |  | 1.18E-30 |
| cg16848116 |  | 19 |  | 57862627 |  | ZNF304 |  | 0.686 |  | 0.049 |  | 0.638 |  | 1.17E-04 |  | 0.430 |  | 1.12E-25 |  | -1.412 |  | 1.49E-19 |
| cg10571951 |  | 5 |  | 88185387 |  | MEF2C |  | 0.666 |  | 0.030 |  | 0.637 |  | 3.65E-05 |  | 0.409 |  | 1.26E-25 |  | -1.622 |  | 1.65E-42 |
| cg06039355 |  | 10 |  | 118032033 |  | GFRA1 |  | 0.727 |  | 0.093 |  | 0.634 |  | 1.07E-04 |  | 0.410 |  | 1.19E-31 |  | -3.175 |  | 2.49E-85 |
| cg07748540 |  | 11 |  | 104034619 |  | PDGFD |  | 0.645 |  | 0.011 |  | 0.633 |  | 2.74E-04 |  | 0.506 |  | 6.81E-26 |  | -1.660 |  | 5.75E-43 |
| cg07783282 |  | 12 |  | 95942964 |  | USP44 |  | 0.726 |  | 0.095 |  | 0.631 |  | 5.38E-05 |  | 0.457 |  | 1.40E-33 |  | -1.156 |  | 1.08E-10 |
| cg00927554 |  | 12 |  | 95941920 |  | USP44 |  | 0.723 |  | 0.092 |  | 0.631 |  | 1.09E-04 |  | 0.504 |  | 1.60E-33 |  | -1.156 |  | 1.08E-10 |
| cg02216246 |  | 11 |  | 128564756 |  | FLI1 |  | 0.829 |  | 0.199 |  | 0.631 |  | 3.23E-05 |  | 0.397 |  | 6.83E-49 |  | -1.298 |  | 1.09E-29 |
| cg13755070 |  | 11 |  | 128563714 |  | FLI1 |  | 0.700 |  | 0.070 |  | 0.630 |  | 5.67E-04 |  | 0.596 |  | 2.49E-38 |  | -1.298 |  | 1.09E-29 |
| cg09383816 |  | 8 |  | 67344556 |  | ADHFE1 |  | 0.803 |  | 0.175 |  | 0.628 |  | 8.35E-05 |  | 0.606 |  | 2.58E-86 |  | -2.859 |  | 1.19E-133 |
| cg13346013 |  | 10 |  | 118032872 |  | GFRA1 |  | 0.705 |  | 0.077 |  | 0.628 |  | 1.48E-04 |  | 0.428 |  | 4.76E-30 |  | -3.175 |  | 2.49E-85 |
| cg05766140 |  | 6 |  | 391743 |  | IRF4 |  | 0.681 |  | 0.053 |  | 0.627 |  | 5.51E-05 |  | 0.497 |  | 1.70E-48 |  | -2.419 |  | 2.40E-49 |
| cg10566121 |  | 11 |  | 30606026 |  | MPPED2 |  | 0.753 |  | 0.126 |  | 0.627 |  | 2.12E-05 |  | 0.580 |  | 2.48E-38 |  | -2.068 |  | 1.94E-34 |
| cg12587766 |  | 5 |  | 38556435 |  | LIFR |  | 0.694 |  | 0.067 |  | 0.627 |  | 1.43E-04 |  | 0.562 |  | 1.06E-36 |  | -3.382 |  | 6.98E-134 |
| cg15093079 |  | 3 |  | 96533290 |  | EPHA6 |  | 0.701 |  | 0.074 |  | 0.626 |  | 1.58E-04 |  | 0.390 |  | 1.43E-13 |  | -4.332 |  | 2.42E-78 |
| cg09008705 |  | 1 |  | 115880865 |  | NGF |  | 0.773 |  | 0.151 |  | 0.622 |  | 4.73E-05 |  | 0.527 |  | 6.09E-41 |  | -1.183 |  | 1.84E-11 |
| cg19039028 |  | 4 |  | 110223429 |  | COL25A1 |  | 0.800 |  | 0.178 |  | 0.621 |  | 6.71E-05 |  | 0.443 |  | 1.05E-42 |  | -1.087 |  | 1.63E-05 |
| cg00843236 |  | 16 |  | 28074980 |  | GSG1L |  | 0.660 |  | 0.042 |  | 0.619 |  | 2.28E-04 |  | 0.403 |  | 2.66E-12 |  | -2.813 |  | 1.91E-35 |
| cg20409590 |  | 14 |  | 70655382 |  | SLC8A3 |  | 0.700 |  | 0.082 |  | 0.618 |  | 2.30E-05 |  | 0.430 |  | 2.19E-20 |  | -1.531 |  | 2.95E-11 |
| cg18672939 |  | 10 |  | 118032879 |  | GFRA1 |  | 0.705 |  | 0.089 |  | 0.616 |  | 2.06E-04 |  | 0.555 |  | 4.01E-34 |  | -3.175 |  | 2.49E-85 |
| cg08190044 |  | 1 |  | 154298335 |  | ATP8B2 |  | 0.735 |  | 0.124 |  | 0.612 |  | 1.65E-04 |  | 0.442 |  | 6.50E-34 |  | -1.075 |  | 9.49E-17 |
| cg03976877 |  | 7 |  | 158937610 |  | VIPR2 |  | 0.854 |  | 0.243 |  | 0.611 |  | 2.16E-05 |  | 0.592 |  | 6.36E-47 |  | -2.010 |  | 4.28E-32 |
| cg17904997 |  | 19 |  | 44952572 |  | ZNF229 |  | 0.709 |  | 0.099 |  | 0.610 |  | 7.64E-05 |  | 0.411 |  | 3.00E-27 |  | -2.137 |  | 6.37E-44 |
| cg11354906 |  | 4 |  | 154710371 |  | SFRP2 |  | 0.715 |  | 0.105 |  | 0.610 |  | 7.00E-05 |  | 0.418 |  | 3.44E-29 |  | -1.139 |  | 6.63E-05 |
| cg16573178 |  | 1 |  | 49242519 |  | BEND5 |  | 0.644 |  | 0.035 |  | 0.610 |  | 2.71E-04 |  | 0.490 |  | 1.16E-26 |  | -2.571 |  | 3.20E-76 |
| cg11430157 |  | 6 |  | 118228869 |  | SLC35F1 |  | 0.656 |  | 0.046 |  | 0.609 |  | 1.15E-04 |  | 0.451 |  | 9.97E-22 |  | -2.178 |  | 8.65E-52 |
| cg03308628 |  | 12 |  | 95942287 |  | USP44 |  | 0.658 |  | 0.049 |  | 0.609 |  | 8.85E-05 |  | 0.391 |  | 9.31E-32 |  | -1.156 |  | 1.08E-10 |
| cg16964348 |  | 7 |  | 24323799 |  | NPY |  | 0.825 |  | 0.216 |  | 0.608 |  | 3.78E-05 |  | 0.483 |  | 2.33E-52 |  | -2.903 |  | 1.18E-30 |
| cg01893212 |  | 7 |  | 49813088 |  | VWC2 |  | 0.686 |  | 0.078 |  | 0.608 |  | 3.91E-04 |  | 0.566 |  | 7.58E-29 |  | -2.201 |  | 5.96E-27 |
| cg19166660 |  | 1 |  | 57889035 |  | DAB1 |  | 0.769 |  | 0.162 |  | 0.607 |  | 4.66E-05 |  | 0.487 |  | 1.26E-33 |  | -1.050 |  | 9.62E-06 |
| cg15929698 |  | 7 |  | 24323792 |  | NPY |  | 0.848 |  | 0.241 |  | 0.607 |  | 3.65E-05 |  | 0.355 |  | 1.44E-44 |  | -2.903 |  | 1.18E-30 |
| cg10172415 |  | 3 |  | 71803558 |  | GPR27 |  | 0.739 |  | 0.133 |  | 0.606 |  | 1.06E-04 |  | 0.433 |  | 3.26E-19 |  | -1.629 |  | 1.55E-12 |
| cg07080358 |  | 2 |  | 68546507 |  | CNRIP1 |  | 0.733 |  | 0.128 |  | 0.605 |  | 3.49E-05 |  | 0.436 |  | 4.81E-55 |  | -1.478 |  | 1.55E-40 |
| cg24171907 |  | 2 |  | 68546579 |  | CNRIP1 |  | 0.747 |  | 0.142 |  | 0.605 |  | 2.66E-05 |  | 0.479 |  | 4.74E-69 |  | -1.478 |  | 1.55E-40 |
| cg07808555 |  | 1 |  | 111217712 |  | KCNA3 |  | 0.788 |  | 0.186 |  | 0.602 |  | 4.22E-05 |  | 0.472 |  | 2.39E-41 |  | -2.192 |  | 3.66E-35 |
| cg14063488 |  | 4 |  | 154709878 |  | SFRP2 |  | 0.649 |  | 0.047 |  | 0.602 |  | 7.43E-04 |  | 0.432 |  | 1.18E-22 |  | -1.139 |  | 6.63E-05 |
| cg04904331 |  | 7 |  | 49813033 |  | VWC2 |  | 0.692 |  | 0.092 |  | 0.600 |  | 4.49E-04 |  | 0.547 |  | 3.60E-27 |  | -2.201 |  | 5.96E-27 |
| cg13491690 |  | 11 |  | 134145979 |  | GLB1L3 |  | 0.640 |  | 0.040 |  | 0.599 |  | 1.44E-04 |  | 0.418 |  | 1.13E-19 |  | -1.278 |  | 8.38E-06 |
| cg23437733 |  | 5 |  | 178017571 |  | COL23A1 |  | 0.674 |  | 0.076 |  | 0.598 |  | 2.82E-04 |  | 0.443 |  | 1.04E-20 |  | -1.089 |  | 4.67E-14 |
| cg17256532 |  | 10 |  | 118031950 |  | GFRA1 |  | 0.664 |  | 0.067 |  | 0.597 |  | 1.17E-04 |  | 0.445 |  | 1.57E-27 |  | -3.175 |  | 2.49E-85 |
| cg19485539 |  | 10 |  | 118031870 |  | GFRA1 |  | 0.689 |  | 0.093 |  | 0.597 |  | 1.37E-04 |  | 0.533 |  | 4.73E-47 |  | -3.175 |  | 2.49E-85 |
| cg18607529 |  | 7 |  | 50343869 |  | IKZF1 |  | 0.636 |  | 0.040 |  | 0.596 |  | 1.47E-05 |  | 0.399 |  | 1.55E-24 |  | -1.553 |  | 4.62E-27 |
| cg17108819 |  | 2 |  | 87017953 |  | CD8A |  | 0.741 |  | 0.145 |  | 0.596 |  | 1.28E-05 |  | 0.385 |  | 4.65E-28 |  | -1.091 |  | 1.35E-09 |
| cg13265789 |  | 4 |  | 96470584 |  | UNC5C |  | 0.757 |  | 0.162 |  | 0.596 |  | 6.64E-05 |  | 0.499 |  | 3.42E-44 |  | -2.323 |  | 6.43E-92 |
| cg23209990 |  | 4 |  | 55097576 |  | PDGFRA |  | 0.762 |  | 0.167 |  | 0.595 |  | 1.52E-04 |  | 0.473 |  | 1.41E-30 |  | -1.185 |  | 1.63E-19 |
| cg03202804 |  | 4 |  | 154710353 |  | SFRP2 |  | 0.713 |  | 0.118 |  | 0.595 |  | 6.31E-05 |  | 0.467 |  | 1.86E-30 |  | -1.139 |  | 6.63E-05 |
| cg27264249 |  | 3 |  | 2141686 |  | CNTN4 |  | 0.713 |  | 0.120 |  | 0.593 |  | 7.08E-05 |  | 0.452 |  | 1.45E-30 |  | -2.476 |  | 8.28E-80 |
| cg11573679 |  | 2 |  | 68546467 |  | CNRIP1 |  | 0.651 |  | 0.063 |  | 0.589 |  | 4.08E-05 |  | 0.497 |  | 3.86E-54 |  | -1.478 |  | 1.55E-40 |
| cg25230074 |  | 8 |  | 32405792 |  | NRG1 |  | 0.755 |  | 0.166 |  | 0.588 |  | 2.70E-04 |  | 0.461 |  | 7.11E-22 |  | -1.447 |  | 5.33E-16 |
| cg02450613 |  | 5 |  | 37840089 |  | GDNF |  | 0.679 |  | 0.091 |  | 0.588 |  | 8.82E-05 |  | 0.428 |  | 7.33E-29 |  | -2.297 |  | 8.35E-67 |
| cg08521987 |  | 10 |  | 119000927 |  | SLC18A2 |  | 0.780 |  | 0.193 |  | 0.587 |  | 6.73E-05 |  | 0.451 |  | 2.22E-20 |  | -1.308 |  | 5.72E-23 |
| cg11398511 |  | 1 |  | 215256635 |  | KCNK2 |  | 0.665 |  | 0.079 |  | 0.586 |  | 2.33E-04 |  | 0.383 |  | 1.82E-12 |  | -2.107 |  | 1.68E-18 |
| cg10511904 |  | 3 |  | 192127327 |  | FGF12 |  | 0.702 |  | 0.116 |  | 0.586 |  | 4.73E-05 |  | 0.404 |  | 1.54E-51 |  | -1.036 |  | 1.80E-10 |
| cg20912169 |  | 8 |  | 67344720 |  | ADHFE1 |  | 0.737 |  | 0.151 |  | 0.586 |  | 1.87E-04 |  | 0.649 |  | 3.08E-88 |  | -2.859 |  | 1.19E-133 |
| cg00421139 |  | 8 |  | 97172961 |  | GDF6 |  | 0.705 |  | 0.119 |  | 0.585 |  | 3.99E-05 |  | 0.551 |  | 8.64E-71 |  | -1.283 |  | 9.70E-08 |
| cg18023283 |  | 12 |  | 85306828 |  | SLC6A15 |  | 0.853 |  | 0.268 |  | 0.585 |  | 2.65E-05 |  | 0.586 |  | 3.27E-52 |  | -1.371 |  | 1.55E-04 |
| cg18044663 |  | 10 |  | 118032081 |  | GFRA1 |  | 0.733 |  | 0.148 |  | 0.585 |  | 1.39E-04 |  | 0.508 |  | 6.61E-38 |  | -3.175 |  | 2.49E-85 |
| cg20041366 |  | 12 |  | 62586267 |  | FAM19A2 |  | 0.845 |  | 0.261 |  | 0.585 |  | 2.44E-05 |  | 0.433 |  | 2.77E-34 |  | -1.128 |  | 4.53E-15 |
| cg27028555 |  | 11 |  | 128563027 |  | FLI1 |  | 0.660 |  | 0.076 |  | 0.584 |  | 7.79E-05 |  | 0.489 |  | 1.93E-41 |  | -1.298 |  | 1.09E-29 |
| cg20984085 |  | 19 |  | 56905032 |  | ZNF582 |  | 0.663 |  | 0.080 |  | 0.583 |  | 6.84E-05 |  | 0.424 |  | 1.26E-31 |  | -1.345 |  | 5.51E-20 |
| cg03060201 |  | 19 |  | 37407374 |  | ZNF568 |  | 0.626 |  | 0.044 |  | 0.583 |  | 3.44E-04 |  | 0.454 |  | 5.71E-29 |  | -1.326 |  | 1.57E-19 |
| cg03384825 |  | 1 |  | 57889604 |  | DAB1 |  | 0.729 |  | 0.146 |  | 0.583 |  | 8.93E-05 |  | 0.518 |  | 1.54E-34 |  | -1.050 |  | 9.62E-06 |
| cg19944763 |  | 10 |  | 15761854 |  | ITGA8 |  | 0.731 |  | 0.152 |  | 0.579 |  | 6.28E-05 |  | 0.471 |  | 5.40E-39 |  | -1.870 |  | 4.80E-60 |
| cg19365062 |  | 6 |  | 127440000 |  | RSPO3 |  | 0.623 |  | 0.045 |  | 0.578 |  | 4.98E-04 |  | 0.485 |  | 1.74E-17 |  | -1.395 |  | 7.07E-11 |
| cg06298519 |  | 10 |  | 118031864 |  | GFRA1 |  | 0.673 |  | 0.095 |  | 0.578 |  | 1.97E-04 |  | 0.535 |  | 4.91E-48 |  | -3.175 |  | 2.49E-85 |
| cg20219457 |  | 13 |  | 36705446 |  | DCLK1 |  | 0.702 |  | 0.125 |  | 0.578 |  | 5.80E-05 |  | 0.595 |  | 3.81E-52 |  | -2.886 |  | 5.36E-68 |
| cg22723056 |  | 11 |  | 7273378 |  | SYT9 |  | 0.679 |  | 0.101 |  | 0.578 |  | 3.90E-05 |  | 0.430 |  | 5.68E-30 |  | -2.647 |  | 2.49E-31 |
| cg17228900 |  | 6 |  | 391764 |  | IRF4 |  | 0.627 |  | 0.050 |  | 0.577 |  | 9.36E-05 |  | 0.544 |  | 9.37E-47 |  | -2.419 |  | 2.40E-49 |
| cg03503087 |  | 10 |  | 118032626 |  | GFRA1 |  | 0.637 |  | 0.062 |  | 0.575 |  | 2.55E-04 |  | 0.431 |  | 4.77E-29 |  | -3.175 |  | 2.49E-85 |
| cg05791136 |  | 4 |  | 110223969 |  | COL25A1 |  | 0.686 |  | 0.111 |  | 0.575 |  | 4.47E-05 |  | 0.477 |  | 1.21E-46 |  | -1.087 |  | 1.63E-05 |
| cg02467990 |  | 7 |  | 49813102 |  | VWC2 |  | 0.710 |  | 0.135 |  | 0.575 |  | 8.27E-04 |  | 0.556 |  | 1.45E-29 |  | -2.201 |  | 5.96E-27 |
| cg09410234 |  | 1 |  | 111217715 |  | KCNA3 |  | 0.783 |  | 0.209 |  | 0.575 |  | 3.51E-05 |  | 0.389 |  | 1.76E-41 |  | -2.192 |  | 3.66E-35 |
| cg03419885 |  | 11 |  | 125036385 |  | PKNOX2 |  | 0.668 |  | 0.094 |  | 0.573 |  | 1.47E-04 |  | 0.485 |  | 3.70E-34 |  | -2.749 |  | 1.20E-86 |
| cg08234308 |  | 1 |  | 65991176 |  | LEPR |  | 0.843 |  | 0.270 |  | 0.573 |  | 4.41E-05 |  | 0.459 |  | 2.53E-21 |  | -1.466 |  | 6.17E-31 |
| cg20560075 |  | 5 |  | 146257484 |  | PPP2R2B |  | 0.641 |  | 0.068 |  | 0.573 |  | 4.04E-05 |  | 0.433 |  | 4.25E-25 |  | -2.147 |  | 4.81E-42 |
| cg03735888 |  | 19 |  | 58951602 |  | ZNF132 |  | 0.605 |  | 0.033 |  | 0.572 |  | 5.76E-04 |  | 0.506 |  | 1.07E-30 |  | -1.161 |  | 1.21E-40 |
| cg20959325 |  | 11 |  | 30606165 |  | MPPED2 |  | 0.672 |  | 0.100 |  | 0.572 |  | 1.44E-04 |  | 0.459 |  | 2.08E-31 |  | -2.068 |  | 1.94E-34 |
| cg05937969 |  | 3 |  | 121903016 |  | CASR |  | 0.650 |  | 0.079 |  | 0.572 |  | 4.75E-05 |  | 0.495 |  | 8.14E-40 |  | -3.375 |  | 1.14E-61 |
| cg04529785 |  | 12 |  | 117799083 |  | NOS1 |  | 0.685 |  | 0.113 |  | 0.571 |  | 7.90E-05 |  | 0.441 |  | 2.85E-38 |  | -3.377 |  | 5.07E-46 |
| cg09926747 |  | 4 |  | 110224247 |  | COL25A1 |  | 0.758 |  | 0.188 |  | 0.571 |  | 4.03E-05 |  | 0.434 |  | 8.54E-46 |  | -1.087 |  | 1.63E-05 |
| cg20295442 |  | 8 |  | 67344665 |  | ADHFE1 |  | 0.749 |  | 0.178 |  | 0.571 |  | 1.27E-04 |  | 0.671 |  | 1.91E-92 |  | -2.859 |  | 1.19E-133 |
| cg22823146 |  | 3 |  | 71802594 |  | EIF4E3 |  | 0.674 |  | 0.103 |  | 0.571 |  | 2.58E-04 |  | 0.420 |  | 2.66E-24 |  | -1.885 |  | 8.61E-74 |
| cg21692846 |  | 11 |  | 134146075 |  | GLB1L3 |  | 0.603 |  | 0.033 |  | 0.570 |  | 1.08E-04 |  | 0.434 |  | 3.37E-26 |  | -1.278 |  | 8.38E-06 |
| cg26036626 |  | 1 |  | 16085597 |  | FBLIM1 |  | 0.764 |  | 0.195 |  | 0.569 |  | 8.74E-05 |  | 0.601 |  | 6.08E-63 |  | -1.290 |  | 1.45E-80 |
| cg18710929 |  | 6 |  | 118228078 |  | SLC35F1 |  | 0.644 |  | 0.075 |  | 0.569 |  | 4.68E-04 |  | 0.415 |  | 1.43E-15 |  | -2.178 |  | 8.65E-52 |
| cg11540007 |  | 2 |  | 145274975 |  | ZEB2 |  | 0.602 |  | 0.034 |  | 0.568 |  | 3.71E-05 |  | 0.378 |  | 6.16E-22 |  | -1.416 |  | 1.89E-21 |
| cg19236679 |  | 10 |  | 118032330 |  | GFRA1 |  | 0.638 |  | 0.072 |  | 0.565 |  | 2.23E-04 |  | 0.444 |  | 2.55E-26 |  | -3.175 |  | 2.49E-85 |
| cg11008243 |  | 6 |  | 73331238 |  | KCNQ5 |  | 0.666 |  | 0.102 |  | 0.564 |  | 2.72E-05 |  | 0.449 |  | 2.19E-42 |  | -2.055 |  | 1.10E-17 |
| cg01295518 |  | 3 |  | 238392 |  | CHL1 |  | 0.675 |  | 0.111 |  | 0.564 |  | 1.17E-04 |  | 0.469 |  | 6.54E-30 |  | -2.660 |  | 2.33E-61 |
| cg10784386 |  | 5 |  | 79330943 |  | THBS4 |  | 0.901 |  | 0.339 |  | 0.562 |  | 3.44E-05 |  | 0.485 |  | 1.22E-32 |  | -2.351 |  | 2.25E-20 |
| cg12666279 |  | 2 |  | 115918745 |  | DPP10 |  | 0.724 |  | 0.162 |  | 0.562 |  | 1.85E-04 |  | 0.500 |  | 5.63E-28 |  | -1.786 |  | 2.51E-08 |
| cg21902544 |  | 18 |  | 70211515 |  | CBLN2 |  | 0.767 |  | 0.205 |  | 0.562 |  | 5.13E-05 |  | 0.459 |  | 2.76E-41 |  | -2.950 |  | 6.25E-75 |
| cg13281139 |  | 4 |  | 20255061 |  | SLIT2 |  | 0.775 |  | 0.213 |  | 0.561 |  | 3.47E-05 |  | 0.498 |  | 1.85E-47 |  | -1.978 |  | 3.90E-26 |
| cg15048660 |  | 3 |  | 192126935 |  | FGF12 |  | 0.646 |  | 0.084 |  | 0.561 |  | 5.21E-05 |  | 0.493 |  | 2.85E-42 |  | -1.036 |  | 1.80E-10 |
| cg04765848 |  | 1 |  | 53527576 |  | PODN |  | 0.751 |  | 0.191 |  | 0.561 |  | 2.72E-04 |  | 0.397 |  | 2.29E-23 |  | -1.062 |  | 2.60E-10 |
| cg12741420 |  | 6 |  | 392131 |  | IRF4 |  | 0.740 |  | 0.181 |  | 0.559 |  | 2.72E-05 |  | 0.384 |  | 6.24E-59 |  | -2.419 |  | 2.40E-49 |
| cg09239744 |  | 16 |  | 10276580 |  | GRIN2A |  | 0.608 |  | 0.050 |  | 0.559 |  | 1.87E-04 |  | 0.437 |  | 2.42E-27 |  | -2.691 |  | 6.46E-32 |
| cg16366473 |  | 3 |  | 192126849 |  | FGF12 |  | 0.638 |  | 0.079 |  | 0.558 |  | 3.92E-05 |  | 0.509 |  | 3.16E-43 |  | -1.036 |  | 1.80E-10 |
| cg16376000 |  | 3 |  | 192127330 |  | FGF12 |  | 0.723 |  | 0.165 |  | 0.558 |  | 4.82E-05 |  | 0.477 |  | 7.14E-66 |  | -1.036 |  | 1.80E-10 |
| cg27648738 |  | 15 |  | 84115811 |  | SH3GL3 |  | 0.700 |  | 0.144 |  | 0.557 |  | 7.70E-05 |  | 0.486 |  | 1.06E-26 |  | -2.120 |  | 4.81E-11 |
| cg14675211 |  | 2 |  | 100938903 |  | LONRF2 |  | 0.605 |  | 0.049 |  | 0.556 |  | 2.79E-05 |  | 0.432 |  | 2.81E-56 |  | -3.768 |  | 7.89E-60 |
| cg08090772 |  | 8 |  | 67344640 |  | ADHFE1 |  | 0.671 |  | 0.115 |  | 0.556 |  | 1.30E-04 |  | 0.504 |  | 2.06E-73 |  | -2.859 |  | 1.19E-133 |
| cg10363246 |  | 12 |  | 5019482 |  | KCNA1 |  | 0.600 |  | 0.044 |  | 0.556 |  | 1.79E-04 |  | 0.474 |  | 8.46E-32 |  | -3.896 |  | 4.28E-50 |
| cg05621343 |  | 11 |  | 134146253 |  | GLB1L3 |  | 0.708 |  | 0.153 |  | 0.555 |  | 1.88E-04 |  | 0.539 |  | 2.85E-33 |  | -1.278 |  | 8.38E-06 |
| cg25971347 |  | 16 |  | 86544339 |  | FOXF1 |  | 0.664 |  | 0.109 |  | 0.554 |  | 3.54E-04 |  | 0.394 |  | 7.26E-18 |  | -1.462 |  | 2.83E-36 |
| cg26215967 |  | 15 |  | 84115897 |  | SH3GL3 |  | 0.654 |  | 0.101 |  | 0.553 |  | 2.68E-04 |  | 0.377 |  | 1.00E-17 |  | -2.120 |  | 4.81E-11 |
| cg21688264 |  | 6 |  | 84418724 |  | SNAP91 |  | 0.773 |  | 0.221 |  | 0.552 |  | 6.77E-04 |  | 0.445 |  | 1.05E-37 |  | -3.160 |  | 4.28E-42 |
| cg17846334 |  | 20 |  | 49639134 |  | KCNG1 |  | 0.665 |  | 0.113 |  | 0.552 |  | 2.56E-05 |  | 0.424 |  | 4.87E-27 |  | -1.329 |  | 5.33E-08 |
| cg00343633 |  | 11 |  | 105481306 |  | GRIA4 |  | 0.854 |  | 0.302 |  | 0.552 |  | 8.10E-05 |  | 0.530 |  | 1.41E-54 |  | -2.716 |  | 1.42E-52 |
| cg03443751 |  | 19 |  | 37407462 |  | ZNF829 |  | 0.612 |  | 0.060 |  | 0.552 |  | 6.51E-04 |  | 0.422 |  | 7.60E-18 |  | -1.193 |  | 1.72E-12 |
| cg08361126 |  | 10 |  | 15762091 |  | ITGA8 |  | 0.748 |  | 0.197 |  | 0.551 |  | 3.87E-05 |  | 0.407 |  | 6.27E-45 |  | -1.870 |  | 4.80E-60 |
| cg05447008 |  | 6 |  | 73331114 |  | KCNQ5 |  | 0.689 |  | 0.138 |  | 0.551 |  | 1.44E-04 |  | 0.499 |  | 4.59E-49 |  | -2.055 |  | 1.10E-17 |
| cg14135551 |  | 6 |  | 39760416 |  | DAAM2 |  | 0.626 |  | 0.076 |  | 0.549 |  | 7.94E-05 |  | 0.388 |  | 1.80E-17 |  | -1.924 |  | 4.59E-65 |
| cg25775322 |  | 4 |  | 154710373 |  | SFRP2 |  | 0.765 |  | 0.215 |  | 0.549 |  | 6.67E-05 |  | 0.391 |  | 2.05E-31 |  | -1.139 |  | 6.63E-05 |
| cg03563667 |  | 4 |  | 142054417 |  | RNF150 |  | 0.612 |  | 0.064 |  | 0.549 |  | 2.75E-04 |  | 0.379 |  | 1.85E-17 |  | -3.394 |  | 6.25E-98 |
| cg02916312 |  | 6 |  | 72596135 |  | RIMS1 |  | 0.591 |  | 0.044 |  | 0.548 |  | 4.77E-04 |  | 0.381 |  | 4.46E-16 |  | -1.053 |  | 2.13E-04 |
| cg08808128 |  | 2 |  | 29338432 |  | CLIP4 |  | 0.702 |  | 0.155 |  | 0.547 |  | 6.11E-05 |  | 0.478 |  | 3.24E-46 |  | -2.068 |  | 2.57E-41 |
| cg14159026 |  | 6 |  | 105584551 |  | BVES |  | 0.628 |  | 0.081 |  | 0.547 |  | 7.30E-04 |  | 0.479 |  | 2.85E-22 |  | -2.897 |  | 7.75E-98 |
| cg11979589 |  | 4 |  | 110223967 |  | COL25A1 |  | 0.686 |  | 0.139 |  | 0.546 |  | 5.18E-05 |  | 0.487 |  | 7.91E-52 |  | -1.087 |  | 1.63E-05 |
| cg02288301 |  | 2 |  | 193060811 |  | TMEFF2 |  | 0.687 |  | 0.141 |  | 0.546 |  | 6.21E-05 |  | 0.397 |  | 1.46E-22 |  | -4.134 |  | 3.79E-97 |
| cg09354309 |  | 12 |  | 22487437 |  | ST8SIA1 |  | 0.569 |  | 0.023 |  | 0.546 |  | 1.43E-04 |  | 0.383 |  | 1.05E-19 |  | -1.764 |  | 1.21E-37 |
| cg04883903 |  | 12 |  | 117798954 |  | NOS1 |  | 0.723 |  | 0.177 |  | 0.546 |  | 5.02E-05 |  | 0.550 |  | 6.35E-52 |  | -3.377 |  | 5.07E-46 |
| cg12602374 |  | 5 |  | 38557162 |  | LIFR |  | 0.603 |  | 0.057 |  | 0.546 |  | 3.49E-04 |  | 0.367 |  | 4.16E-23 |  | -3.382 |  | 6.98E-134 |
| cg14185025 |  | 14 |  | 100259341 |  | EML1 |  | 0.642 |  | 0.096 |  | 0.546 |  | 1.10E-04 |  | 0.396 |  | 1.97E-16 |  | -2.393 |  | 3.13E-87 |
| cg03323292 |  | 11 |  | 134146132 |  | GLB1L3 |  | 0.595 |  | 0.049 |  | 0.545 |  | 1.41E-04 |  | 0.465 |  | 2.74E-23 |  | -1.278 |  | 8.38E-06 |
| cg01419567 |  | 8 |  | 77594595 |  | ZFHX4 |  | 0.613 |  | 0.069 |  | 0.544 |  | 1.10E-04 |  | 0.474 |  | 3.95E-35 |  | -1.166 |  | 1.24E-06 |
| cg03654735 |  | 1 |  | 38100826 |  | RSPO1 |  | 0.678 |  | 0.136 |  | 0.543 |  | 8.10E-05 |  | 0.401 |  | 4.52E-20 |  | -2.063 |  | 2.33E-23 |
| cg08002883 |  | 3 |  | 192127457 |  | FGF12 |  | 0.647 |  | 0.104 |  | 0.543 |  | 5.62E-05 |  | 0.475 |  | 2.14E-65 |  | -1.036 |  | 1.80E-10 |
| cg05901579 |  | 15 |  | 88800567 |  | NTRK3 |  | 0.803 |  | 0.260 |  | 0.543 |  | 5.92E-05 |  | 0.391 |  | 9.17E-30 |  | -2.086 |  | 3.74E-20 |
| cg10928087 |  | 3 |  | 46923937 |  | PTH1R |  | 0.629 |  | 0.088 |  | 0.541 |  | 2.93E-04 |  | 0.356 |  | 1.39E-15 |  | -2.023 |  | 4.10E-35 |
| cg06943420 |  | 1 |  | 1566699 |  | MMP23A |  | 0.821 |  | 0.279 |  | 0.541 |  | 5.92E-05 |  | 0.469 |  | 6.42E-25 |  | -1.281 |  | 1.90E-05 |
| cg11960629 |  | 10 |  | 91295317 |  | SLC16A12 |  | 0.707 |  | 0.167 |  | 0.540 |  | 5.47E-05 |  | 0.401 |  | 2.13E-24 |  | -2.811 |  | 6.10E-71 |
| cg27296293 |  | 11 |  | 101454680 |  | TRPC6 |  | 0.789 |  | 0.249 |  | 0.540 |  | 4.49E-05 |  | 0.463 |  | 6.51E-49 |  | -1.238 |  | 2.67E-20 |
| cg18624636 |  | 10 |  | 91295358 |  | SLC16A12 |  | 0.603 |  | 0.063 |  | 0.540 |  | 2.89E-04 |  | 0.407 |  | 9.85E-20 |  | -2.811 |  | 6.10E-71 |
| cg01808545 |  | 2 |  | 193059717 |  | TMEFF2 |  | 0.585 |  | 0.045 |  | 0.540 |  | 1.26E-04 |  | 0.477 |  | 1.45E-27 |  | -4.134 |  | 3.79E-97 |
| cg10333808 |  | 12 |  | 22487459 |  | ST8SIA1 |  | 0.634 |  | 0.095 |  | 0.539 |  | 1.50E-04 |  | 0.422 |  | 1.04E-29 |  | -1.764 |  | 1.21E-37 |
| cg17368254 |  | 12 |  | 95942847 |  | USP44 |  | 0.621 |  | 0.082 |  | 0.539 |  | 2.67E-04 |  | 0.425 |  | 3.59E-29 |  | -1.156 |  | 1.08E-10 |
| cg07589773 |  | 7 |  | 50343883 |  | IKZF1 |  | 0.625 |  | 0.087 |  | 0.539 |  | 1.29E-05 |  | 0.478 |  | 1.96E-31 |  | -1.553 |  | 4.62E-27 |
| cg16818740 |  | 20 |  | 4803300 |  | RASSF2 |  | 0.597 |  | 0.058 |  | 0.538 |  | 2.69E-05 |  | 0.423 |  | 1.40E-33 |  | -1.261 |  | 7.80E-23 |
| cg03108229 |  | 11 |  | 134146266 |  | GLB1L3 |  | 0.639 |  | 0.102 |  | 0.537 |  | 2.21E-04 |  | 0.428 |  | 1.05E-30 |  | -1.278 |  | 8.38E-06 |
| cg14449051 |  | 12 |  | 85306474 |  | SLC6A15 |  | 0.669 |  | 0.132 |  | 0.537 |  | 7.39E-05 |  | 0.472 |  | 6.47E-37 |  | -1.371 |  | 1.55E-04 |
| cg09979256 |  | 6 |  | 127440104 |  | RSPO3 |  | 0.566 |  | 0.030 |  | 0.536 |  | 4.83E-04 |  | 0.379 |  | 2.53E-16 |  | -1.395 |  | 7.07E-11 |
| cg03382304 |  | 21 |  | 27012176 |  | JAM2 |  | 0.611 |  | 0.075 |  | 0.536 |  | 5.91E-05 |  | 0.428 |  | 2.29E-37 |  | -2.547 |  | 1.20E-92 |
| cg05375728 |  | 1 |  | 58715539 |  | DAB1 |  | 0.762 |  | 0.227 |  | 0.535 |  | 3.08E-05 |  | 0.406 |  | 4.05E-33 |  | -1.050 |  | 9.62E-06 |
| cg14348439 |  | 2 |  | 100938444 |  | LONRF2 |  | 0.647 |  | 0.112 |  | 0.535 |  | 2.69E-05 |  | 0.421 |  | 5.28E-54 |  | -3.768 |  | 7.89E-60 |
| cg02120582 |  | 11 |  | 104034754 |  | PDGFD |  | 0.574 |  | 0.040 |  | 0.535 |  | 1.18E-04 |  | 0.431 |  | 1.70E-22 |  | -1.660 |  | 5.75E-43 |
| cg24521633 |  | 4 |  | 166794786 |  | TLL1 |  | 0.595 |  | 0.061 |  | 0.535 |  | 1.05E-03 |  | 0.420 |  | 5.46E-17 |  | -2.406 |  | 3.96E-40 |
| cg02989521 |  | 11 |  | 107462437 |  | ELMOD1 |  | 0.630 |  | 0.095 |  | 0.535 |  | 1.02E-04 |  | 0.418 |  | 3.71E-17 |  | -1.406 |  | 6.52E-10 |
| cg07095995 |  | 4 |  | 110223980 |  | COL25A1 |  | 0.715 |  | 0.181 |  | 0.534 |  | 3.41E-05 |  | 0.470 |  | 9.29E-52 |  | -1.087 |  | 1.63E-05 |
| cg23559689 |  | 11 |  | 105481292 |  | GRIA4 |  | 0.846 |  | 0.312 |  | 0.534 |  | 1.55E-04 |  | 0.457 |  | 3.42E-50 |  | -2.716 |  | 1.42E-52 |
| cg08568720 |  | 20 |  | 61051432 |  | GATA5 |  | 0.669 |  | 0.136 |  | 0.534 |  | 1.12E-04 |  | 0.528 |  | 4.65E-27 |  | -2.304 |  | 5.49E-13 |
| cg03225817 |  | 11 |  | 105481317 |  | GRIA4 |  | 0.736 |  | 0.202 |  | 0.534 |  | 1.56E-04 |  | 0.468 |  | 5.33E-44 |  | -2.716 |  | 1.42E-52 |
| cg25943503 |  | 13 |  | 36705608 |  | DCLK1 |  | 0.708 |  | 0.175 |  | 0.533 |  | 6.88E-05 |  | 0.489 |  | 1.67E-34 |  | -2.886 |  | 5.36E-68 |
| cg04035209 |  | 4 |  | 134069670 |  | PCDH10 |  | 0.662 |  | 0.129 |  | 0.533 |  | 7.64E-05 |  | 0.389 |  | 4.55E-30 |  | -2.971 |  | 2.07E-39 |
| cg03032214 |  | 18 |  | 74961809 |  | GALR1 |  | 0.734 |  | 0.202 |  | 0.532 |  | 3.99E-05 |  | 0.415 |  | 2.65E-28 |  | -3.710 |  | 1.01E-57 |
| cg18143243 |  | 3 |  | 54155663 |  | CACNA2D3 |  | 0.634 |  | 0.102 |  | 0.532 |  | 9.27E-04 |  | 0.413 |  | 5.55E-18 |  | -1.282 |  | 2.72E-16 |
| cg23564700 |  | 18 |  | 5544169 |  | EPB41L3 |  | 0.643 |  | 0.111 |  | 0.532 |  | 2.52E-04 |  | 0.371 |  | 1.53E-12 |  | -2.714 |  | 6.49E-79 |
| cg12510981 |  | 20 |  | 37434260 |  | PPP1R16B |  | 0.559 |  | 0.028 |  | 0.532 |  | 7.38E-05 |  | 0.453 |  | 1.05E-27 |  | -1.764 |  | 2.82E-42 |
| cg24305584 |  | 10 |  | 91295045 |  | SLC16A12 |  | 0.648 |  | 0.116 |  | 0.531 |  | 1.56E-04 |  | 0.391 |  | 1.62E-22 |  | -2.811 |  | 6.10E-71 |
| cg06172475 |  | 11 |  | 128563903 |  | FLI1 |  | 0.603 |  | 0.072 |  | 0.531 |  | 5.57E-04 |  | 0.440 |  | 1.32E-29 |  | -1.298 |  | 1.09E-29 |
| cg22478591 |  | 4 |  | 176923544 |  | GPM6A |  | 0.561 |  | 0.030 |  | 0.531 |  | 6.23E-05 |  | 0.379 |  | 1.39E-26 |  | -4.531 |  | 2.55E-79 |
| cg00355281 |  | 7 |  | 24323767 |  | NPY |  | 0.727 |  | 0.197 |  | 0.531 |  | 3.19E-05 |  | 0.354 |  | 8.24E-45 |  | -2.903 |  | 1.18E-30 |
| cg13890706 |  | 10 |  | 118033115 |  | GFRA1 |  | 0.683 |  | 0.153 |  | 0.531 |  | 1.50E-04 |  | 0.448 |  | 2.19E-38 |  | -3.175 |  | 2.49E-85 |
| cg04779631 |  | 15 |  | 84115895 |  | SH3GL3 |  | 0.654 |  | 0.124 |  | 0.530 |  | 3.46E-04 |  | 0.398 |  | 1.68E-19 |  | -2.120 |  | 4.81E-11 |
| cg12391690 |  | 1 |  | 38100811 |  | RSPO1 |  | 0.684 |  | 0.155 |  | 0.529 |  | 1.38E-04 |  | 0.392 |  | 1.76E-18 |  | -2.063 |  | 2.33E-23 |
| cg04638468 |  | 4 |  | 122686453 |  | TMEM155 |  | 0.800 |  | 0.271 |  | 0.529 |  | 7.97E-05 |  | 0.475 |  | 5.60E-48 |  | -2.293 |  | 8.56E-42 |
| cg25729826 |  | 3 |  | 121903095 |  | CASR |  | 0.715 |  | 0.187 |  | 0.529 |  | 2.00E-04 |  | 0.510 |  | 2.28E-36 |  | -3.375 |  | 1.14E-61 |
| cg24663256 |  | 4 |  | 21950307 |  | KCNIP4 |  | 0.694 |  | 0.166 |  | 0.528 |  | 4.48E-05 |  | 0.401 |  | 2.61E-26 |  | -2.411 |  | 3.25E-90 |
| cg11172693 |  | 20 |  | 37434229 |  | PPP1R16B |  | 0.695 |  | 0.166 |  | 0.528 |  | 1.19E-04 |  | 0.465 |  | 6.63E-43 |  | -1.764 |  | 2.82E-42 |
| cg09893588 |  | 20 |  | 61340109 |  | NTSR1 |  | 0.616 |  | 0.088 |  | 0.528 |  | 1.36E-03 |  | 0.410 |  | 3.23E-14 |  | -1.391 |  | 3.72E-06 |
| cg04942472 |  | 16 |  | 58497239 |  | NDRG4 |  | 0.630 |  | 0.104 |  | 0.527 |  | 4.22E-05 |  | 0.413 |  | 6.64E-21 |  | -1.187 |  | 2.35E-22 |
| cg21672843 |  | 20 |  | 41818905 |  | PTPRT |  | 0.660 |  | 0.133 |  | 0.527 |  | 3.05E-05 |  | 0.464 |  | 4.05E-40 |  | -1.704 |  | 3.19E-16 |
| cg10273340 |  | 16 |  | 56224793 |  | GNAO1 |  | 0.610 |  | 0.084 |  | 0.526 |  | 9.56E-04 |  | 0.448 |  | 1.97E-22 |  | -3.588 |  | 3.11E-121 |
| cg04442576 |  | 6 |  | 118228650 |  | SLC35F1 |  | 0.637 |  | 0.111 |  | 0.526 |  | 1.86E-04 |  | 0.481 |  | 4.26E-24 |  | -2.178 |  | 8.65E-52 |
| cg04457196 |  | 2 |  | 164593264 |  | FIGN |  | 0.699 |  | 0.173 |  | 0.526 |  | 2.76E-04 |  | 0.464 |  | 1.78E-33 |  | -1.773 |  | 5.16E-29 |
| cg13498858 |  | 19 |  | 44952618 |  | ZNF229 |  | 0.600 |  | 0.074 |  | 0.525 |  | 5.96E-05 |  | 0.398 |  | 5.81E-24 |  | -2.137 |  | 6.37E-44 |
| cg00581482 |  | 1 |  | 154298344 |  | ATP8B2 |  | 0.647 |  | 0.121 |  | 0.525 |  | 6.56E-04 |  | 0.511 |  | 4.24E-34 |  | -1.075 |  | 9.49E-17 |
| cg06392169 |  | 6 |  | 391936 |  | IRF4 |  | 0.711 |  | 0.186 |  | 0.525 |  | 3.93E-05 |  | 0.409 |  | 1.32E-64 |  | -2.419 |  | 2.40E-49 |
| cg22960186 |  | 5 |  | 178017260 |  | COL23A1 |  | 0.570 |  | 0.045 |  | 0.525 |  | 2.19E-04 |  | 0.355 |  | 4.70E-17 |  | -1.089 |  | 4.67E-14 |
| cg06340552 |  | 4 |  | 142054329 |  | RNF150 |  | 0.606 |  | 0.082 |  | 0.525 |  | 1.94E-04 |  | 0.436 |  | 2.61E-21 |  | -3.394 |  | 6.25E-98 |
| cg15542741 |  | 12 |  | 85305677 |  | SLC6A15 |  | 0.667 |  | 0.142 |  | 0.525 |  | 1.93E-04 |  | 0.430 |  | 1.90E-29 |  | -1.371 |  | 1.55E-04 |
| cg19939997 |  | 8 |  | 97173335 |  | GDF6 |  | 0.601 |  | 0.077 |  | 0.524 |  | 4.41E-05 |  | 0.419 |  | 1.10E-37 |  | -1.283 |  | 9.70E-08 |
| cg05311412 |  | 2 |  | 108602906 |  | SLC5A7 |  | 0.708 |  | 0.184 |  | 0.524 |  | 1.25E-04 |  | 0.430 |  | 2.88E-27 |  | -4.278 |  | 1.41E-70 |
| cg24403845 |  | 10 |  | 108924366 |  | SORCS1 |  | 0.845 |  | 0.321 |  | 0.524 |  | 4.60E-05 |  | 0.566 |  | 6.49E-40 |  | -3.835 |  | 2.70E-84 |
| cg07304692 |  | 2 |  | 100939045 |  | LONRF2 |  | 0.615 |  | 0.091 |  | 0.523 |  | 1.90E-05 |  | 0.435 |  | 4.68E-56 |  | -3.768 |  | 7.89E-60 |
| cg02629281 |  | 11 |  | 8102450 |  | TUB |  | 0.588 |  | 0.066 |  | 0.523 |  | 1.56E-04 |  | 0.373 |  | 3.15E-18 |  | -1.937 |  | 1.92E-34 |
| cg21653184 |  | 19 |  | 58609361 |  | ZSCAN18 |  | 0.630 |  | 0.108 |  | 0.522 |  | 1.40E-03 |  | 0.433 |  | 2.47E-16 |  | -1.394 |  | 5.72E-18 |
| cg03538436 |  | 12 |  | 117799370 |  | NOS1 |  | 0.738 |  | 0.215 |  | 0.522 |  | 6.13E-05 |  | 0.431 |  | 1.72E-39 |  | -3.377 |  | 5.07E-46 |
| cg08972130 |  | 12 |  | 24715478 |  | SOX5 |  | 0.561 |  | 0.039 |  | 0.522 |  | 1.53E-03 |  | 0.424 |  | 6.75E-14 |  | -1.403 |  | 1.26E-17 |
| cg09896900 |  | 19 |  | 44952808 |  | ZNF229 |  | 0.768 |  | 0.246 |  | 0.522 |  | 1.14E-04 |  | 0.483 |  | 2.91E-33 |  | -2.137 |  | 6.37E-44 |
| cg02485200 |  | 21 |  | 27011788 |  | JAM2 |  | 0.576 |  | 0.055 |  | 0.522 |  | 4.25E-05 |  | 0.408 |  | 1.10E-17 |  | -2.547 |  | 1.20E-92 |
| cg18237607 |  | 5 |  | 37840075 |  | GDNF |  | 0.663 |  | 0.142 |  | 0.521 |  | 2.30E-04 |  | 0.444 |  | 4.34E-33 |  | -2.297 |  | 8.35E-67 |
| cg12035092 |  | 2 |  | 149633226 |  | KIF5C |  | 0.575 |  | 0.054 |  | 0.521 |  | 1.64E-04 |  | 0.407 |  | 8.60E-17 |  | -2.093 |  | 2.02E-35 |
| cg26532358 |  | 6 |  | 118228871 |  | SLC35F1 |  | 0.598 |  | 0.078 |  | 0.521 |  | 2.11E-04 |  | 0.415 |  | 5.81E-24 |  | -2.178 |  | 8.65E-52 |
| cg23148701 |  | 12 |  | 64062498 |  | DPY19L2 |  | 0.601 |  | 0.080 |  | 0.521 |  | 2.56E-05 |  | 0.417 |  | 1.57E-46 |  | -1.277 |  | 3.73E-10 |
| cg08768569 |  | 10 |  | 106400565 |  | SORCS3 |  | 0.637 |  | 0.118 |  | 0.519 |  | 1.05E-03 |  | 0.414 |  | 2.75E-20 |  | -2.764 |  | 6.34E-24 |
| cg24500900 |  | 20 |  | 61051423 |  | GATA5 |  | 0.698 |  | 0.180 |  | 0.518 |  | 9.13E-05 |  | 0.458 |  | 8.43E-24 |  | -2.304 |  | 5.49E-13 |
| cg22014661 |  | 6 |  | 62996130 |  | KHDRBS2 |  | 0.789 |  | 0.272 |  | 0.518 |  | 7.66E-05 |  | 0.518 |  | 1.71E-48 |  | -4.225 |  | 2.32E-91 |
| cg05212802 |  | 4 |  | 142053623 |  | RNF150 |  | 0.662 |  | 0.144 |  | 0.517 |  | 4.68E-05 |  | 0.356 |  | 4.79E-32 |  | -3.394 |  | 6.25E-98 |
| cg00596508 |  | 12 |  | 41086800 |  | CNTN1 |  | 0.648 |  | 0.131 |  | 0.517 |  | 4.90E-05 |  | 0.355 |  | 6.06E-45 |  | -2.827 |  | 4.42E-36 |
| cg17398252 |  | 6 |  | 105585179 |  | BVES |  | 0.604 |  | 0.086 |  | 0.517 |  | 5.84E-04 |  | 0.425 |  | 2.81E-21 |  | -2.897 |  | 7.75E-98 |
| cg25012919 |  | 13 |  | 36705606 |  | DCLK1 |  | 0.683 |  | 0.166 |  | 0.517 |  | 5.73E-05 |  | 0.498 |  | 1.35E-34 |  | -2.886 |  | 5.36E-68 |
| cg20253551 |  | 6 |  | 152129400 |  | ESR1 |  | 0.675 |  | 0.158 |  | 0.517 |  | 7.06E-05 |  | 0.374 |  | 1.48E-36 |  | -1.207 |  | 2.22E-11 |
| cg11410023 |  | 3 |  | 96533511 |  | EPHA6 |  | 0.762 |  | 0.246 |  | 0.517 |  | 1.10E-04 |  | 0.541 |  | 4.63E-37 |  | -4.332 |  | 2.42E-78 |
| cg24242823 |  | 7 |  | 24323675 |  | NPY |  | 0.650 |  | 0.134 |  | 0.516 |  | 8.74E-05 |  | 0.428 |  | 1.24E-39 |  | -2.903 |  | 1.18E-30 |
| cg05730365 |  | 5 |  | 37838563 |  | GDNF |  | 0.643 |  | 0.128 |  | 0.515 |  | 4.64E-04 |  | 0.397 |  | 1.16E-25 |  | -2.297 |  | 8.35E-67 |
| cg25433624 |  | 20 |  | 37435119 |  | PPP1R16B |  | 0.629 |  | 0.114 |  | 0.515 |  | 1.67E-04 |  | 0.414 |  | 4.50E-43 |  | -1.764 |  | 2.82E-42 |
| cg05919360 |  | 1 |  | 57889417 |  | DAB1 |  | 0.739 |  | 0.224 |  | 0.515 |  | 1.42E-04 |  | 0.399 |  | 1.33E-32 |  | -1.050 |  | 9.62E-06 |
| cg13078140 |  | 4 |  | 20255082 |  | SLIT2 |  | 0.768 |  | 0.255 |  | 0.513 |  | 4.43E-05 |  | 0.484 |  | 2.67E-51 |  | -1.978 |  | 3.90E-26 |
| cg27021357 |  | 15 |  | 84115969 |  | SH3GL3 |  | 0.686 |  | 0.174 |  | 0.513 |  | 2.23E-04 |  | 0.427 |  | 2.48E-20 |  | -2.120 |  | 4.81E-11 |
| cg06468908 |  | 4 |  | 110223713 |  | COL25A1 |  | 0.681 |  | 0.169 |  | 0.512 |  | 7.68E-05 |  | 0.360 |  | 3.63E-30 |  | -1.087 |  | 1.63E-05 |
| cg03734437 |  | 8 |  | 97173526 |  | GDF6 |  | 0.621 |  | 0.109 |  | 0.511 |  | 5.80E-05 |  | 0.512 |  | 2.87E-47 |  | -1.283 |  | 9.70E-08 |
| cg10237419 |  | 4 |  | 110224235 |  | COL25A1 |  | 0.616 |  | 0.105 |  | 0.511 |  | 5.45E-05 |  | 0.409 |  | 9.15E-43 |  | -1.087 |  | 1.63E-05 |
| cg00920970 |  | 6 |  | 152129388 |  | ESR1 |  | 0.668 |  | 0.157 |  | 0.511 |  | 4.90E-05 |  | 0.436 |  | 1.21E-37 |  | -1.207 |  | 2.22E-11 |
| cg01466678 |  | 16 |  | 58497395 |  | NDRG4 |  | 0.580 |  | 0.070 |  | 0.511 |  | 3.47E-05 |  | 0.377 |  | 1.10E-27 |  | -1.187 |  | 2.35E-22 |
| cg02523844 |  | 7 |  | 79083507 |  | MAGI2 |  | 0.747 |  | 0.237 |  | 0.510 |  | 4.72E-04 |  | 0.463 |  | 2.36E-37 |  | -1.368 |  | 4.25E-33 |
| cg21656726 |  | 20 |  | 37434262 |  | PPP1R16B |  | 0.551 |  | 0.041 |  | 0.510 |  | 8.10E-05 |  | 0.436 |  | 1.31E-27 |  | -1.764 |  | 2.82E-42 |
| cg07494047 |  | 19 |  | 57862713 |  | ZNF304 |  | 0.540 |  | 0.030 |  | 0.510 |  | 2.55E-04 |  | 0.367 |  | 7.13E-22 |  | -1.412 |  | 1.49E-19 |
| cg21761844 |  | 19 |  | 37407257 |  | ZNF568 |  | 0.577 |  | 0.068 |  | 0.510 |  | 5.90E-04 |  | 0.415 |  | 1.61E-32 |  | -1.326 |  | 1.57E-19 |
| cg19577779 |  | 13 |  | 88326635 |  | SLITRK5 |  | 0.669 |  | 0.160 |  | 0.510 |  | 6.17E-04 |  | 0.459 |  | 3.44E-34 |  | -1.305 |  | 8.07E-06 |
| cg02269161 |  | 11 |  | 7273154 |  | SYT9 |  | 0.700 |  | 0.191 |  | 0.509 |  | 4.29E-05 |  | 0.356 |  | 2.52E-29 |  | -2.647 |  | 2.49E-31 |
| cg20115218 |  | 11 |  | 22214726 |  | ANO5 |  | 0.597 |  | 0.088 |  | 0.509 |  | 5.47E-04 |  | 0.410 |  | 8.53E-17 |  | -3.178 |  | 6.05E-57 |
| cg21627760 |  | 19 |  | 57862638 |  | ZNF304 |  | 0.553 |  | 0.045 |  | 0.508 |  | 1.59E-04 |  | 0.411 |  | 1.24E-24 |  | -1.412 |  | 1.49E-19 |
| cg09201151 |  | 15 |  | 84116151 |  | SH3GL3 |  | 0.616 |  | 0.109 |  | 0.507 |  | 4.91E-04 |  | 0.424 |  | 1.24E-19 |  | -2.120 |  | 4.81E-11 |
| cg00095976 |  | 6 |  | 118228060 |  | SLC35F1 |  | 0.562 |  | 0.055 |  | 0.507 |  | 1.35E-03 |  | 0.411 |  | 3.50E-16 |  | -2.178 |  | 8.65E-52 |
| cg10756099 |  | 4 |  | 110224036 |  | COL25A1 |  | 0.572 |  | 0.066 |  | 0.507 |  | 3.40E-04 |  | 0.367 |  | 3.49E-26 |  | -1.087 |  | 1.63E-05 |
| cg24834740 |  | 20 |  | 37434552 |  | PPP1R16B |  | 0.596 |  | 0.090 |  | 0.506 |  | 3.92E-05 |  | 0.368 |  | 1.78E-30 |  | -1.764 |  | 2.82E-42 |
| cg14510812 |  | 12 |  | 85306797 |  | SLC6A15 |  | 0.691 |  | 0.185 |  | 0.506 |  | 7.85E-05 |  | 0.488 |  | 5.00E-42 |  | -1.371 |  | 1.55E-04 |
| cg15621322 |  | 21 |  | 28218789 |  | ADAMTS1 |  | 0.568 |  | 0.062 |  | 0.505 |  | 1.70E-03 |  | 0.362 |  | 1.08E-12 |  | -2.043 |  | 2.50E-58 |
| cg09073398 |  | 5 |  | 168727762 |  | SLIT3 |  | 0.775 |  | 0.270 |  | 0.505 |  | 5.90E-05 |  | 0.472 |  | 1.89E-53 |  | -2.537 |  | 2.57E-65 |
| cg14338345 |  | 9 |  | 22447679 |  | DMRTA1 |  | 0.807 |  | 0.301 |  | 0.505 |  | 4.41E-04 |  | 0.437 |  | 1.03E-31 |  | -3.425 |  | 2.36E-68 |
| cg24687051 |  | 6 |  | 73332073 |  | KCNQ5 |  | 0.862 |  | 0.357 |  | 0.505 |  | 3.71E-05 |  | 0.418 |  | 2.99E-55 |  | -2.055 |  | 1.10E-17 |
| cg06650115 |  | 16 |  | 58498585 |  | NDRG4 |  | 0.544 |  | 0.039 |  | 0.505 |  | 5.18E-04 |  | 0.435 |  | 1.71E-21 |  | -1.187 |  | 2.35E-22 |
| cg24239329 |  | 13 |  | 36705622 |  | DCLK1 |  | 0.676 |  | 0.172 |  | 0.504 |  | 8.28E-05 |  | 0.480 |  | 4.89E-37 |  | -2.886 |  | 5.36E-68 |
| cg12486537 |  | 10 |  | 93393030 |  | PPP1R3C |  | 0.616 |  | 0.111 |  | 0.504 |  | 2.72E-03 |  | 0.416 |  | 6.94E-16 |  | -2.387 |  | 9.67E-50 |
| cg21145136 |  | 15 |  | 84116115 |  | SH3GL3 |  | 0.646 |  | 0.142 |  | 0.504 |  | 7.63E-05 |  | 0.407 |  | 2.38E-21 |  | -2.120 |  | 4.81E-11 |
| cg07136998 |  | 5 |  | 168728081 |  | SLIT3 |  | 0.812 |  | 0.309 |  | 0.503 |  | 4.60E-05 |  | 0.572 |  | 3.74E-46 |  | -2.537 |  | 2.57E-65 |
| cg09493505 |  | 7 |  | 49813111 |  | VWC2 |  | 0.629 |  | 0.125 |  | 0.503 |  | 2.14E-03 |  | 0.490 |  | 1.85E-26 |  | -2.201 |  | 5.96E-27 |
| cg00472814 |  | 21 |  | 28217676 |  | ADAMTS1 |  | 0.633 |  | 0.130 |  | 0.503 |  | 4.67E-04 |  | 0.386 |  | 3.63E-18 |  | -2.043 |  | 2.50E-58 |
| cg22403344 |  | 2 |  | 95691500 |  | MAL |  | 0.641 |  | 0.138 |  | 0.503 |  | 3.83E-05 |  | 0.427 |  | 6.17E-52 |  | -3.665 |  | 1.33E-128 |
| cg10486998 |  | 18 |  | 74961787 |  | GALR1 |  | 0.732 |  | 0.229 |  | 0.503 |  | 8.05E-05 |  | 0.418 |  | 5.65E-26 |  | -3.710 |  | 1.01E-57 |
| cg14765646 |  | 2 |  | 154334450 |  | RPRM |  | 0.631 |  | 0.128 |  | 0.503 |  | 1.62E-03 |  | 0.400 |  | 6.88E-14 |  | -2.319 |  | 7.15E-24 |
| cg24262066 |  | 21 |  | 28217060 |  | ADAMTS1 |  | 0.667 |  | 0.165 |  | 0.502 |  | 9.16E-05 |  | 0.403 |  | 4.08E-23 |  | -2.043 |  | 2.50E-58 |
| cg07663789 |  | 5 |  | 32711429 |  | NPR3 |  | 0.568 |  | 0.066 |  | 0.502 |  | 1.55E-03 |  | 0.451 |  | 1.97E-19 |  | -1.195 |  | 2.31E-06 |
| cg26612849 |  | 19 |  | 37407413 |  | ZNF829 |  | 0.549 |  | 0.048 |  | 0.501 |  | 5.33E-04 |  | 0.358 |  | 5.01E-24 |  | -1.193 |  | 1.72E-12 |
| cg02441747 |  | 4 |  | 110223646 |  | COL25A1 |  | 0.721 |  | 0.220 |  | 0.501 |  | 9.23E-05 |  | 0.431 |  | 2.66E-48 |  | -1.087 |  | 1.63E-05 |
| cg02588107 |  | 1 |  | 207669716 |  | CR1 |  | 0.530 |  | 0.029 |  | 0.501 |  | 2.02E-03 |  | 0.456 |  | 1.83E-31 |  | -1.656 |  | 2.67E-16 |
| cg06652199 |  | 12 |  | 5018715 |  | KCNA1 |  | 0.596 |  | 0.095 |  | 0.500 |  | 1.32E-04 |  | 0.401 |  | 3.92E-31 |  | -3.896 |  | 4.28E-50 |
| cg11117364 |  | 11 |  | 94501718 |  | AMOTL1 |  | 0.632 |  | 0.132 |  | 0.500 |  | 1.37E-03 |  | 0.405 |  | 6.96E-14 |  | -1.993 |  | 5.06E-44 |
| cg10731073 |  | 4 |  | 126237373 |  | FAT4 |  | 0.661 |  | 0.161 |  | 0.500 |  | 1.25E-03 |  | 0.402 |  | 4.57E-23 |  | -1.136 |  | 2.29E-13 |
| cg22434409 |  | 4 |  | 21950722 |  | KCNIP4 |  | 0.772 |  | 0.272 |  | 0.500 |  | 5.38E-04 |  | 0.499 |  | 4.36E-30 |  | -2.411 |  | 3.25E-90 |
| cg11108676 |  | 15 |  | 88801004 |  | NTRK3 |  | 0.574 |  | 0.074 |  | 0.500 |  | 5.58E-04 |  | 0.410 |  | 1.05E-22 |  | -2.086 |  | 3.74E-20 |
| cg26945996 |  | 11 |  | 7272876 |  | SYT9 |  | 0.737 |  | 0.240 |  | 0.498 |  | 1.23E-04 |  | 0.463 |  | 5.88E-30 |  | -2.647 |  | 2.49E-31 |
| cg05874561 |  | 4 |  | 154709828 |  | SFRP2 |  | 0.608 |  | 0.111 |  | 0.497 |  | 2.22E-03 |  | 0.425 |  | 2.35E-25 |  | -1.139 |  | 6.63E-05 |
| cg17477445 |  | 12 |  | 64062526 |  | DPY19L2 |  | 0.608 |  | 0.112 |  | 0.496 |  | 2.74E-05 |  | 0.433 |  | 1.24E-41 |  | -1.277 |  | 3.73E-10 |
| cg19156875 |  | 17 |  | 43974446 |  | MAPT |  | 0.658 |  | 0.163 |  | 0.496 |  | 1.04E-04 |  | 0.382 |  | 9.99E-40 |  | -2.373 |  | 2.23E-36 |
| cg24319381 |  | 15 |  | 83316911 |  | CPEB1 |  | 0.684 |  | 0.189 |  | 0.495 |  | 1.14E-04 |  | 0.413 |  | 3.88E-33 |  | -3.325 |  | 4.24E-93 |
| cg17305266 |  | 6 |  | 1391265 |  | FOXF2 |  | 0.673 |  | 0.178 |  | 0.495 |  | 1.48E-04 |  | 0.458 |  | 2.10E-28 |  | -2.197 |  | 1.79E-66 |
| cg14613271 |  | 2 |  | 108602931 |  | SLC5A7 |  | 0.719 |  | 0.224 |  | 0.495 |  | 2.35E-04 |  | 0.440 |  | 6.97E-28 |  | -4.278 |  | 1.41E-70 |
| cg16437728 |  | 11 |  | 7273046 |  | SYT9 |  | 0.736 |  | 0.241 |  | 0.495 |  | 8.18E-05 |  | 0.495 |  | 1.07E-29 |  | -2.647 |  | 2.49E-31 |
| cg16697214 |  | 7 |  | 50343361 |  | IKZF1 |  | 0.670 |  | 0.175 |  | 0.495 |  | 4.41E-05 |  | 0.426 |  | 7.18E-36 |  | -1.553 |  | 4.62E-27 |
| cg03561455 |  | 18 |  | 70210433 |  | CBLN2 |  | 0.643 |  | 0.150 |  | 0.493 |  | 1.13E-03 |  | 0.465 |  | 1.99E-28 |  | -2.950 |  | 6.25E-75 |
| cg11832210 |  | 10 |  | 91295346 |  | SLC16A12 |  | 0.667 |  | 0.175 |  | 0.493 |  | 2.23E-04 |  | 0.418 |  | 8.46E-25 |  | -2.811 |  | 6.10E-71 |
| cg14042851 |  | 19 |  | 57019005 |  | ZNF471 |  | 0.703 |  | 0.212 |  | 0.492 |  | 2.55E-04 |  | 0.416 |  | 4.13E-24 |  | -1.718 |  | 1.59E-22 |
| cg04555373 |  | 8 |  | 31497042 |  | NRG1 |  | 0.714 |  | 0.222 |  | 0.492 |  | 8.82E-05 |  | 0.464 |  | 1.49E-39 |  | -1.447 |  | 5.33E-16 |
| cg18771173 |  | 2 |  | 108602937 |  | SLC5A7 |  | 0.767 |  | 0.276 |  | 0.491 |  | 1.97E-04 |  | 0.423 |  | 2.00E-28 |  | -4.278 |  | 1.41E-70 |
| cg23898073 |  | 10 |  | 118032948 |  | GFRA1 |  | 0.563 |  | 0.072 |  | 0.491 |  | 6.89E-05 |  | 0.391 |  | 1.26E-31 |  | -3.175 |  | 2.49E-85 |
| cg04184836 |  | 15 |  | 83316640 |  | CPEB1 |  | 0.605 |  | 0.114 |  | 0.491 |  | 1.42E-04 |  | 0.499 |  | 4.37E-32 |  | -3.325 |  | 4.24E-93 |
| cg24039631 |  | 19 |  | 56905013 |  | ZNF582 |  | 0.684 |  | 0.194 |  | 0.491 |  | 6.20E-05 |  | 0.403 |  | 3.41E-28 |  | -1.345 |  | 5.51E-20 |
| cg25088758 |  | 4 |  | 110223598 |  | COL25A1 |  | 0.674 |  | 0.183 |  | 0.490 |  | 6.77E-05 |  | 0.459 |  | 1.45E-54 |  | -1.087 |  | 1.63E-05 |
| cg08684639 |  | 4 |  | 176987313 |  | WDR17 |  | 0.526 |  | 0.035 |  | 0.490 |  | 1.87E-03 |  | 0.464 |  | 1.30E-25 |  | -2.723 |  | 4.08E-37 |
| cg07003632 |  | 14 |  | 100259329 |  | EML1 |  | 0.574 |  | 0.084 |  | 0.490 |  | 1.87E-04 |  | 0.390 |  | 1.11E-20 |  | -2.393 |  | 3.13E-87 |
| cg19788741 |  | 5 |  | 101632310 |  | SLCO4C1 |  | 0.526 |  | 0.036 |  | 0.490 |  | 3.47E-04 |  | 0.355 |  | 1.77E-10 |  | -3.544 |  | 3.29E-76 |
| cg09301294 |  | 3 |  | 96533520 |  | EPHA6 |  | 0.661 |  | 0.171 |  | 0.489 |  | 9.54E-05 |  | 0.420 |  | 1.36E-28 |  | -4.332 |  | 2.42E-78 |
| cg01151966 |  | 10 |  | 83634485 |  | NRG3 |  | 0.721 |  | 0.232 |  | 0.489 |  | 5.29E-05 |  | 0.418 |  | 3.87E-42 |  | -2.071 |  | 2.56E-25 |
| cg03723506 |  | 5 |  | 38557143 |  | LIFR |  | 0.610 |  | 0.121 |  | 0.489 |  | 4.43E-04 |  | 0.420 |  | 1.98E-26 |  | -3.382 |  | 6.98E-134 |
| cg24021956 |  | 7 |  | 79083509 |  | MAGI2 |  | 0.752 |  | 0.263 |  | 0.488 |  | 4.90E-04 |  | 0.447 |  | 1.89E-39 |  | -1.368 |  | 4.25E-33 |
| cg08516516 |  | 5 |  | 115152492 |  | CDO1 |  | 0.681 |  | 0.193 |  | 0.488 |  | 7.86E-05 |  | 0.381 |  | 2.60E-42 |  | -1.956 |  | 2.66E-22 |
| cg09258813 |  | 8 |  | 37823409 |  | ADRB3 |  | 0.664 |  | 0.176 |  | 0.488 |  | 4.04E-05 |  | 0.486 |  | 1.27E-29 |  | -2.523 |  | 2.53E-24 |
| cg02660347 |  | 12 |  | 64062505 |  | DPY19L2 |  | 0.762 |  | 0.274 |  | 0.488 |  | 4.76E-05 |  | 0.379 |  | 1.48E-37 |  | -1.277 |  | 3.73E-10 |
| cg04991805 |  | 10 |  | 99790910 |  | CRTAC1 |  | 0.790 |  | 0.302 |  | 0.488 |  | 3.08E-05 |  | 0.424 |  | 4.92E-45 |  | -2.051 |  | 1.49E-17 |
| cg04454951 |  | 7 |  | 49813031 |  | VWC2 |  | 0.660 |  | 0.173 |  | 0.487 |  | 7.30E-04 |  | 0.461 |  | 1.73E-26 |  | -2.201 |  | 5.96E-27 |
| cg02918577 |  | 15 |  | 93633145 |  | RGMA |  | 0.659 |  | 0.172 |  | 0.487 |  | 3.55E-04 |  | 0.418 |  | 5.02E-21 |  | -2.430 |  | 1.53E-49 |
| cg00737840 |  | 11 |  | 125036420 |  | PKNOX2 |  | 0.663 |  | 0.176 |  | 0.487 |  | 1.32E-04 |  | 0.373 |  | 3.49E-30 |  | -2.749 |  | 1.20E-86 |
| cg00687686 |  | 16 |  | 58497236 |  | NDRG4 |  | 0.569 |  | 0.083 |  | 0.487 |  | 2.53E-04 |  | 0.395 |  | 5.10E-19 |  | -1.187 |  | 2.35E-22 |
| cg03700449 |  | 12 |  | 103352326 |  | ASCL1 |  | 0.641 |  | 0.154 |  | 0.486 |  | 2.00E-05 |  | 0.367 |  | 6.99E-25 |  | -1.622 |  | 6.21E-08 |
| cg14553600 |  | 21 |  | 27011793 |  | JAM2 |  | 0.570 |  | 0.084 |  | 0.486 |  | 4.75E-05 |  | 0.395 |  | 1.02E-22 |  | -2.547 |  | 1.20E-92 |
| cg22676693 |  | 1 |  | 57888581 |  | DAB1 |  | 0.588 |  | 0.102 |  | 0.486 |  | 9.61E-04 |  | 0.359 |  | 2.56E-19 |  | -1.050 |  | 9.62E-06 |
| cg10390058 |  | 18 |  | 74962794 |  | GALR1 |  | 0.773 |  | 0.287 |  | 0.486 |  | 3.40E-05 |  | 0.389 |  | 3.17E-41 |  | -3.710 |  | 1.01E-57 |
| cg03430846 |  | 8 |  | 31497082 |  | NRG1 |  | 0.837 |  | 0.352 |  | 0.486 |  | 4.54E-05 |  | 0.470 |  | 2.35E-41 |  | -1.447 |  | 5.33E-16 |
| cg19320476 |  | 11 |  | 82443592 |  | FAM181B |  | 0.671 |  | 0.186 |  | 0.486 |  | 4.22E-04 |  | 0.379 |  | 5.20E-15 |  | -2.134 |  | 2.81E-19 |
| cg01539036 |  | 20 |  | 61340116 |  | NTSR1 |  | 0.572 |  | 0.086 |  | 0.486 |  | 1.31E-03 |  | 0.386 |  | 1.91E-15 |  | -1.391 |  | 3.72E-06 |
| cg02732804 |  | 16 |  | 28075269 |  | GSG1L |  | 0.563 |  | 0.078 |  | 0.486 |  | 5.99E-04 |  | 0.505 |  | 4.42E-29 |  | -2.813 |  | 1.91E-35 |
| cg24307799 |  | 3 |  | 238931 |  | CHL1 |  | 0.641 |  | 0.156 |  | 0.486 |  | 4.32E-05 |  | 0.370 |  | 3.53E-31 |  | -2.660 |  | 2.33E-61 |
| cg03698948 |  | 6 |  | 39760092 |  | DAAM2 |  | 0.637 |  | 0.151 |  | 0.485 |  | 5.24E-05 |  | 0.372 |  | 3.04E-18 |  | -1.924 |  | 4.59E-65 |
| cg25842285 |  | 10 |  | 101089972 |  | CNNM1 |  | 0.727 |  | 0.242 |  | 0.485 |  | 4.47E-05 |  | 0.497 |  | 3.17E-54 |  | -1.045 |  | 2.68E-06 |
| cg27034576 |  | 10 |  | 118031654 |  | GFRA1 |  | 0.578 |  | 0.093 |  | 0.485 |  | 5.02E-04 |  | 0.438 |  | 3.70E-43 |  | -3.175 |  | 2.49E-85 |
| cg18011364 |  | 7 |  | 103630086 |  | RELN |  | 0.577 |  | 0.092 |  | 0.485 |  | 5.44E-05 |  | 0.403 |  | 8.18E-27 |  | -2.738 |  | 1.53E-45 |
| cg06617456 |  | 8 |  | 68864769 |  | PREX2 |  | 0.724 |  | 0.239 |  | 0.485 |  | 5.18E-05 |  | 0.470 |  | 2.07E-67 |  | -1.384 |  | 1.68E-22 |
| cg10074544 |  | 4 |  | 110223700 |  | COL25A1 |  | 0.639 |  | 0.155 |  | 0.485 |  | 9.52E-05 |  | 0.384 |  | 3.47E-41 |  | -1.087 |  | 1.63E-05 |
| cg07167168 |  | 20 |  | 41818788 |  | PTPRT |  | 0.674 |  | 0.190 |  | 0.484 |  | 5.18E-05 |  | 0.447 |  | 3.60E-40 |  | -1.704 |  | 3.19E-16 |
| cg12742937 |  | 2 |  | 40678618 |  | SLC8A1 |  | 0.846 |  | 0.362 |  | 0.484 |  | 8.48E-06 |  | 0.394 |  | 4.29E-33 |  | -1.676 |  | 1.70E-42 |
| cg08154348 |  | 6 |  | 84562930 |  | RIPPLY2 |  | 0.532 |  | 0.048 |  | 0.484 |  | 2.77E-04 |  | 0.358 |  | 7.89E-19 |  | -1.130 |  | 7.10E-03 |
| cg16714055 |  | 20 |  | 61051341 |  | GATA5 |  | 0.602 |  | 0.118 |  | 0.484 |  | 4.68E-04 |  | 0.504 |  | 5.26E-24 |  | -2.304 |  | 5.49E-13 |
| cg07352438 |  | 18 |  | 5543548 |  | EPB41L3 |  | 0.754 |  | 0.271 |  | 0.483 |  | 7.09E-04 |  | 0.435 |  | 4.96E-14 |  | -2.714 |  | 6.49E-79 |
| cg03217795 |  | 16 |  | 23847556 |  | PRKCB |  | 0.513 |  | 0.030 |  | 0.483 |  | 3.45E-04 |  | 0.409 |  | 2.42E-23 |  | -2.494 |  | 2.13E-73 |
| cg12855522 |  | 6 |  | 36808207 |  | CPNE5 |  | 0.536 |  | 0.053 |  | 0.482 |  | 7.23E-04 |  | 0.440 |  | 1.58E-26 |  | -1.991 |  | 1.08E-63 |
| cg07972135 |  | 11 |  | 105481322 |  | GRIA4 |  | 0.749 |  | 0.266 |  | 0.482 |  | 9.19E-05 |  | 0.485 |  | 2.34E-48 |  | -2.716 |  | 1.42E-52 |
| cg07204550 |  | 4 |  | 21950155 |  | KCNIP4 |  | 0.645 |  | 0.163 |  | 0.482 |  | 7.47E-05 |  | 0.355 |  | 1.05E-27 |  | -2.411 |  | 3.25E-90 |
| cg09092054 |  | 22 |  | 19511987 |  | CLDN5 |  | 0.580 |  | 0.098 |  | 0.481 |  | 8.33E-05 |  | 0.388 |  | 2.46E-23 |  | -1.805 |  | 1.23E-30 |
| cg14330641 |  | 4 |  | 154710399 |  | SFRP2 |  | 0.585 |  | 0.104 |  | 0.481 |  | 9.44E-04 |  | 0.402 |  | 4.85E-23 |  | -1.139 |  | 6.63E-05 |
| cg16727201 |  | 18 |  | 12254556 |  | CIDEA |  | 0.779 |  | 0.297 |  | 0.481 |  | 2.69E-04 |  | 0.406 |  | 1.80E-31 |  | -1.867 |  | 5.19E-05 |
| cg12005098 |  | 10 |  | 91295338 |  | SLC16A12 |  | 0.608 |  | 0.127 |  | 0.481 |  | 2.27E-04 |  | 0.396 |  | 5.12E-23 |  | -2.811 |  | 6.10E-71 |
| cg05774801 |  | 4 |  | 154709756 |  | SFRP2 |  | 0.583 |  | 0.102 |  | 0.481 |  | 1.02E-03 |  | 0.358 |  | 1.42E-23 |  | -1.139 |  | 6.63E-05 |
| cg00910695 |  | 8 |  | 109095568 |  | RSPO2 |  | 0.624 |  | 0.144 |  | 0.480 |  | 1.27E-04 |  | 0.509 |  | 4.93E-50 |  | -4.155 |  | 1.48E-92 |
| cg19852958 |  | 4 |  | 13545760 |  | NKX3-2 |  | 0.783 |  | 0.303 |  | 0.480 |  | 3.25E-05 |  | 0.499 |  | 1.08E-38 |  | -1.918 |  | 1.44E-32 |
| cg22821324 |  | 4 |  | 176923439 |  | GPM6A |  | 0.633 |  | 0.153 |  | 0.480 |  | 5.85E-04 |  | 0.475 |  | 1.04E-46 |  | -4.531 |  | 2.55E-79 |
| cg02500300 |  | 4 |  | 184827754 |  | STOX2 |  | 0.618 |  | 0.139 |  | 0.479 |  | 2.62E-03 |  | 0.519 |  | 5.35E-46 |  | -2.513 |  | 3.34E-75 |
| cg11732619 |  | 5 |  | 168728076 |  | SLIT3 |  | 0.640 |  | 0.161 |  | 0.479 |  | 3.12E-05 |  | 0.418 |  | 1.38E-39 |  | -2.537 |  | 2.57E-65 |
| cg11982072 |  | 20 |  | 61051348 |  | GATA5 |  | 0.628 |  | 0.149 |  | 0.479 |  | 4.84E-04 |  | 0.477 |  | 6.58E-22 |  | -2.304 |  | 5.49E-13 |
| cg01645753 |  | 15 |  | 83316734 |  | CPEB1 |  | 0.607 |  | 0.128 |  | 0.479 |  | 7.06E-05 |  | 0.428 |  | 1.28E-33 |  | -3.325 |  | 4.24E-93 |
| cg15322783 |  | 19 |  | 37407152 |  | ZNF829 |  | 0.541 |  | 0.062 |  | 0.479 |  | 4.77E-04 |  | 0.433 |  | 5.40E-28 |  | -1.193 |  | 1.72E-12 |
| cg07155336 |  | 1 |  | 107683775 |  | NTNG1 |  | 0.536 |  | 0.058 |  | 0.478 |  | 6.61E-03 |  | 0.422 |  | 5.78E-13 |  | -2.951 |  | 3.02E-31 |
| cg08576864 |  | 4 |  | 176922779 |  | GPM6A |  | 0.558 |  | 0.080 |  | 0.478 |  | 4.57E-04 |  | 0.469 |  | 5.56E-28 |  | -4.531 |  | 2.55E-79 |
| cg24924779 |  | 20 |  | 49639998 |  | KCNG1 |  | 0.701 |  | 0.223 |  | 0.478 |  | 4.34E-05 |  | 0.423 |  | 1.20E-39 |  | -1.329 |  | 5.33E-08 |
| cg11267955 |  | 5 |  | 101632327 |  | SLCO4C1 |  | 0.508 |  | 0.030 |  | 0.478 |  | 3.32E-04 |  | 0.352 |  | 1.83E-10 |  | -3.544 |  | 3.29E-76 |
| cg04417028 |  | 11 |  | 110583882 |  | ARHGAP20 |  | 0.550 |  | 0.073 |  | 0.477 |  | 2.64E-04 |  | 0.370 |  | 1.52E-24 |  | -2.604 |  | 8.71E-68 |
| cg16136098 |  | 1 |  | 107683532 |  | NTNG1 |  | 0.591 |  | 0.114 |  | 0.477 |  | 6.23E-04 |  | 0.356 |  | 6.11E-21 |  | -2.951 |  | 3.02E-31 |
| cg23811464 |  | 12 |  | 24716204 |  | SOX5 |  | 0.501 |  | 0.024 |  | 0.477 |  | 6.23E-04 |  | 0.375 |  | 1.93E-23 |  | -1.403 |  | 1.26E-17 |
| cg23421023 |  | 8 |  | 40755165 |  | ZMAT4 |  | 0.678 |  | 0.202 |  | 0.476 |  | 9.49E-05 |  | 0.358 |  | 2.72E-29 |  | -3.032 |  | 6.68E-36 |
| cg25167643 |  | 7 |  | 121513538 |  | PTPRZ1 |  | 0.548 |  | 0.072 |  | 0.475 |  | 2.01E-03 |  | 0.375 |  | 2.68E-12 |  | -3.009 |  | 2.53E-32 |
| cg23770904 |  | 20 |  | 61051561 |  | GATA5 |  | 0.663 |  | 0.188 |  | 0.475 |  | 1.42E-04 |  | 0.499 |  | 1.05E-29 |  | -2.304 |  | 5.49E-13 |
| cg08729318 |  | 12 |  | 29936839 |  | TMTC1 |  | 0.784 |  | 0.310 |  | 0.475 |  | 4.63E-05 |  | 0.450 |  | 5.45E-36 |  | -1.822 |  | 3.70E-37 |
| cg27341472 |  | 7 |  | 19812592 |  | TMEM196 |  | 0.725 |  | 0.251 |  | 0.474 |  | 3.66E-05 |  | 0.473 |  | 4.08E-29 |  | -2.732 |  | 4.84E-24 |
| cg02910208 |  | 7 |  | 54610022 |  | VSTM2A |  | 0.606 |  | 0.132 |  | 0.474 |  | 5.88E-05 |  | 0.394 |  | 1.31E-30 |  | -5.178 |  | 1.84E-132 |
| cg02973171 |  | 11 |  | 82443614 |  | FAM181B |  | 0.769 |  | 0.296 |  | 0.474 |  | 5.50E-05 |  | 0.384 |  | 3.62E-16 |  | -2.134 |  | 2.81E-19 |
| cg18277754 |  | 2 |  | 30144152 |  | ALK |  | 0.693 |  | 0.220 |  | 0.474 |  | 8.87E-05 |  | 0.381 |  | 4.99E-43 |  | -2.020 |  | 1.27E-11 |
| cg12804010 |  | 1 |  | 114697113 |  | SYT6 |  | 0.582 |  | 0.108 |  | 0.474 |  | 6.76E-04 |  | 0.432 |  | 4.66E-26 |  | -1.841 |  | 2.36E-13 |
| cg23676439 |  | 4 |  | 155663836 |  | LRAT |  | 0.678 |  | 0.205 |  | 0.474 |  | 9.56E-05 |  | 0.353 |  | 5.29E-37 |  | -3.481 |  | 1.74E-57 |
| cg10573018 |  | 1 |  | 38100837 |  | RSPO1 |  | 0.775 |  | 0.302 |  | 0.473 |  | 2.67E-04 |  | 0.370 |  | 5.54E-21 |  | -2.063 |  | 2.33E-23 |
| cg22889573 |  | 3 |  | 134369939 |  | KY |  | 0.708 |  | 0.235 |  | 0.473 |  | 1.38E-04 |  | 0.470 |  | 2.65E-34 |  | -2.631 |  | 4.72E-53 |
| cg12412079 |  | 3 |  | 68981877 |  | FAM19A4 |  | 0.679 |  | 0.205 |  | 0.473 |  | 8.71E-05 |  | 0.465 |  | 6.67E-36 |  | -2.758 |  | 4.88E-22 |
| cg08553437 |  | 4 |  | 122686456 |  | TMEM155 |  | 0.718 |  | 0.245 |  | 0.473 |  | 8.70E-05 |  | 0.411 |  | 9.25E-45 |  | -2.293 |  | 8.56E-42 |
| cg01664666 |  | 1 |  | 242687465 |  | PLD5 |  | 0.601 |  | 0.128 |  | 0.473 |  | 6.31E-04 |  | 0.394 |  | 1.01E-27 |  | -3.256 |  | 1.14E-39 |
| cg04377145 |  | 6 |  | 73331191 |  | KCNQ5 |  | 0.588 |  | 0.115 |  | 0.473 |  | 5.15E-05 |  | 0.367 |  | 2.03E-46 |  | -2.055 |  | 1.10E-17 |
| cg24366702 |  | 19 |  | 58951778 |  | ZNF132 |  | 0.590 |  | 0.117 |  | 0.473 |  | 8.09E-05 |  | 0.381 |  | 1.20E-39 |  | -1.161 |  | 1.21E-40 |
| cg17393267 |  | 3 |  | 192127356 |  | FGF12 |  | 0.604 |  | 0.132 |  | 0.472 |  | 5.53E-05 |  | 0.380 |  | 5.99E-68 |  | -1.036 |  | 1.80E-10 |
| cg15988350 |  | 7 |  | 32110650 |  | PDE1C |  | 0.592 |  | 0.121 |  | 0.472 |  | 1.61E-04 |  | 0.426 |  | 2.52E-22 |  | -1.555 |  | 8.21E-34 |
| cg01394819 |  | 1 |  | 53527665 |  | PODN |  | 0.533 |  | 0.061 |  | 0.471 |  | 2.93E-03 |  | 0.411 |  | 2.11E-11 |  | -1.062 |  | 2.60E-10 |
| cg10379890 |  | 6 |  | 391208 |  | IRF4 |  | 0.668 |  | 0.197 |  | 0.470 |  | 2.17E-04 |  | 0.360 |  | 3.32E-39 |  | -2.419 |  | 2.40E-49 |
| cg08130988 |  | 2 |  | 56150925 |  | EFEMP1 |  | 0.662 |  | 0.192 |  | 0.470 |  | 1.57E-04 |  | 0.372 |  | 2.50E-28 |  | -1.459 |  | 5.03E-19 |
| cg13566610 |  | 7 |  | 103630083 |  | RELN |  | 0.651 |  | 0.181 |  | 0.469 |  | 5.39E-05 |  | 0.356 |  | 1.74E-32 |  | -2.738 |  | 1.53E-45 |
| cg00845942 |  | 12 |  | 64062724 |  | DPY19L2 |  | 0.747 |  | 0.278 |  | 0.469 |  | 1.45E-04 |  | 0.403 |  | 3.78E-19 |  | -1.277 |  | 3.73E-10 |
| cg12417685 |  | 3 |  | 68981852 |  | FAM19A4 |  | 0.636 |  | 0.167 |  | 0.469 |  | 3.57E-04 |  | 0.448 |  | 2.00E-38 |  | -2.758 |  | 4.88E-22 |
| cg05700339 |  | 18 |  | 12254566 |  | CIDEA |  | 0.681 |  | 0.213 |  | 0.468 |  | 8.12E-05 |  | 0.374 |  | 1.30E-37 |  | -1.867 |  | 5.19E-05 |
| cg10658542 |  | 12 |  | 33592642 |  | SYT10 |  | 0.533 |  | 0.066 |  | 0.466 |  | 3.06E-04 |  | 0.397 |  | 7.30E-18 |  | -3.471 |  | 2.55E-43 |
| cg18404308 |  | 2 |  | 98962900 |  | CNGA3 |  | 0.621 |  | 0.155 |  | 0.466 |  | 4.64E-04 |  | 0.409 |  | 4.78E-32 |  | -2.976 |  | 1.63E-30 |
| cg00314966 |  | 16 |  | 86544346 |  | FOXF1 |  | 0.655 |  | 0.189 |  | 0.466 |  | 4.40E-04 |  | 0.358 |  | 8.90E-18 |  | -1.462 |  | 2.83E-36 |
| cg17151990 |  | 8 |  | 58907242 |  | FAM110B |  | 0.592 |  | 0.127 |  | 0.466 |  | 7.66E-04 |  | 0.412 |  | 1.02E-23 |  | -1.448 |  | 2.40E-23 |
| cg18213472 |  | 6 |  | 84419189 |  | SNAP91 |  | 0.658 |  | 0.193 |  | 0.466 |  | 5.67E-04 |  | 0.460 |  | 1.04E-33 |  | -3.160 |  | 4.28E-42 |
| cg04349243 |  | 10 |  | 91295159 |  | SLC16A12 |  | 0.658 |  | 0.193 |  | 0.465 |  | 2.64E-04 |  | 0.411 |  | 4.04E-25 |  | -2.811 |  | 6.10E-71 |
| cg09868336 |  | 3 |  | 68981866 |  | FAM19A4 |  | 0.752 |  | 0.287 |  | 0.465 |  | 1.90E-04 |  | 0.521 |  | 8.72E-37 |  | -2.758 |  | 4.88E-22 |
| cg14170313 |  | 9 |  | 77112896 |  | RORB |  | 0.617 |  | 0.152 |  | 0.465 |  | 2.08E-03 |  | 0.456 |  | 1.95E-21 |  | -1.632 |  | 3.66E-12 |
| cg27600205 |  | 4 |  | 134070416 |  | PCDH10 |  | 0.713 |  | 0.249 |  | 0.464 |  | 8.23E-05 |  | 0.375 |  | 2.92E-24 |  | -2.971 |  | 2.07E-39 |
| cg15778745 |  | 13 |  | 88324570 |  | SLITRK5 |  | 0.702 |  | 0.238 |  | 0.464 |  | 6.90E-03 |  | 0.511 |  | 6.59E-24 |  | -1.305 |  | 8.07E-06 |
| cg20546002 |  | 5 |  | 178017376 |  | COL23A1 |  | 0.523 |  | 0.059 |  | 0.464 |  | 5.19E-04 |  | 0.360 |  | 4.73E-22 |  | -1.089 |  | 4.67E-14 |
| cg23003534 |  | 10 |  | 105036747 |  | INA |  | 0.542 |  | 0.078 |  | 0.463 |  | 1.94E-04 |  | 0.390 |  | 5.97E-29 |  | -2.726 |  | 9.36E-33 |
| cg17767285 |  | 6 |  | 84419202 |  | SNAP91 |  | 0.588 |  | 0.125 |  | 0.463 |  | 6.08E-04 |  | 0.476 |  | 1.79E-32 |  | -3.160 |  | 4.28E-42 |
| cg17385936 |  | 3 |  | 132756986 |  | TMEM108 |  | 0.829 |  | 0.367 |  | 0.463 |  | 7.02E-05 |  | 0.438 |  | 3.02E-30 |  | -1.931 |  | 2.61E-51 |
| cg24792682 |  | 10 |  | 118031632 |  | GFRA1 |  | 0.580 |  | 0.118 |  | 0.462 |  | 2.26E-04 |  | 0.408 |  | 3.43E-40 |  | -3.175 |  | 2.49E-85 |
| cg02547269 |  | 6 |  | 1390361 |  | FOXF2 |  | 0.625 |  | 0.163 |  | 0.462 |  | 1.08E-03 |  | 0.362 |  | 7.42E-16 |  | -2.197 |  | 1.79E-66 |
| cg12219082 |  | 3 |  | 68981854 |  | FAM19A4 |  | 0.630 |  | 0.168 |  | 0.462 |  | 4.62E-04 |  | 0.440 |  | 6.38E-37 |  | -2.758 |  | 4.88E-22 |
| cg09695033 |  | 2 |  | 29337988 |  | CLIP4 |  | 0.676 |  | 0.215 |  | 0.461 |  | 5.40E-05 |  | 0.365 |  | 4.18E-39 |  | -2.068 |  | 2.57E-41 |
| cg16434547 |  | 6 |  | 152957995 |  | SYNE1 |  | 0.492 |  | 0.031 |  | 0.461 |  | 2.28E-04 |  | 0.354 |  | 5.55E-25 |  | -1.507 |  | 6.57E-25 |
| cg03559235 |  | 2 |  | 100937944 |  | LONRF2 |  | 0.691 |  | 0.230 |  | 0.461 |  | 2.82E-05 |  | 0.401 |  | 1.95E-57 |  | -3.768 |  | 7.89E-60 |
| cg07594636 |  | 2 |  | 68547088 |  | CNRIP1 |  | 0.632 |  | 0.173 |  | 0.459 |  | 2.55E-04 |  | 0.356 |  | 1.56E-31 |  | -1.478 |  | 1.55E-40 |
| cg07994622 |  | 20 |  | 4804052 |  | RASSF2 |  | 0.517 |  | 0.058 |  | 0.459 |  | 1.30E-04 |  | 0.400 |  | 1.80E-15 |  | -1.261 |  | 7.80E-23 |
| cg18221862 |  | 2 |  | 193059230 |  | TMEFF2 |  | 0.569 |  | 0.111 |  | 0.459 |  | 9.26E-05 |  | 0.413 |  | 3.82E-32 |  | -4.134 |  | 3.79E-97 |
| cg20872937 |  | 18 |  | 74961968 |  | GALR1 |  | 0.696 |  | 0.238 |  | 0.458 |  | 4.64E-05 |  | 0.493 |  | 7.61E-33 |  | -3.710 |  | 1.01E-57 |
| cg14625113 |  | 12 |  | 41086680 |  | CNTN1 |  | 0.745 |  | 0.287 |  | 0.458 |  | 5.87E-05 |  | 0.396 |  | 2.78E-44 |  | -2.827 |  | 4.42E-36 |
| cg00868383 |  | 19 |  | 58951814 |  | ZNF132 |  | 0.496 |  | 0.037 |  | 0.458 |  | 5.95E-04 |  | 0.407 |  | 2.55E-36 |  | -1.161 |  | 1.21E-40 |
| cg21657955 |  | 7 |  | 19812578 |  | TMEM196 |  | 0.634 |  | 0.176 |  | 0.458 |  | 6.47E-05 |  | 0.372 |  | 4.68E-27 |  | -2.732 |  | 4.84E-24 |
| cg09303936 |  | 6 |  | 73331290 |  | KCNQ5 |  | 0.611 |  | 0.153 |  | 0.458 |  | 2.23E-04 |  | 0.378 |  | 3.69E-37 |  | -2.055 |  | 1.10E-17 |
| cg04529370 |  | 10 |  | 83635300 |  | NRG3 |  | 0.743 |  | 0.285 |  | 0.458 |  | 7.50E-05 |  | 0.360 |  | 3.94E-50 |  | -2.071 |  | 2.56E-25 |
| cg07283152 |  | 11 |  | 8284997 |  | LMO1 |  | 0.581 |  | 0.124 |  | 0.457 |  | 1.78E-04 |  | 0.355 |  | 3.18E-15 |  | -1.704 |  | 1.50E-07 |
| cg18844382 |  | 14 |  | 23834977 |  | EFS |  | 0.645 |  | 0.188 |  | 0.457 |  | 1.30E-04 |  | 0.482 |  | 8.76E-36 |  | -1.192 |  | 4.46E-19 |
| cg16919517 |  | 20 |  | 61050914 |  | GATA5 |  | 0.603 |  | 0.146 |  | 0.457 |  | 8.11E-04 |  | 0.509 |  | 9.28E-24 |  | -2.304 |  | 5.49E-13 |
| cg20392607 |  | 12 |  | 5018798 |  | KCNA1 |  | 0.548 |  | 0.091 |  | 0.457 |  | 6.37E-04 |  | 0.459 |  | 2.29E-37 |  | -3.896 |  | 4.28E-50 |
| cg25552435 |  | 5 |  | 178017300 |  | COL23A1 |  | 0.614 |  | 0.158 |  | 0.456 |  | 3.08E-04 |  | 0.417 |  | 8.87E-25 |  | -1.089 |  | 4.67E-14 |
| cg15603568 |  | 11 |  | 105481283 |  | GRIA4 |  | 0.791 |  | 0.336 |  | 0.456 |  | 1.96E-04 |  | 0.432 |  | 1.24E-45 |  | -2.716 |  | 1.42E-52 |
| cg04842146 |  | 8 |  | 85097056 |  | RALYL |  | 0.705 |  | 0.250 |  | 0.456 |  | 8.34E-04 |  | 0.520 |  | 2.23E-40 |  | -3.144 |  | 4.93E-29 |
| cg23180938 |  | 5 |  | 115152485 |  | CDO1 |  | 0.643 |  | 0.188 |  | 0.455 |  | 1.64E-04 |  | 0.379 |  | 6.06E-44 |  | -1.956 |  | 2.66E-22 |
| cg20599022 |  | 4 |  | 142054446 |  | RNF150 |  | 0.586 |  | 0.130 |  | 0.455 |  | 4.50E-05 |  | 0.369 |  | 6.15E-20 |  | -3.394 |  | 6.25E-98 |
| cg18206027 |  | 7 |  | 49813486 |  | VWC2 |  | 0.552 |  | 0.097 |  | 0.455 |  | 2.90E-04 |  | 0.450 |  | 3.25E-40 |  | -2.201 |  | 5.96E-27 |
| cg22491927 |  | 19 |  | 13617091 |  | CACNA1A |  | 0.763 |  | 0.308 |  | 0.455 |  | 5.44E-05 |  | 0.449 |  | 1.31E-38 |  | -1.407 |  | 1.61E-21 |
| cg08458292 |  | 1 |  | 57890610 |  | DAB1 |  | 0.724 |  | 0.269 |  | 0.455 |  | 1.65E-03 |  | 0.380 |  | 2.22E-18 |  | -1.050 |  | 9.62E-06 |
| cg13921352 |  | 3 |  | 68981890 |  | FAM19A4 |  | 0.693 |  | 0.239 |  | 0.455 |  | 1.59E-04 |  | 0.418 |  | 3.76E-33 |  | -2.758 |  | 4.88E-22 |
| cg03186486 |  | 3 |  | 68980947 |  | FAM19A4 |  | 0.543 |  | 0.088 |  | 0.454 |  | 2.05E-03 |  | 0.373 |  | 2.10E-18 |  | -2.758 |  | 4.88E-22 |
| cg23458558 |  | 8 |  | 85096868 |  | RALYL |  | 0.632 |  | 0.178 |  | 0.454 |  | 2.92E-04 |  | 0.434 |  | 6.13E-34 |  | -3.144 |  | 4.93E-29 |
| cg03625010 |  | 12 |  | 24715484 |  | SOX5 |  | 0.467 |  | 0.013 |  | 0.454 |  | 3.09E-03 |  | 0.357 |  | 1.06E-13 |  | -1.403 |  | 1.26E-17 |
| cg02761480 |  | 16 |  | 77822771 |  | VAT1L |  | 0.643 |  | 0.189 |  | 0.454 |  | 7.36E-04 |  | 0.378 |  | 4.55E-28 |  | -2.094 |  | 7.22E-62 |
| cg15405439 |  | 3 |  | 96533530 |  | EPHA6 |  | 0.693 |  | 0.240 |  | 0.453 |  | 9.29E-05 |  | 0.360 |  | 5.55E-28 |  | -4.332 |  | 2.42E-78 |
| cg22802813 |  | 12 |  | 95942761 |  | USP44 |  | 0.718 |  | 0.265 |  | 0.453 |  | 6.45E-05 |  | 0.448 |  | 3.38E-47 |  | -1.156 |  | 1.08E-10 |
| cg04190807 |  | 16 |  | 58497230 |  | NDRG4 |  | 0.554 |  | 0.101 |  | 0.453 |  | 2.33E-04 |  | 0.400 |  | 4.53E-23 |  | -1.187 |  | 2.35E-22 |
| cg15356923 |  | 3 |  | 68981011 |  | FAM19A4 |  | 0.567 |  | 0.114 |  | 0.453 |  | 2.39E-04 |  | 0.382 |  | 4.34E-23 |  | -2.758 |  | 4.88E-22 |
| cg04003850 |  | 3 |  | 192126165 |  | FGF12 |  | 0.610 |  | 0.157 |  | 0.453 |  | 2.27E-04 |  | 0.383 |  | 2.08E-35 |  | -1.036 |  | 1.80E-10 |
| cg11648594 |  | 1 |  | 1566860 |  | MMP23A |  | 0.594 |  | 0.142 |  | 0.452 |  | 1.56E-03 |  | 0.386 |  | 2.32E-20 |  | -1.281 |  | 1.90E-05 |
| cg17509967 |  | 19 |  | 13617094 |  | CACNA1A |  | 0.684 |  | 0.233 |  | 0.452 |  | 7.17E-05 |  | 0.416 |  | 5.77E-38 |  | -1.407 |  | 1.61E-21 |
| cg03112087 |  | 4 |  | 142053720 |  | RNF150 |  | 0.696 |  | 0.244 |  | 0.452 |  | 4.65E-05 |  | 0.436 |  | 6.92E-48 |  | -3.394 |  | 6.25E-98 |
| cg05349062 |  | 2 |  | 74741853 |  | TLX2 |  | 0.595 |  | 0.143 |  | 0.452 |  | 3.15E-04 |  | 0.364 |  | 2.16E-20 |  | -1.289 |  | 9.33E-11 |
| cg20225749 |  | 2 |  | 946127 |  | SNTG2 |  | 0.505 |  | 0.054 |  | 0.451 |  | 1.23E-03 |  | 0.385 |  | 1.62E-17 |  | -2.364 |  | 1.88E-28 |
| cg03629943 |  | 10 |  | 101089149 |  | CNNM1 |  | 0.730 |  | 0.279 |  | 0.451 |  | 4.90E-05 |  | 0.449 |  | 2.56E-58 |  | -1.045 |  | 2.68E-06 |
| cg16783279 |  | 10 |  | 125425625 |  | GPR26 |  | 0.781 |  | 0.331 |  | 0.450 |  | 5.10E-05 |  | 0.480 |  | 3.34E-58 |  | -2.255 |  | 3.08E-13 |
| cg27662483 |  | 4 |  | 156588683 |  | GUCY1A3 |  | 0.566 |  | 0.116 |  | 0.450 |  | 3.75E-04 |  | 0.400 |  | 1.57E-29 |  | -1.375 |  | 2.55E-16 |
| cg17498296 |  | 10 |  | 124907540 |  | HMX2 |  | 0.495 |  | 0.045 |  | 0.450 |  | 6.58E-04 |  | 0.375 |  | 1.31E-15 |  | -1.577 |  | 1.54E-06 |
| cg00866976 |  | 16 |  | 56224782 |  | GNAO1 |  | 0.530 |  | 0.081 |  | 0.450 |  | 1.76E-03 |  | 0.395 |  | 2.77E-21 |  | -3.588 |  | 3.11E-121 |
| cg14243481 |  | 11 |  | 7272867 |  | SYT9 |  | 0.690 |  | 0.240 |  | 0.450 |  | 7.15E-05 |  | 0.416 |  | 5.55E-32 |  | -2.647 |  | 2.49E-31 |
| cg20265733 |  | 20 |  | 61051032 |  | GATA5 |  | 0.655 |  | 0.206 |  | 0.449 |  | 1.40E-03 |  | 0.550 |  | 2.53E-29 |  | -2.304 |  | 5.49E-13 |
| cg16409955 |  | 13 |  | 38444227 |  | TRPC4 |  | 0.651 |  | 0.205 |  | 0.446 |  | 1.23E-03 |  | 0.392 |  | 6.57E-36 |  | -1.043 |  | 8.13E-13 |
| cg15031661 |  | 1 |  | 240256603 |  | FMN2 |  | 0.682 |  | 0.236 |  | 0.446 |  | 1.33E-03 |  | 0.413 |  | 4.58E-19 |  | -3.449 |  | 4.77E-48 |
| cg26433102 |  | 6 |  | 391441 |  | IRF4 |  | 0.646 |  | 0.201 |  | 0.446 |  | 1.25E-04 |  | 0.381 |  | 3.08E-50 |  | -2.419 |  | 2.40E-49 |
| cg15146859 |  | 18 |  | 74961737 |  | GALR1 |  | 0.601 |  | 0.155 |  | 0.446 |  | 7.48E-04 |  | 0.447 |  | 7.93E-21 |  | -3.710 |  | 1.01E-57 |
| cg26110710 |  | 13 |  | 88323607 |  | SLITRK5 |  | 0.497 |  | 0.051 |  | 0.446 |  | 1.22E-02 |  | 0.377 |  | 2.77E-11 |  | -1.305 |  | 8.07E-06 |
| cg02582387 |  | 1 |  | 20879813 |  | FAM43B |  | 0.681 |  | 0.235 |  | 0.445 |  | 4.20E-05 |  | 0.400 |  | 4.36E-38 |  | -1.562 |  | 1.80E-13 |
| cg11723848 |  | 4 |  | 96470286 |  | UNC5C |  | 0.630 |  | 0.186 |  | 0.445 |  | 5.47E-05 |  | 0.379 |  | 2.29E-51 |  | -2.323 |  | 6.43E-92 |
| cg07173275 |  | 1 |  | 57890636 |  | DAB1 |  | 0.655 |  | 0.210 |  | 0.445 |  | 1.64E-03 |  | 0.466 |  | 1.33E-21 |  | -1.050 |  | 9.62E-06 |
| cg19651132 |  | 12 |  | 5018805 |  | KCNA1 |  | 0.585 |  | 0.141 |  | 0.444 |  | 7.63E-04 |  | 0.456 |  | 5.75E-42 |  | -3.896 |  | 4.28E-50 |
| cg06193383 |  | 16 |  | 10275767 |  | GRIN2A |  | 0.539 |  | 0.095 |  | 0.444 |  | 4.35E-04 |  | 0.352 |  | 3.96E-21 |  | -2.691 |  | 6.46E-32 |
| cg18505401 |  | 7 |  | 19812570 |  | TMEM196 |  | 0.640 |  | 0.196 |  | 0.444 |  | 2.86E-04 |  | 0.404 |  | 1.36E-25 |  | -2.732 |  | 4.84E-24 |
| cg20702559 |  | 8 |  | 58907821 |  | FAM110B |  | 0.521 |  | 0.078 |  | 0.444 |  | 1.86E-04 |  | 0.489 |  | 1.51E-29 |  | -1.448 |  | 2.40E-23 |
| cg22647407 |  | 19 |  | 56904965 |  | ZNF582 |  | 0.695 |  | 0.251 |  | 0.443 |  | 4.79E-05 |  | 0.391 |  | 1.23E-27 |  | -1.345 |  | 5.51E-20 |
| cg15720995 |  | 12 |  | 29936972 |  | TMTC1 |  | 0.709 |  | 0.266 |  | 0.443 |  | 1.07E-04 |  | 0.375 |  | 2.38E-34 |  | -1.822 |  | 3.70E-37 |
| cg23967169 |  | 3 |  | 68981503 |  | FAM19A4 |  | 0.747 |  | 0.304 |  | 0.443 |  | 2.11E-04 |  | 0.351 |  | 2.58E-29 |  | -2.758 |  | 4.88E-22 |
| cg03403065 |  | 20 |  | 41818358 |  | PTPRT |  | 0.704 |  | 0.261 |  | 0.443 |  | 2.96E-04 |  | 0.507 |  | 3.61E-52 |  | -1.704 |  | 3.19E-16 |
| cg18278265 |  | 20 |  | 61051438 |  | GATA5 |  | 0.540 |  | 0.098 |  | 0.442 |  | 1.04E-04 |  | 0.410 |  | 7.42E-28 |  | -2.304 |  | 5.49E-13 |
| cg13845982 |  | 20 |  | 61051029 |  | GATA5 |  | 0.682 |  | 0.240 |  | 0.441 |  | 1.90E-03 |  | 0.536 |  | 6.56E-29 |  | -2.304 |  | 5.49E-13 |
| cg14400886 |  | 4 |  | 134070441 |  | PCDH10 |  | 0.663 |  | 0.222 |  | 0.441 |  | 1.27E-04 |  | 0.356 |  | 4.22E-25 |  | -2.971 |  | 2.07E-39 |
| cg15637465 |  | 5 |  | 168728270 |  | SLIT3 |  | 0.647 |  | 0.206 |  | 0.441 |  | 4.19E-04 |  | 0.426 |  | 7.36E-31 |  | -2.537 |  | 2.57E-65 |
| cg25825488 |  | 10 |  | 15761876 |  | ITGA8 |  | 0.701 |  | 0.260 |  | 0.440 |  | 8.39E-05 |  | 0.387 |  | 2.79E-41 |  | -1.870 |  | 4.80E-60 |
| cg01718116 |  | 2 |  | 115919829 |  | DPP10 |  | 0.703 |  | 0.263 |  | 0.440 |  | 7.14E-05 |  | 0.437 |  | 7.18E-29 |  | -1.786 |  | 2.51E-08 |
| cg17567560 |  | 10 |  | 105036863 |  | INA |  | 0.473 |  | 0.034 |  | 0.439 |  | 9.16E-04 |  | 0.418 |  | 3.57E-24 |  | -2.726 |  | 9.36E-33 |
| cg15343119 |  | 18 |  | 74961785 |  | GALR1 |  | 0.701 |  | 0.262 |  | 0.439 |  | 1.40E-04 |  | 0.404 |  | 8.01E-28 |  | -3.710 |  | 1.01E-57 |
| cg20776829 |  | 4 |  | 90758797 |  | SNCA |  | 0.516 |  | 0.077 |  | 0.439 |  | 1.18E-03 |  | 0.394 |  | 1.36E-11 |  | -1.934 |  | 3.83E-28 |
| cg22295787 |  | 8 |  | 26723584 |  | ADRA1A |  | 0.611 |  | 0.172 |  | 0.439 |  | 1.25E-04 |  | 0.431 |  | 3.34E-34 |  | -2.833 |  | 2.07E-47 |
| cg05118638 |  | 4 |  | 126237371 |  | FAT4 |  | 0.673 |  | 0.234 |  | 0.439 |  | 1.38E-03 |  | 0.370 |  | 3.81E-23 |  | -1.136 |  | 2.29E-13 |
| cg17222500 |  | 12 |  | 85306741 |  | SLC6A15 |  | 0.598 |  | 0.159 |  | 0.439 |  | 6.85E-05 |  | 0.409 |  | 5.91E-40 |  | -1.371 |  | 1.55E-04 |
| cg14256587 |  | 2 |  | 946010 |  | SNTG2 |  | 0.719 |  | 0.280 |  | 0.439 |  | 1.29E-04 |  | 0.415 |  | 3.45E-22 |  | -2.364 |  | 1.88E-28 |
| cg02856953 |  | 1 |  | 154474530 |  | TDRD10 |  | 0.499 |  | 0.060 |  | 0.439 |  | 2.13E-03 |  | 0.357 |  | 6.30E-12 |  | -1.718 |  | 9.74E-42 |
| cg05075118 |  | 3 |  | 132756991 |  | TMEM108 |  | 0.781 |  | 0.343 |  | 0.438 |  | 9.00E-05 |  | 0.390 |  | 6.04E-28 |  | -1.931 |  | 2.61E-51 |
| cg20174636 |  | 2 |  | 220299900 |  | SPEG |  | 0.749 |  | 0.311 |  | 0.437 |  | 5.52E-05 |  | 0.394 |  | 1.29E-31 |  | -3.012 |  | 8.27E-66 |
| cg18174928 |  | 5 |  | 38557085 |  | LIFR |  | 0.511 |  | 0.075 |  | 0.437 |  | 3.58E-04 |  | 0.356 |  | 5.62E-25 |  | -3.382 |  | 6.98E-134 |
| cg07219542 |  | 1 |  | 110210770 |  | GSTM2 |  | 0.592 |  | 0.155 |  | 0.436 |  | 2.09E-05 |  | 0.406 |  | 9.74E-40 |  | -1.554 |  | 3.90E-44 |
| cg11097433 |  | 10 |  | 108924557 |  | SORCS1 |  | 0.686 |  | 0.250 |  | 0.436 |  | 9.18E-05 |  | 0.451 |  | 7.21E-43 |  | -3.835 |  | 2.70E-84 |
| cg24454144 |  | 20 |  | 37353126 |  | SLC32A1 |  | 0.777 |  | 0.341 |  | 0.436 |  | 4.93E-05 |  | 0.365 |  | 7.07E-39 |  | -1.547 |  | 2.14E-04 |
| cg19898128 |  | 4 |  | 166794971 |  | TLL1 |  | 0.518 |  | 0.083 |  | 0.436 |  | 4.10E-03 |  | 0.484 |  | 1.17E-28 |  | -2.406 |  | 3.96E-40 |
| cg12748607 |  | 2 |  | 40678691 |  | SLC8A1 |  | 0.592 |  | 0.158 |  | 0.435 |  | 1.75E-03 |  | 0.355 |  | 1.35E-35 |  | -1.676 |  | 1.70E-42 |
| cg06733470 |  | 20 |  | 37435204 |  | PPP1R16B |  | 0.508 |  | 0.073 |  | 0.435 |  | 1.64E-04 |  | 0.374 |  | 2.80E-34 |  | -1.764 |  | 2.82E-42 |
| cg14733048 |  | 8 |  | 109095782 |  | RSPO2 |  | 0.634 |  | 0.199 |  | 0.435 |  | 2.34E-04 |  | 0.363 |  | 2.46E-35 |  | -4.155 |  | 1.48E-92 |
| cg24190603 |  | 6 |  | 84418433 |  | SNAP91 |  | 0.885 |  | 0.450 |  | 0.434 |  | 3.25E-05 |  | 0.488 |  | 1.01E-71 |  | -3.160 |  | 4.28E-42 |
| cg12740757 |  | 2 |  | 30144623 |  | ALK |  | 0.484 |  | 0.050 |  | 0.434 |  | 4.34E-03 |  | 0.373 |  | 2.26E-19 |  | -2.020 |  | 1.27E-11 |
| cg02539855 |  | 8 |  | 144241434 |  | LY6H |  | 0.477 |  | 0.044 |  | 0.433 |  | 1.31E-03 |  | 0.457 |  | 1.56E-28 |  | -2.123 |  | 9.32E-27 |
| cg20285745 |  | 12 |  | 117798627 |  | NOS1 |  | 0.818 |  | 0.385 |  | 0.433 |  | 8.46E-06 |  | 0.386 |  | 6.35E-40 |  | -3.377 |  | 5.07E-46 |
| cg11355135 |  | 2 |  | 108602860 |  | SLC5A7 |  | 0.797 |  | 0.364 |  | 0.433 |  | 5.94E-05 |  | 0.355 |  | 1.04E-27 |  | -4.278 |  | 1.41E-70 |
| cg00333226 |  | 7 |  | 49813763 |  | VWC2 |  | 0.626 |  | 0.193 |  | 0.433 |  | 3.18E-04 |  | 0.374 |  | 4.46E-31 |  | -2.201 |  | 5.96E-27 |
| cg20271517 |  | 4 |  | 156129862 |  | NPY2R |  | 0.564 |  | 0.132 |  | 0.432 |  | 7.56E-04 |  | 0.408 |  | 2.16E-29 |  | -4.485 |  | 8.01E-72 |
| cg19502867 |  | 8 |  | 58907088 |  | FAM110B |  | 0.566 |  | 0.134 |  | 0.432 |  | 7.59E-04 |  | 0.426 |  | 1.77E-25 |  | -1.448 |  | 2.40E-23 |
| cg20375342 |  | 3 |  | 68981895 |  | FAM19A4 |  | 0.646 |  | 0.214 |  | 0.432 |  | 7.25E-04 |  | 0.401 |  | 9.64E-31 |  | -2.758 |  | 4.88E-22 |
| cg24928391 |  | 8 |  | 55370336 |  | SOX17 |  | 0.654 |  | 0.222 |  | 0.432 |  | 1.90E-03 |  | 0.495 |  | 1.78E-38 |  | -1.065 |  | 4.37E-15 |
| cg08392199 |  | 5 |  | 38556223 |  | LIFR |  | 0.667 |  | 0.236 |  | 0.431 |  | 2.86E-04 |  | 0.360 |  | 3.81E-37 |  | -3.382 |  | 6.98E-134 |
| cg09463233 |  | 3 |  | 33260366 |  | SUSD5 |  | 0.542 |  | 0.111 |  | 0.431 |  | 7.96E-05 |  | 0.374 |  | 2.33E-33 |  | -1.819 |  | 8.86E-23 |
| cg05168033 |  | 2 |  | 56150942 |  | EFEMP1 |  | 0.557 |  | 0.126 |  | 0.431 |  | 4.84E-04 |  | 0.354 |  | 4.00E-27 |  | -1.459 |  | 5.03E-19 |
| cg06577205 |  | 5 |  | 15500714 |  | FBXL7 |  | 0.765 |  | 0.335 |  | 0.430 |  | 6.38E-05 |  | 0.417 |  | 1.21E-30 |  | -1.414 |  | 5.12E-29 |
| cg22266749 |  | 4 |  | 110223959 |  | COL25A1 |  | 0.735 |  | 0.305 |  | 0.430 |  | 1.06E-04 |  | 0.466 |  | 2.13E-40 |  | -1.087 |  | 1.63E-05 |
| cg06008912 |  | 2 |  | 193059645 |  | TMEFF2 |  | 0.723 |  | 0.293 |  | 0.430 |  | 6.40E-05 |  | 0.442 |  | 2.94E-44 |  | -4.134 |  | 3.79E-97 |
| cg17457560 |  | 8 |  | 32405851 |  | NRG1 |  | 0.684 |  | 0.254 |  | 0.430 |  | 1.01E-04 |  | 0.380 |  | 1.73E-26 |  | -1.447 |  | 5.33E-16 |
| cg17543296 |  | 1 |  | 20880378 |  | FAM43B |  | 0.663 |  | 0.233 |  | 0.430 |  | 5.89E-04 |  | 0.387 |  | 3.91E-31 |  | -1.562 |  | 1.80E-13 |
| cg06630204 |  | 12 |  | 29937343 |  | TMTC1 |  | 0.518 |  | 0.088 |  | 0.429 |  | 3.71E-04 |  | 0.415 |  | 1.92E-30 |  | -1.822 |  | 3.70E-37 |
| cg18976437 |  | 1 |  | 9712576 |  | PIK3CD |  | 0.520 |  | 0.091 |  | 0.429 |  | 1.26E-04 |  | 0.405 |  | 4.37E-32 |  | -1.046 |  | 4.42E-16 |
| cg11389172 |  | 10 |  | 50818299 |  | SLC18A3 |  | 0.625 |  | 0.196 |  | 0.429 |  | 6.73E-05 |  | 0.374 |  | 4.14E-37 |  | -2.734 |  | 1.25E-18 |
| cg17334018 |  | 4 |  | 96470293 |  | UNC5C |  | 0.536 |  | 0.108 |  | 0.428 |  | 1.02E-04 |  | 0.356 |  | 3.20E-47 |  | -2.323 |  | 6.43E-92 |
| cg12271981 |  | 13 |  | 88326752 |  | SLITRK5 |  | 0.668 |  | 0.240 |  | 0.428 |  | 1.13E-04 |  | 0.351 |  | 4.47E-36 |  | -1.305 |  | 8.07E-06 |
| cg05385513 |  | 2 |  | 56150478 |  | EFEMP1 |  | 0.742 |  | 0.314 |  | 0.428 |  | 6.75E-04 |  | 0.363 |  | 1.17E-30 |  | -1.459 |  | 5.03E-19 |
| cg24946597 |  | 8 |  | 31497464 |  | NRG1 |  | 0.778 |  | 0.350 |  | 0.428 |  | 1.19E-04 |  | 0.469 |  | 2.71E-43 |  | -1.447 |  | 5.33E-16 |
| cg08906194 |  | 9 |  | 34577972 |  | CNTFR |  | 0.716 |  | 0.288 |  | 0.428 |  | 7.66E-04 |  | 0.393 |  | 3.85E-26 |  | -3.741 |  | 2.66E-54 |
| cg12307306 |  | 3 |  | 134369987 |  | KY |  | 0.561 |  | 0.133 |  | 0.428 |  | 2.11E-03 |  | 0.397 |  | 2.46E-24 |  | -2.631 |  | 4.72E-53 |
| cg19901801 |  | 1 |  | 114697032 |  | SYT6 |  | 0.742 |  | 0.314 |  | 0.428 |  | 1.09E-04 |  | 0.371 |  | 1.23E-33 |  | -1.841 |  | 2.36E-13 |
| cg01764020 |  | 13 |  | 38444087 |  | TRPC4 |  | 0.687 |  | 0.259 |  | 0.427 |  | 1.42E-04 |  | 0.372 |  | 1.62E-36 |  | -1.043 |  | 8.13E-13 |
| cg11595545 |  | 1 |  | 111217497 |  | KCNA3 |  | 0.803 |  | 0.376 |  | 0.427 |  | 1.89E-04 |  | 0.394 |  | 5.16E-49 |  | -2.192 |  | 3.66E-35 |
| cg05207495 |  | 14 |  | 63512710 |  | KCNH5 |  | 0.678 |  | 0.251 |  | 0.427 |  | 2.09E-04 |  | 0.358 |  | 1.52E-28 |  | -1.306 |  | 1.52E-03 |
| cg10158080 |  | 12 |  | 24715864 |  | SOX5 |  | 0.446 |  | 0.020 |  | 0.427 |  | 1.03E-02 |  | 0.380 |  | 1.07E-11 |  | -1.403 |  | 1.26E-17 |
| cg06288251 |  | 3 |  | 132757087 |  | TMEM108 |  | 0.705 |  | 0.279 |  | 0.426 |  | 5.25E-05 |  | 0.384 |  | 2.60E-36 |  | -1.931 |  | 2.61E-51 |
| cg00590851 |  | 13 |  | 27335089 |  | GPR12 |  | 0.559 |  | 0.132 |  | 0.426 |  | 7.38E-05 |  | 0.354 |  | 8.02E-17 |  | -2.780 |  | 1.20E-21 |
| cg23650423 |  | 11 |  | 101454676 |  | TRPC6 |  | 0.673 |  | 0.247 |  | 0.426 |  | 1.48E-04 |  | 0.438 |  | 9.58E-43 |  | -1.238 |  | 2.67E-20 |
| cg03159124 |  | 18 |  | 7118077 |  | LAMA1 |  | 0.571 |  | 0.146 |  | 0.425 |  | 1.16E-04 |  | 0.394 |  | 1.28E-29 |  | -2.622 |  | 1.55E-68 |
| cg02459604 |  | 19 |  | 37407284 |  | ZNF568 |  | 0.622 |  | 0.197 |  | 0.425 |  | 1.17E-03 |  | 0.433 |  | 1.31E-32 |  | -1.326 |  | 1.57E-19 |
| cg24109980 |  | 10 |  | 50818707 |  | SLC18A3 |  | 0.753 |  | 0.328 |  | 0.425 |  | 5.51E-05 |  | 0.388 |  | 7.10E-36 |  | -2.734 |  | 1.25E-18 |
| cg19478298 |  | 7 |  | 103630093 |  | RELN |  | 0.732 |  | 0.308 |  | 0.424 |  | 6.39E-05 |  | 0.384 |  | 3.88E-32 |  | -2.738 |  | 1.53E-45 |
| cg07121547 |  | 4 |  | 156129169 |  | NPY2R |  | 0.463 |  | 0.039 |  | 0.424 |  | 8.44E-04 |  | 0.376 |  | 9.17E-22 |  | -4.485 |  | 8.01E-72 |
| cg10569606 |  | 12 |  | 5019004 |  | KCNA1 |  | 0.588 |  | 0.165 |  | 0.424 |  | 2.20E-04 |  | 0.408 |  | 1.22E-35 |  | -3.896 |  | 4.28E-50 |
| cg20634738 |  | 11 |  | 22214683 |  | ANO5 |  | 0.513 |  | 0.089 |  | 0.424 |  | 3.07E-03 |  | 0.404 |  | 6.43E-13 |  | -3.178 |  | 6.05E-57 |
| cg15975865 |  | 2 |  | 127413831 |  | GYPC |  | 0.759 |  | 0.336 |  | 0.423 |  | 1.10E-04 |  | 0.392 |  | 1.28E-53 |  | -1.743 |  | 3.37E-45 |
| cg16933181 |  | 18 |  | 24765485 |  | CHST9 |  | 0.666 |  | 0.243 |  | 0.423 |  | 2.74E-03 |  | 0.359 |  | 4.55E-12 |  | -2.307 |  | 1.87E-10 |
| cg06728579 |  | 16 |  | 56224901 |  | GNAO1 |  | 0.471 |  | 0.048 |  | 0.422 |  | 3.81E-03 |  | 0.372 |  | 2.10E-10 |  | -3.588 |  | 3.11E-121 |
| cg20061155 |  | 8 |  | 109095388 |  | RSPO2 |  | 0.674 |  | 0.252 |  | 0.422 |  | 9.73E-05 |  | 0.438 |  | 1.45E-52 |  | -4.155 |  | 1.48E-92 |
| cg27258025 |  | 19 |  | 52900974 |  | ZNF528 |  | 0.510 |  | 0.088 |  | 0.422 |  | 4.07E-04 |  | 0.439 |  | 4.85E-21 |  | -1.042 |  | 1.52E-09 |
| cg12664464 |  | 20 |  | 61051021 |  | GATA5 |  | 0.659 |  | 0.238 |  | 0.421 |  | 1.84E-03 |  | 0.529 |  | 7.88E-30 |  | -2.304 |  | 5.49E-13 |
| cg18884037 |  | 6 |  | 62996214 |  | KHDRBS2 |  | 0.657 |  | 0.236 |  | 0.421 |  | 8.05E-04 |  | 0.522 |  | 9.58E-49 |  | -4.225 |  | 2.32E-91 |
| cg03167429 |  | 11 |  | 126870429 |  | KIRREL3 |  | 0.495 |  | 0.074 |  | 0.421 |  | 1.38E-03 |  | 0.362 |  | 4.55E-22 |  | -1.946 |  | 3.09E-27 |
| cg14633783 |  | 8 |  | 21645870 |  | GFRA2 |  | 0.468 |  | 0.047 |  | 0.421 |  | 2.17E-03 |  | 0.362 |  | 1.13E-14 |  | -3.646 |  | 5.10E-159 |
| cg25013011 |  | 12 |  | 130389010 |  | TMEM132D |  | 0.541 |  | 0.121 |  | 0.420 |  | 1.90E-04 |  | 0.469 |  | 6.53E-31 |  | -1.718 |  | 2.72E-14 |
| cg25757598 |  | 8 |  | 85097195 |  | RALYL |  | 0.752 |  | 0.333 |  | 0.420 |  | 7.28E-05 |  | 0.455 |  | 1.11E-49 |  | -3.144 |  | 4.93E-29 |
| cg20140110 |  | 12 |  | 29936845 |  | TMTC1 |  | 0.750 |  | 0.331 |  | 0.420 |  | 7.34E-05 |  | 0.358 |  | 3.20E-37 |  | -1.822 |  | 3.70E-37 |
| cg04897742 |  | 1 |  | 16085148 |  | FBLIM1 |  | 0.437 |  | 0.018 |  | 0.419 |  | 1.57E-03 |  | 0.381 |  | 8.03E-37 |  | -1.290 |  | 1.45E-80 |
| cg20482521 |  | 10 |  | 106400667 |  | SORCS3 |  | 0.583 |  | 0.164 |  | 0.419 |  | 6.25E-04 |  | 0.385 |  | 4.14E-30 |  | -2.764 |  | 6.34E-24 |
| cg00197495 |  | 10 |  | 93392836 |  | PPP1R3C |  | 0.481 |  | 0.062 |  | 0.419 |  | 6.18E-03 |  | 0.390 |  | 3.00E-16 |  | -2.387 |  | 9.67E-50 |
| cg01663016 |  | 5 |  | 168727752 |  | SLIT3 |  | 0.739 |  | 0.321 |  | 0.419 |  | 7.73E-05 |  | 0.378 |  | 6.67E-48 |  | -2.537 |  | 2.57E-65 |
| cg26963356 |  | 6 |  | 55443833 |  | HMGCLL1 |  | 0.689 |  | 0.270 |  | 0.418 |  | 1.11E-03 |  | 0.360 |  | 1.69E-19 |  | -2.917 |  | 2.73E-79 |
| cg21217024 |  | 11 |  | 105481406 |  | GRIA4 |  | 0.590 |  | 0.172 |  | 0.417 |  | 9.36E-04 |  | 0.434 |  | 3.86E-33 |  | -2.716 |  | 1.42E-52 |
| cg02774439 |  | 4 |  | 174451251 |  | HAND2 |  | 0.575 |  | 0.158 |  | 0.417 |  | 1.12E-03 |  | 0.408 |  | 3.49E-26 |  | -4.035 |  | 3.30E-99 |
| cg11539780 |  | 19 |  | 57019016 |  | ZNF471 |  | 0.691 |  | 0.274 |  | 0.417 |  | 2.13E-04 |  | 0.422 |  | 1.68E-28 |  | -1.718 |  | 1.59E-22 |
| cg17239236 |  | 7 |  | 71802184 |  | CALN1 |  | 0.913 |  | 0.496 |  | 0.417 |  | 4.41E-05 |  | 0.402 |  | 1.23E-55 |  | -1.975 |  | 6.14E-16 |
| cg07921384 |  | 2 |  | 220299740 |  | SPEG |  | 0.631 |  | 0.215 |  | 0.417 |  | 3.46E-04 |  | 0.472 |  | 1.07E-39 |  | -3.012 |  | 8.27E-66 |
| cg12973941 |  | 4 |  | 13545639 |  | NKX3-2 |  | 0.758 |  | 0.342 |  | 0.416 |  | 1.64E-04 |  | 0.378 |  | 7.23E-48 |  | -1.918 |  | 1.44E-32 |
| cg23912454 |  | 4 |  | 156588387 |  | GUCY1A3 |  | 0.552 |  | 0.136 |  | 0.416 |  | 4.02E-04 |  | 0.419 |  | 4.13E-32 |  | -1.375 |  | 2.55E-16 |
| cg04075191 |  | 2 |  | 115919785 |  | DPP10 |  | 0.723 |  | 0.307 |  | 0.416 |  | 7.20E-05 |  | 0.407 |  | 2.05E-25 |  | -1.786 |  | 2.51E-08 |
| cg14777772 |  | 4 |  | 55098130 |  | PDGFRA |  | 0.442 |  | 0.026 |  | 0.416 |  | 9.69E-04 |  | 0.433 |  | 5.49E-30 |  | -1.185 |  | 1.63E-19 |
| cg12221475 |  | 6 |  | 1390622 |  | FOXF2 |  | 0.683 |  | 0.267 |  | 0.416 |  | 3.86E-03 |  | 0.405 |  | 4.77E-19 |  | -2.197 |  | 1.79E-66 |
| cg18277764 |  | 3 |  | 239188 |  | CHL1 |  | 0.678 |  | 0.263 |  | 0.415 |  | 3.90E-04 |  | 0.354 |  | 3.39E-35 |  | -2.660 |  | 2.33E-61 |
| cg26143045 |  | 3 |  | 132757610 |  | TMEM108 |  | 0.485 |  | 0.070 |  | 0.415 |  | 7.17E-04 |  | 0.383 |  | 5.11E-19 |  | -1.931 |  | 2.61E-51 |
| cg11016563 |  | 11 |  | 101454626 |  | TRPC6 |  | 0.627 |  | 0.212 |  | 0.415 |  | 1.00E-04 |  | 0.410 |  | 1.18E-38 |  | -1.238 |  | 2.67E-20 |
| cg07204280 |  | 18 |  | 12254551 |  | CIDEA |  | 0.750 |  | 0.335 |  | 0.415 |  | 8.53E-04 |  | 0.355 |  | 2.92E-35 |  | -1.867 |  | 5.19E-05 |
| cg24032666 |  | 8 |  | 24814035 |  | NEFL |  | 0.789 |  | 0.375 |  | 0.415 |  | 3.31E-04 |  | 0.359 |  | 9.48E-23 |  | -2.627 |  | 9.90E-21 |
| cg14163665 |  | 2 |  | 30144579 |  | ALK |  | 0.460 |  | 0.045 |  | 0.414 |  | 6.15E-03 |  | 0.464 |  | 4.12E-20 |  | -2.020 |  | 1.27E-11 |
| cg04804539 |  | 2 |  | 95691255 |  | MAL |  | 0.566 |  | 0.152 |  | 0.414 |  | 2.53E-04 |  | 0.399 |  | 1.77E-44 |  | -3.665 |  | 1.33E-128 |
| cg09083627 |  | 13 |  | 88324597 |  | SLITRK5 |  | 0.588 |  | 0.175 |  | 0.413 |  | 7.14E-03 |  | 0.478 |  | 2.04E-27 |  | -1.305 |  | 8.07E-06 |
| cg05893614 |  | 3 |  | 68981956 |  | FAM19A4 |  | 0.628 |  | 0.215 |  | 0.413 |  | 2.14E-04 |  | 0.406 |  | 2.27E-32 |  | -2.758 |  | 4.88E-22 |
| cg09722742 |  | 4 |  | 168155695 |  | SPOCK3 |  | 0.475 |  | 0.062 |  | 0.413 |  | 1.20E-03 |  | 0.355 |  | 1.55E-13 |  | -3.843 |  | 6.16E-46 |
| cg14370314 |  | 14 |  | 23834891 |  | EFS |  | 0.640 |  | 0.228 |  | 0.412 |  | 1.39E-04 |  | 0.437 |  | 6.55E-44 |  | -1.192 |  | 4.46E-19 |
| cg23771603 |  | 10 |  | 26223310 |  | MYO3A |  | 0.712 |  | 0.300 |  | 0.412 |  | 8.60E-05 |  | 0.376 |  | 6.62E-28 |  | -1.196 |  | 3.78E-05 |
| cg00329270 |  | 3 |  | 134369919 |  | KY |  | 0.557 |  | 0.146 |  | 0.412 |  | 5.85E-04 |  | 0.440 |  | 4.64E-36 |  | -2.631 |  | 4.72E-53 |
| cg09134593 |  | 11 |  | 130297528 |  | ADAMTS8 |  | 0.590 |  | 0.179 |  | 0.412 |  | 1.99E-04 |  | 0.382 |  | 3.90E-21 |  | -2.126 |  | 7.30E-38 |
| cg01423964 |  | 1 |  | 111217575 |  | KCNA3 |  | 0.575 |  | 0.164 |  | 0.411 |  | 7.43E-05 |  | 0.355 |  | 3.10E-46 |  | -2.192 |  | 3.66E-35 |
| cg15927720 |  | 5 |  | 168728149 |  | SLIT3 |  | 0.683 |  | 0.272 |  | 0.411 |  | 1.16E-04 |  | 0.474 |  | 3.45E-40 |  | -2.537 |  | 2.57E-65 |
| cg16368442 |  | 16 |  | 10276799 |  | GRIN2A |  | 0.442 |  | 0.032 |  | 0.410 |  | 1.13E-04 |  | 0.474 |  | 3.12E-48 |  | -2.691 |  | 6.46E-32 |
| cg18930994 |  | 4 |  | 156129424 |  | NPY2R |  | 0.647 |  | 0.237 |  | 0.409 |  | 2.08E-04 |  | 0.432 |  | 1.34E-30 |  | -4.485 |  | 8.01E-72 |
| cg22748799 |  | 19 |  | 58570790 |  | ZNF135 |  | 0.762 |  | 0.353 |  | 0.409 |  | 6.61E-05 |  | 0.405 |  | 1.79E-43 |  | -1.643 |  | 3.78E-30 |
| cg03091337 |  | 19 |  | 52901124 |  | ZNF528 |  | 0.557 |  | 0.148 |  | 0.409 |  | 4.88E-04 |  | 0.402 |  | 3.70E-22 |  | -1.042 |  | 1.52E-09 |
| cg14604568 |  | 7 |  | 32110523 |  | PDE1C |  | 0.546 |  | 0.137 |  | 0.409 |  | 7.78E-04 |  | 0.393 |  | 7.18E-20 |  | -1.555 |  | 8.21E-34 |
| cg10514097 |  | 16 |  | 86544787 |  | FOXF1 |  | 0.758 |  | 0.350 |  | 0.409 |  | 4.43E-05 |  | 0.379 |  | 5.98E-35 |  | -1.462 |  | 2.83E-36 |
| cg03336086 |  | 5 |  | 79331047 |  | THBS4 |  | 0.604 |  | 0.196 |  | 0.409 |  | 1.97E-03 |  | 0.387 |  | 3.58E-19 |  | -2.351 |  | 2.25E-20 |
| cg23649435 |  | 4 |  | 184826421 |  | STOX2 |  | 0.583 |  | 0.175 |  | 0.408 |  | 1.63E-03 |  | 0.390 |  | 2.47E-18 |  | -2.513 |  | 3.34E-75 |
| cg02745211 |  | 3 |  | 132757106 |  | TMEM108 |  | 0.753 |  | 0.345 |  | 0.408 |  | 8.00E-05 |  | 0.361 |  | 1.29E-37 |  | -1.931 |  | 2.61E-51 |
| cg11124364 |  | 7 |  | 19812850 |  | TMEM196 |  | 0.706 |  | 0.300 |  | 0.406 |  | 2.82E-05 |  | 0.377 |  | 1.38E-33 |  | -2.732 |  | 4.84E-24 |
| cg17617930 |  | 7 |  | 31091825 |  | ADCYAP1R1 |  | 0.462 |  | 0.056 |  | 0.406 |  | 1.23E-03 |  | 0.406 |  | 7.37E-21 |  | -4.346 |  | 6.97E-103 |
| cg10358533 |  | 19 |  | 34114209 |  | CHST8 |  | 0.706 |  | 0.300 |  | 0.406 |  | 4.57E-05 |  | 0.393 |  | 6.76E-35 |  | -1.580 |  | 4.92E-08 |
| cg17849956 |  | 19 |  | 57862480 |  | ZNF304 |  | 0.442 |  | 0.037 |  | 0.406 |  | 6.55E-04 |  | 0.358 |  | 2.28E-20 |  | -1.412 |  | 1.49E-19 |
| cg25539045 |  | 10 |  | 129535186 |  | FOXI2 |  | 0.756 |  | 0.351 |  | 0.406 |  | 7.40E-05 |  | 0.405 |  | 1.17E-35 |  | -1.803 |  | 3.01E-16 |
| cg16390060 |  | 20 |  | 21494084 |  | NKX2-2 |  | 0.700 |  | 0.294 |  | 0.405 |  | 6.69E-05 |  | 0.352 |  | 1.11E-28 |  | -1.556 |  | 2.30E-06 |
| cg19952303 |  | 12 |  | 85306323 |  | SLC6A15 |  | 0.533 |  | 0.128 |  | 0.405 |  | 1.54E-04 |  | 0.396 |  | 4.88E-41 |  | -1.371 |  | 1.55E-04 |
| cg18596362 |  | 18 |  | 70210955 |  | CBLN2 |  | 0.666 |  | 0.262 |  | 0.404 |  | 1.24E-04 |  | 0.433 |  | 9.40E-69 |  | -2.950 |  | 6.25E-75 |
| cg09552213 |  | 16 |  | 87636782 |  | JPH3 |  | 0.515 |  | 0.112 |  | 0.404 |  | 6.74E-04 |  | 0.368 |  | 4.54E-24 |  | -1.877 |  | 2.23E-20 |
| cg21068911 |  | 11 |  | 107462430 |  | ELMOD1 |  | 0.423 |  | 0.019 |  | 0.404 |  | 4.70E-04 |  | 0.364 |  | 6.45E-16 |  | -1.406 |  | 6.52E-10 |
| cg03753331 |  | 2 |  | 115919006 |  | DPP10 |  | 0.709 |  | 0.305 |  | 0.403 |  | 5.18E-05 |  | 0.472 |  | 3.59E-28 |  | -1.786 |  | 2.51E-08 |
| cg27486637 |  | 4 |  | 176987174 |  | WDR17 |  | 0.451 |  | 0.048 |  | 0.403 |  | 2.90E-03 |  | 0.492 |  | 2.56E-33 |  | -2.723 |  | 4.08E-37 |
| cg16987305 |  | 1 |  | 65991596 |  | LEPR |  | 0.797 |  | 0.394 |  | 0.403 |  | 6.28E-05 |  | 0.427 |  | 1.60E-28 |  | -1.466 |  | 6.17E-31 |
| cg25022687 |  | 10 |  | 118032987 |  | GFRA1 |  | 0.479 |  | 0.076 |  | 0.403 |  | 1.27E-03 |  | 0.354 |  | 2.59E-27 |  | -3.175 |  | 2.49E-85 |
| cg14233354 |  | 10 |  | 99790761 |  | CRTAC1 |  | 0.617 |  | 0.215 |  | 0.403 |  | 2.14E-05 |  | 0.470 |  | 1.33E-43 |  | -2.051 |  | 1.49E-17 |
| cg07073391 |  | 10 |  | 15761869 |  | ITGA8 |  | 0.580 |  | 0.177 |  | 0.403 |  | 1.73E-04 |  | 0.384 |  | 1.16E-35 |  | -1.870 |  | 4.80E-60 |
| cg03966785 |  | 4 |  | 55097476 |  | PDGFRA |  | 0.561 |  | 0.159 |  | 0.403 |  | 9.27E-04 |  | 0.373 |  | 6.64E-21 |  | -1.185 |  | 1.63E-19 |
| cg10453425 |  | 16 |  | 87636594 |  | JPH3 |  | 0.508 |  | 0.105 |  | 0.402 |  | 5.73E-04 |  | 0.369 |  | 1.44E-25 |  | -1.877 |  | 2.23E-20 |
| cg12426234 |  | 21 |  | 22370347 |  | NCAM2 |  | 0.625 |  | 0.223 |  | 0.402 |  | 6.64E-05 |  | 0.372 |  | 1.47E-33 |  | -1.359 |  | 4.53E-12 |
| cg11842610 |  | 10 |  | 118033762 |  | GFRA1 |  | 0.536 |  | 0.134 |  | 0.402 |  | 1.37E-03 |  | 0.373 |  | 8.52E-25 |  | -3.175 |  | 2.49E-85 |
| cg16138150 |  | 5 |  | 37839684 |  | GDNF |  | 0.587 |  | 0.185 |  | 0.402 |  | 1.45E-04 |  | 0.385 |  | 3.68E-31 |  | -2.297 |  | 8.35E-67 |
| cg20318608 |  | 1 |  | 179712591 |  | FAM163A |  | 0.581 |  | 0.180 |  | 0.401 |  | 5.24E-03 |  | 0.412 |  | 8.57E-20 |  | -2.126 |  | 3.17E-44 |
| cg06367693 |  | 4 |  | 184827491 |  | STOX2 |  | 0.568 |  | 0.168 |  | 0.401 |  | 2.42E-03 |  | 0.404 |  | 3.24E-26 |  | -2.513 |  | 3.34E-75 |
| cg08829841 |  | 10 |  | 129535348 |  | FOXI2 |  | 0.849 |  | 0.448 |  | 0.401 |  | 5.01E-05 |  | 0.398 |  | 1.59E-50 |  | -1.803 |  | 3.01E-16 |
| cg24287438 |  | 18 |  | 904878 |  | ADCYAP1 |  | 0.503 |  | 0.103 |  | 0.400 |  | 4.54E-03 |  | 0.380 |  | 1.08E-19 |  | -1.058 |  | 6.82E-06 |
| cg26715952 |  | 6 |  | 62996119 |  | KHDRBS2 |  | 0.645 |  | 0.245 |  | 0.400 |  | 7.17E-05 |  | 0.448 |  | 4.33E-62 |  | -4.225 |  | 2.32E-91 |
| cg18789663 |  | 1 |  | 242688591 |  | PLD5 |  | 0.601 |  | 0.201 |  | 0.400 |  | 3.53E-04 |  | 0.490 |  | 4.56E-34 |  | -3.256 |  | 1.14E-39 |
| cg25924217 |  | 17 |  | 78451563 |  | NPTX1 |  | 0.554 |  | 0.155 |  | 0.400 |  | 1.38E-03 |  | 0.416 |  | 1.16E-25 |  | -5.150 |  | 5.21E-150 |
| cg24424545 |  | 10 |  | 101089592 |  | CNNM1 |  | 0.625 |  | 0.226 |  | 0.399 |  | 4.00E-04 |  | 0.410 |  | 4.62E-51 |  | -1.045 |  | 2.68E-06 |
| cg04617755 |  | 2 |  | 115919366 |  | DPP10 |  | 0.523 |  | 0.125 |  | 0.398 |  | 8.08E-04 |  | 0.366 |  | 1.31E-21 |  | -1.786 |  | 2.51E-08 |
| cg26708235 |  | 13 |  | 25946397 |  | ATP8A2 |  | 0.607 |  | 0.210 |  | 0.398 |  | 1.14E-03 |  | 0.365 |  | 4.24E-24 |  | -1.867 |  | 1.51E-44 |
| cg02168857 |  | 6 |  | 94129611 |  | EPHA7 |  | 0.594 |  | 0.197 |  | 0.397 |  | 6.21E-03 |  | 0.378 |  | 7.16E-16 |  | -3.522 |  | 4.74E-63 |
| cg25201881 |  | 1 |  | 114695527 |  | SYT6 |  | 0.615 |  | 0.218 |  | 0.397 |  | 4.08E-04 |  | 0.361 |  | 1.84E-24 |  | -1.841 |  | 2.36E-13 |
| cg09426834 |  | 2 |  | 946648 |  | SNTG2 |  | 0.597 |  | 0.199 |  | 0.397 |  | 5.71E-04 |  | 0.456 |  | 7.20E-38 |  | -2.364 |  | 1.88E-28 |
| cg15060366 |  | 20 |  | 17206529 |  | PCSK2 |  | 0.607 |  | 0.210 |  | 0.397 |  | 1.06E-03 |  | 0.364 |  | 3.72E-16 |  | -4.954 |  | 3.62E-91 |
| cg26104297 |  | 10 |  | 15762076 |  | ITGA8 |  | 0.583 |  | 0.186 |  | 0.397 |  | 3.59E-04 |  | 0.385 |  | 4.47E-34 |  | -1.870 |  | 4.80E-60 |
| cg02174225 |  | 11 |  | 133938941 |  | JAM3 |  | 0.462 |  | 0.065 |  | 0.397 |  | 2.34E-04 |  | 0.360 |  | 2.98E-36 |  | -1.577 |  | 3.46E-50 |
| cg07013734 |  | 11 |  | 101454683 |  | TRPC6 |  | 0.559 |  | 0.162 |  | 0.397 |  | 3.96E-04 |  | 0.436 |  | 1.98E-34 |  | -1.238 |  | 2.67E-20 |
| cg26578621 |  | 11 |  | 110583377 |  | ARHGAP20 |  | 0.515 |  | 0.119 |  | 0.397 |  | 2.59E-04 |  | 0.385 |  | 8.07E-28 |  | -2.604 |  | 8.71E-68 |
| cg18239753 |  | 6 |  | 62995963 |  | KHDRBS2 |  | 0.719 |  | 0.323 |  | 0.396 |  | 1.04E-04 |  | 0.447 |  | 2.52E-58 |  | -4.225 |  | 2.32E-91 |
| cg27247697 |  | 2 |  | 87018054 |  | CD8A |  | 0.708 |  | 0.313 |  | 0.395 |  | 5.02E-05 |  | 0.358 |  | 1.42E-25 |  | -1.091 |  | 1.35E-09 |
| cg18315963 |  | 4 |  | 30723455 |  | PCDH7 |  | 0.708 |  | 0.313 |  | 0.395 |  | 2.06E-04 |  | 0.371 |  | 6.29E-35 |  | -1.556 |  | 3.36E-22 |
| cg00547077 |  | 19 |  | 58951756 |  | ZNF132 |  | 0.586 |  | 0.191 |  | 0.395 |  | 1.05E-03 |  | 0.374 |  | 3.91E-36 |  | -1.161 |  | 1.21E-40 |
| cg21782409 |  | 4 |  | 96470887 |  | UNC5C |  | 0.564 |  | 0.169 |  | 0.394 |  | 9.06E-04 |  | 0.483 |  | 5.75E-39 |  | -2.323 |  | 6.43E-92 |
| cg22603247 |  | 10 |  | 124907553 |  | HMX2 |  | 0.418 |  | 0.025 |  | 0.393 |  | 7.20E-04 |  | 0.371 |  | 2.51E-14 |  | -1.577 |  | 1.54E-06 |
| cg14070647 |  | 8 |  | 109095264 |  | RSPO2 |  | 0.550 |  | 0.157 |  | 0.393 |  | 9.94E-04 |  | 0.381 |  | 2.87E-41 |  | -4.155 |  | 1.48E-92 |
| cg20486569 |  | 22 |  | 19512228 |  | CLDN5 |  | 0.643 |  | 0.250 |  | 0.392 |  | 8.67E-05 |  | 0.353 |  | 1.91E-22 |  | -1.805 |  | 1.23E-30 |
| cg07039180 |  | 7 |  | 49814435 |  | VWC2 |  | 0.504 |  | 0.112 |  | 0.392 |  | 1.55E-02 |  | 0.411 |  | 1.31E-20 |  | -2.201 |  | 5.96E-27 |
| cg18443378 |  | 4 |  | 176986950 |  | WDR17 |  | 0.447 |  | 0.056 |  | 0.392 |  | 3.30E-03 |  | 0.456 |  | 1.96E-23 |  | -2.723 |  | 4.08E-37 |
| cg24621437 |  | 10 |  | 108924560 |  | SORCS1 |  | 0.672 |  | 0.281 |  | 0.391 |  | 6.07E-05 |  | 0.413 |  | 3.89E-41 |  | -3.835 |  | 2.70E-84 |
| cg01815529 |  | 20 |  | 17206946 |  | PCSK2 |  | 0.668 |  | 0.278 |  | 0.391 |  | 3.89E-04 |  | 0.357 |  | 1.37E-20 |  | -4.954 |  | 3.62E-91 |
| cg14997226 |  | 2 |  | 100938813 |  | LONRF2 |  | 0.408 |  | 0.017 |  | 0.391 |  | 1.44E-05 |  | 0.390 |  | 1.00E-54 |  | -3.768 |  | 7.89E-60 |
| cg08095852 |  | 4 |  | 176987020 |  | WDR17 |  | 0.477 |  | 0.086 |  | 0.391 |  | 3.21E-03 |  | 0.443 |  | 6.03E-32 |  | -2.723 |  | 4.08E-37 |
| cg00058329 |  | 12 |  | 133195094 |  | P2RX2 |  | 0.635 |  | 0.245 |  | 0.390 |  | 4.48E-03 |  | 0.431 |  | 9.21E-21 |  | -3.894 |  | 8.91E-88 |
| cg01802258 |  | 14 |  | 92790097 |  | SLC24A4 |  | 0.693 |  | 0.303 |  | 0.390 |  | 1.44E-04 |  | 0.381 |  | 5.45E-33 |  | -1.352 |  | 3.17E-21 |
| cg16899351 |  | 1 |  | 202679089 |  | SYT2 |  | 0.458 |  | 0.069 |  | 0.390 |  | 2.40E-03 |  | 0.365 |  | 2.23E-15 |  | -1.015 |  | 5.43E-09 |
| cg23572908 |  | 7 |  | 158937969 |  | VIPR2 |  | 0.899 |  | 0.510 |  | 0.390 |  | 2.57E-05 |  | 0.485 |  | 1.35E-49 |  | -2.010 |  | 4.28E-32 |
| cg04534765 |  | 18 |  | 74962369 |  | GALR1 |  | 0.752 |  | 0.362 |  | 0.389 |  | 8.06E-05 |  | 0.450 |  | 3.77E-57 |  | -3.710 |  | 1.01E-57 |
| cg24685755 |  | 19 |  | 53758031 |  | ZNF677 |  | 0.563 |  | 0.174 |  | 0.389 |  | 1.59E-04 |  | 0.403 |  | 4.84E-38 |  | -1.720 |  | 7.22E-37 |
| cg23971170 |  | 1 |  | 165326269 |  | LMX1A |  | 0.654 |  | 0.266 |  | 0.388 |  | 6.42E-03 |  | 0.517 |  | 1.99E-26 |  | -1.975 |  | 1.67E-09 |
| cg26721264 |  | 18 |  | 74961727 |  | GALR1 |  | 0.514 |  | 0.126 |  | 0.388 |  | 4.99E-03 |  | 0.380 |  | 3.33E-18 |  | -3.710 |  | 1.01E-57 |
| cg07176264 |  | 2 |  | 120281999 |  | SCTR |  | 0.735 |  | 0.346 |  | 0.388 |  | 8.07E-05 |  | 0.464 |  | 1.95E-38 |  | -1.869 |  | 3.17E-21 |
| cg18319029 |  | 18 |  | 904920 |  | ADCYAP1 |  | 0.679 |  | 0.291 |  | 0.388 |  | 1.43E-04 |  | 0.394 |  | 1.72E-29 |  | -1.058 |  | 6.82E-06 |
| cg10583000 |  | 2 |  | 98963134 |  | CNGA3 |  | 0.499 |  | 0.112 |  | 0.388 |  | 5.89E-04 |  | 0.394 |  | 6.90E-32 |  | -2.976 |  | 1.63E-30 |
| cg20450979 |  | 4 |  | 176923542 |  | GPM6A |  | 0.439 |  | 0.051 |  | 0.388 |  | 5.37E-05 |  | 0.362 |  | 9.20E-34 |  | -4.531 |  | 2.55E-79 |
| cg05239311 |  | 15 |  | 78913147 |  | CHRNA3 |  | 0.678 |  | 0.292 |  | 0.387 |  | 2.21E-03 |  | 0.370 |  | 4.01E-14 |  | -2.057 |  | 1.73E-27 |
| cg23113963 |  | 16 |  | 23313293 |  | SCNN1B |  | 0.495 |  | 0.109 |  | 0.387 |  | 2.13E-03 |  | 0.434 |  | 5.49E-22 |  | -3.950 |  | 1.67E-83 |
| cg16896079 |  | 12 |  | 41582362 |  | PDZRN4 |  | 0.708 |  | 0.321 |  | 0.387 |  | 1.19E-04 |  | 0.364 |  | 3.12E-52 |  | -4.306 |  | 2.76E-105 |
| cg15202738 |  | 19 |  | 53636108 |  | ZNF415 |  | 0.541 |  | 0.154 |  | 0.387 |  | 9.77E-03 |  | 0.423 |  | 5.07E-23 |  | -1.399 |  | 2.89E-15 |
| cg13724788 |  | 19 |  | 19322778 |  | NCAN |  | 0.629 |  | 0.242 |  | 0.387 |  | 1.42E-03 |  | 0.352 |  | 7.74E-23 |  | -1.140 |  | 5.28E-04 |
| cg14388488 |  | 20 |  | 61051777 |  | GATA5 |  | 0.457 |  | 0.071 |  | 0.386 |  | 4.40E-03 |  | 0.381 |  | 1.81E-13 |  | -2.304 |  | 5.49E-13 |
| cg17909890 |  | 9 |  | 22447694 |  | DMRTA1 |  | 0.535 |  | 0.149 |  | 0.386 |  | 1.58E-03 |  | 0.382 |  | 2.83E-28 |  | -3.425 |  | 2.36E-68 |
| cg06482428 |  | 4 |  | 21950173 |  | KCNIP4 |  | 0.707 |  | 0.321 |  | 0.386 |  | 5.77E-05 |  | 0.364 |  | 1.25E-32 |  | -2.411 |  | 3.25E-90 |
| cg25724283 |  | 13 |  | 36705475 |  | DCLK1 |  | 0.674 |  | 0.288 |  | 0.386 |  | 5.80E-05 |  | 0.379 |  | 1.19E-56 |  | -2.886 |  | 5.36E-68 |
| cg25932164 |  | 20 |  | 37434950 |  | PPP1R16B |  | 0.404 |  | 0.019 |  | 0.386 |  | 1.37E-03 |  | 0.469 |  | 1.10E-24 |  | -1.764 |  | 2.82E-42 |
| cg03878133 |  | 2 |  | 164593411 |  | FIGN |  | 0.726 |  | 0.340 |  | 0.386 |  | 1.47E-04 |  | 0.392 |  | 2.10E-31 |  | -1.773 |  | 5.16E-29 |
| cg21517947 |  | 8 |  | 41167107 |  | SFRP1 |  | 0.769 |  | 0.384 |  | 0.384 |  | 7.47E-05 |  | 0.392 |  | 1.82E-44 |  | -4.202 |  | 3.13E-91 |
| cg18725867 |  | 5 |  | 37834958 |  | GDNF |  | 0.776 |  | 0.392 |  | 0.384 |  | 1.22E-04 |  | 0.410 |  | 7.75E-36 |  | -2.297 |  | 8.35E-67 |
| cg12950007 |  | 19 |  | 52900970 |  | ZNF528 |  | 0.543 |  | 0.159 |  | 0.384 |  | 8.88E-04 |  | 0.380 |  | 2.67E-20 |  | -1.042 |  | 1.52E-09 |
| cg06133145 |  | 14 |  | 94254351 |  | PRIMA1 |  | 0.731 |  | 0.347 |  | 0.384 |  | 2.77E-04 |  | 0.351 |  | 6.47E-25 |  | -4.746 |  | 2.54E-122 |
| cg26684946 |  | 22 |  | 43739575 |  | SCUBE1 |  | 0.496 |  | 0.113 |  | 0.383 |  | 8.24E-03 |  | 0.430 |  | 3.99E-21 |  | -1.937 |  | 2.10E-40 |
| cg18624900 |  | 10 |  | 91295643 |  | SLC16A12 |  | 0.598 |  | 0.216 |  | 0.383 |  | 2.39E-04 |  | 0.357 |  | 3.97E-24 |  | -2.811 |  | 6.10E-71 |
| cg00812833 |  | 1 |  | 242687412 |  | PLD5 |  | 0.668 |  | 0.286 |  | 0.382 |  | 8.89E-05 |  | 0.434 |  | 2.31E-37 |  | -3.256 |  | 1.14E-39 |
| cg14980983 |  | 20 |  | 61051039 |  | GATA5 |  | 0.652 |  | 0.269 |  | 0.382 |  | 2.70E-03 |  | 0.526 |  | 1.19E-29 |  | -2.304 |  | 5.49E-13 |
| cg14470895 |  | 5 |  | 115152431 |  | CDO1 |  | 0.587 |  | 0.205 |  | 0.382 |  | 7.49E-04 |  | 0.386 |  | 1.24E-39 |  | -1.956 |  | 2.66E-22 |
| cg02043159 |  | 4 |  | 134070235 |  | PCDH10 |  | 0.639 |  | 0.258 |  | 0.381 |  | 1.27E-04 |  | 0.404 |  | 5.79E-26 |  | -2.971 |  | 2.07E-39 |
| cg07285673 |  | 2 |  | 29337984 |  | CLIP4 |  | 0.601 |  | 0.221 |  | 0.380 |  | 1.84E-03 |  | 0.373 |  | 5.19E-40 |  | -2.068 |  | 2.57E-41 |
| cg14831174 |  | 20 |  | 61050885 |  | GATA5 |  | 0.593 |  | 0.213 |  | 0.380 |  | 1.48E-03 |  | 0.434 |  | 3.72E-26 |  | -2.304 |  | 5.49E-13 |
| cg04050867 |  | 8 |  | 109095572 |  | RSPO2 |  | 0.601 |  | 0.222 |  | 0.379 |  | 8.67E-05 |  | 0.391 |  | 1.75E-51 |  | -4.155 |  | 1.48E-92 |
| cg05152589 |  | 3 |  | 85008678 |  | CADM2 |  | 0.616 |  | 0.237 |  | 0.379 |  | 2.00E-04 |  | 0.452 |  | 4.20E-32 |  | -4.575 |  | 2.02E-88 |
| cg16340268 |  | 1 |  | 226925172 |  | ITPKB |  | 0.427 |  | 0.049 |  | 0.378 |  | 5.90E-03 |  | 0.363 |  | 1.35E-19 |  | -1.037 |  | 1.22E-39 |
| cg15950068 |  | 6 |  | 84418659 |  | SNAP91 |  | 0.710 |  | 0.331 |  | 0.378 |  | 7.10E-04 |  | 0.417 |  | 2.03E-50 |  | -3.160 |  | 4.28E-42 |
| cg02914422 |  | 7 |  | 32110145 |  | PDE1C |  | 0.615 |  | 0.238 |  | 0.377 |  | 1.30E-04 |  | 0.353 |  | 1.20E-32 |  | -1.555 |  | 8.21E-34 |
| cg16642284 |  | 10 |  | 129535378 |  | FOXI2 |  | 0.654 |  | 0.277 |  | 0.377 |  | 4.59E-03 |  | 0.533 |  | 1.67E-36 |  | -1.803 |  | 3.01E-16 |
| cg17509220 |  | 19 |  | 13617012 |  | CACNA1A |  | 0.623 |  | 0.247 |  | 0.376 |  | 8.65E-05 |  | 0.402 |  | 5.60E-33 |  | -1.407 |  | 1.61E-21 |
| cg04408595 |  | 3 |  | 71802608 |  | EIF4E3 |  | 0.428 |  | 0.052 |  | 0.376 |  | 6.86E-04 |  | 0.402 |  | 7.47E-24 |  | -1.885 |  | 8.61E-74 |
| cg23524195 |  | 10 |  | 118034031 |  | GFRA1 |  | 0.521 |  | 0.145 |  | 0.376 |  | 3.11E-03 |  | 0.372 |  | 1.38E-19 |  | -3.175 |  | 2.49E-85 |
| cg01458504 |  | 4 |  | 156589291 |  | GUCY1A3 |  | 0.480 |  | 0.104 |  | 0.376 |  | 2.65E-03 |  | 0.383 |  | 5.28E-26 |  | -1.375 |  | 2.55E-16 |
| cg21384402 |  | 10 |  | 105036701 |  | INA |  | 0.626 |  | 0.251 |  | 0.376 |  | 1.04E-03 |  | 0.387 |  | 3.89E-30 |  | -2.726 |  | 9.36E-33 |
| cg21859781 |  | 8 |  | 97173034 |  | GDF6 |  | 0.531 |  | 0.156 |  | 0.375 |  | 8.26E-05 |  | 0.450 |  | 7.95E-56 |  | -1.283 |  | 9.70E-08 |
| cg10351287 |  | 4 |  | 5053341 |  | STK32B |  | 0.675 |  | 0.300 |  | 0.375 |  | 1.00E-04 |  | 0.435 |  | 4.44E-58 |  | -1.568 |  | 1.10E-29 |
| cg10002178 |  | 14 |  | 94254726 |  | PRIMA1 |  | 0.452 |  | 0.078 |  | 0.375 |  | 6.47E-03 |  | 0.460 |  | 1.33E-17 |  | -4.746 |  | 2.54E-122 |
| cg16711792 |  | 21 |  | 32930054 |  | TIAM1 |  | 0.510 |  | 0.136 |  | 0.374 |  | 2.91E-04 |  | 0.394 |  | 3.71E-35 |  | -1.251 |  | 2.18E-14 |
| cg21319053 |  | 5 |  | 37840725 |  | GDNF |  | 0.627 |  | 0.254 |  | 0.373 |  | 9.16E-04 |  | 0.352 |  | 1.00E-27 |  | -2.297 |  | 8.35E-67 |
| cg05932408 |  | 20 |  | 48184639 |  | PTGIS |  | 0.516 |  | 0.143 |  | 0.373 |  | 7.06E-04 |  | 0.372 |  | 1.78E-26 |  | -1.526 |  | 1.02E-12 |
| cg03502002 |  | 18 |  | 74962133 |  | GALR1 |  | 0.803 |  | 0.430 |  | 0.373 |  | 4.71E-05 |  | 0.554 |  | 2.62E-54 |  | -3.710 |  | 1.01E-57 |
| cg02484469 |  | 20 |  | 61051036 |  | GATA5 |  | 0.685 |  | 0.313 |  | 0.373 |  | 4.35E-03 |  | 0.512 |  | 1.33E-29 |  | -2.304 |  | 5.49E-13 |
| cg04482794 |  | 1 |  | 226925181 |  | ITPKB |  | 0.395 |  | 0.022 |  | 0.373 |  | 4.14E-03 |  | 0.372 |  | 3.79E-22 |  | -1.037 |  | 1.22E-39 |
| cg01896761 |  | 3 |  | 13590444 |  | FBLN2 |  | 0.623 |  | 0.251 |  | 0.372 |  | 1.51E-03 |  | 0.418 |  | 1.06E-13 |  | -1.310 |  | 4.92E-12 |
| cg14664759 |  | 1 |  | 196577723 |  | KCNT2 |  | 0.568 |  | 0.196 |  | 0.372 |  | 5.17E-03 |  | 0.404 |  | 1.59E-29 |  | -1.756 |  | 1.64E-21 |
| cg27278953 |  | 14 |  | 77737661 |  | NGB |  | 0.693 |  | 0.321 |  | 0.371 |  | 3.06E-05 |  | 0.375 |  | 5.18E-25 |  | -5.045 |  | 9.66E-94 |
| cg23353893 |  | 6 |  | 152958133 |  | SYNE1 |  | 0.402 |  | 0.031 |  | 0.370 |  | 2.61E-03 |  | 0.369 |  | 4.24E-15 |  | -1.507 |  | 6.57E-25 |
| cg18932798 |  | 10 |  | 105037503 |  | INA |  | 0.677 |  | 0.307 |  | 0.370 |  | 1.81E-04 |  | 0.368 |  | 1.60E-32 |  | -2.726 |  | 9.36E-33 |
| cg06401021 |  | 6 |  | 55443868 |  | HMGCLL1 |  | 0.652 |  | 0.282 |  | 0.370 |  | 1.88E-03 |  | 0.434 |  | 9.18E-20 |  | -2.917 |  | 2.73E-79 |
| cg10576051 |  | 15 |  | 73661652 |  | HCN4 |  | 0.444 |  | 0.075 |  | 0.368 |  | 3.21E-03 |  | 0.364 |  | 4.39E-15 |  | -1.415 |  | 1.19E-07 |
| cg07657064 |  | 19 |  | 58609764 |  | ZSCAN18 |  | 0.510 |  | 0.142 |  | 0.368 |  | 5.41E-03 |  | 0.351 |  | 2.18E-16 |  | -1.394 |  | 5.72E-18 |
| cg08463758 |  | 20 |  | 39995649 |  | EMILIN3 |  | 0.726 |  | 0.358 |  | 0.368 |  | 9.78E-05 |  | 0.355 |  | 6.42E-37 |  | -2.583 |  | 6.25E-83 |
| cg09186006 |  | 10 |  | 91295299 |  | SLC16A12 |  | 0.569 |  | 0.202 |  | 0.367 |  | 3.52E-04 |  | 0.416 |  | 4.21E-25 |  | -2.811 |  | 6.10E-71 |
| cg00287829 |  | 16 |  | 10276375 |  | GRIN2A |  | 0.509 |  | 0.142 |  | 0.367 |  | 1.17E-03 |  | 0.362 |  | 1.13E-24 |  | -2.691 |  | 6.46E-32 |
| cg14531560 |  | 1 |  | 16085371 |  | FBLIM1 |  | 0.575 |  | 0.208 |  | 0.367 |  | 2.50E-04 |  | 0.450 |  | 6.64E-54 |  | -1.290 |  | 1.45E-80 |
| cg00795341 |  | 5 |  | 79330929 |  | THBS4 |  | 0.643 |  | 0.276 |  | 0.366 |  | 9.68E-04 |  | 0.475 |  | 4.50E-25 |  | -2.351 |  | 2.25E-20 |
| cg03659519 |  | 18 |  | 74961966 |  | GALR1 |  | 0.670 |  | 0.303 |  | 0.366 |  | 6.15E-05 |  | 0.395 |  | 1.42E-31 |  | -3.710 |  | 1.01E-57 |
| cg25784220 |  | 19 |  | 58609602 |  | ZSCAN18 |  | 0.594 |  | 0.228 |  | 0.366 |  | 4.56E-03 |  | 0.362 |  | 2.22E-16 |  | -1.394 |  | 5.72E-18 |
| cg24680586 |  | 10 |  | 105036727 |  | INA |  | 0.608 |  | 0.242 |  | 0.366 |  | 9.90E-04 |  | 0.387 |  | 1.48E-27 |  | -2.726 |  | 9.36E-33 |
| cg09528265 |  | 5 |  | 146258195 |  | PPP2R2B |  | 0.432 |  | 0.066 |  | 0.366 |  | 9.16E-04 |  | 0.383 |  | 7.30E-17 |  | -2.147 |  | 4.81E-42 |
| cg15186181 |  | 8 |  | 55370434 |  | SOX17 |  | 0.637 |  | 0.271 |  | 0.365 |  | 1.15E-03 |  | 0.375 |  | 3.56E-39 |  | -1.065 |  | 4.37E-15 |
| cg24291087 |  | 4 |  | 30722781 |  | PCDH7 |  | 0.560 |  | 0.195 |  | 0.365 |  | 1.53E-02 |  | 0.425 |  | 1.24E-20 |  | -1.556 |  | 3.36E-22 |
| cg02881570 |  | 14 |  | 77737495 |  | NGB |  | 0.720 |  | 0.355 |  | 0.365 |  | 5.27E-05 |  | 0.437 |  | 2.67E-33 |  | -5.045 |  | 9.66E-94 |
| cg24320612 |  | 20 |  | 61051317 |  | GATA5 |  | 0.557 |  | 0.192 |  | 0.365 |  | 1.60E-03 |  | 0.427 |  | 3.88E-26 |  | -2.304 |  | 5.49E-13 |
| cg05552220 |  | 8 |  | 97173466 |  | GDF6 |  | 0.502 |  | 0.138 |  | 0.364 |  | 4.80E-04 |  | 0.366 |  | 1.13E-31 |  | -1.283 |  | 9.70E-08 |
| cg11826826 |  | 5 |  | 146258237 |  | PPP2R2B |  | 0.469 |  | 0.106 |  | 0.364 |  | 3.78E-04 |  | 0.374 |  | 1.37E-19 |  | -2.147 |  | 4.81E-42 |
| cg06223466 |  | 7 |  | 4922708 |  | RADIL |  | 0.425 |  | 0.061 |  | 0.363 |  | 5.26E-03 |  | 0.421 |  | 3.23E-21 |  | -1.429 |  | 2.11E-12 |
| cg21438101 |  | 18 |  | 904885 |  | ADCYAP1 |  | 0.518 |  | 0.154 |  | 0.363 |  | 1.07E-03 |  | 0.395 |  | 4.41E-22 |  | -1.058 |  | 6.82E-06 |
| cg25645268 |  | 4 |  | 154710598 |  | SFRP2 |  | 0.453 |  | 0.090 |  | 0.362 |  | 1.27E-03 |  | 0.408 |  | 2.54E-27 |  | -1.139 |  | 6.63E-05 |
| cg03929977 |  | 12 |  | 5019017 |  | KCNA1 |  | 0.538 |  | 0.177 |  | 0.361 |  | 7.03E-04 |  | 0.378 |  | 1.90E-38 |  | -3.896 |  | 4.28E-50 |
| cg02057391 |  | 13 |  | 38443783 |  | TRPC4 |  | 0.767 |  | 0.407 |  | 0.360 |  | 2.87E-03 |  | 0.429 |  | 4.17E-41 |  | -1.043 |  | 8.13E-13 |
| cg14658804 |  | 5 |  | 168728213 |  | SLIT3 |  | 0.741 |  | 0.382 |  | 0.360 |  | 8.20E-05 |  | 0.398 |  | 2.99E-36 |  | -2.537 |  | 2.57E-65 |
| cg08969532 |  | 10 |  | 99790438 |  | CRTAC1 |  | 0.568 |  | 0.210 |  | 0.358 |  | 6.41E-04 |  | 0.360 |  | 7.20E-21 |  | -2.051 |  | 1.49E-17 |
| cg00060320 |  | 3 |  | 134369974 |  | KY |  | 0.435 |  | 0.078 |  | 0.358 |  | 5.99E-03 |  | 0.411 |  | 8.48E-22 |  | -2.631 |  | 4.72E-53 |
| cg26295057 |  | 5 |  | 37839829 |  | GDNF |  | 0.724 |  | 0.366 |  | 0.357 |  | 1.34E-04 |  | 0.439 |  | 9.09E-35 |  | -2.297 |  | 8.35E-67 |
| cg25149751 |  | 5 |  | 146258427 |  | PPP2R2B |  | 0.521 |  | 0.164 |  | 0.357 |  | 1.58E-03 |  | 0.370 |  | 4.49E-20 |  | -2.147 |  | 4.81E-42 |
| cg13168820 |  | 20 |  | 41818356 |  | PTPRT |  | 0.610 |  | 0.253 |  | 0.357 |  | 4.25E-04 |  | 0.391 |  | 9.91E-49 |  | -1.704 |  | 3.19E-16 |
| cg00615473 |  | 21 |  | 32930423 |  | TIAM1 |  | 0.424 |  | 0.067 |  | 0.357 |  | 1.91E-03 |  | 0.390 |  | 4.95E-22 |  | -1.251 |  | 2.18E-14 |
| cg09912350 |  | 4 |  | 156588326 |  | GUCY1A3 |  | 0.563 |  | 0.207 |  | 0.356 |  | 2.91E-04 |  | 0.393 |  | 4.69E-31 |  | -1.375 |  | 2.55E-16 |
| cg19727439 |  | 1 |  | 37500508 |  | GRIK3 |  | 0.565 |  | 0.209 |  | 0.356 |  | 2.29E-04 |  | 0.366 |  | 1.73E-32 |  | -3.755 |  | 1.43E-150 |
| cg21013866 |  | 14 |  | 23834985 |  | EFS |  | 0.558 |  | 0.203 |  | 0.355 |  | 1.97E-04 |  | 0.403 |  | 1.84E-31 |  | -1.192 |  | 4.46E-19 |
| cg10345326 |  | 11 |  | 24518281 |  | LUZP2 |  | 0.494 |  | 0.139 |  | 0.355 |  | 3.56E-03 |  | 0.363 |  | 6.10E-22 |  | -2.270 |  | 6.26E-33 |
| cg12328333 |  | 13 |  | 36705041 |  | DCLK1 |  | 0.581 |  | 0.227 |  | 0.354 |  | 2.23E-04 |  | 0.422 |  | 1.43E-35 |  | -2.886 |  | 5.36E-68 |
| cg18428688 |  | 19 |  | 58609744 |  | ZSCAN18 |  | 0.538 |  | 0.184 |  | 0.354 |  | 7.34E-03 |  | 0.408 |  | 9.77E-17 |  | -1.394 |  | 5.72E-18 |
| cg09968630 |  | 11 |  | 22214554 |  | ANO5 |  | 0.444 |  | 0.090 |  | 0.354 |  | 2.04E-02 |  | 0.399 |  | 6.50E-12 |  | -3.178 |  | 6.05E-57 |
| cg25280433 |  | 6 |  | 105584216 |  | BVES |  | 0.439 |  | 0.085 |  | 0.354 |  | 3.76E-03 |  | 0.371 |  | 1.02E-24 |  | -2.897 |  | 7.75E-98 |
| cg12903638 |  | 2 |  | 210636350 |  | UNC80 |  | 0.522 |  | 0.169 |  | 0.353 |  | 1.27E-02 |  | 0.362 |  | 6.90E-14 |  | -2.322 |  | 4.72E-22 |
| cg12300353 |  | 4 |  | 44450358 |  | KCTD8 |  | 0.616 |  | 0.263 |  | 0.353 |  | 2.39E-04 |  | 0.360 |  | 6.79E-29 |  | -2.591 |  | 7.03E-18 |
| cg10406295 |  | 8 |  | 41167113 |  | SFRP1 |  | 0.789 |  | 0.437 |  | 0.352 |  | 6.43E-05 |  | 0.377 |  | 1.69E-39 |  | -4.202 |  | 3.13E-91 |
| cg19811761 |  | 19 |  | 57019022 |  | ZNF471 |  | 0.741 |  | 0.389 |  | 0.352 |  | 2.28E-04 |  | 0.362 |  | 8.54E-26 |  | -1.718 |  | 1.59E-22 |
| cg08185661 |  | 11 |  | 7273498 |  | SYT9 |  | 0.574 |  | 0.224 |  | 0.350 |  | 1.35E-04 |  | 0.363 |  | 4.41E-31 |  | -2.647 |  | 2.49E-31 |

CHR, Chromosome; CA-N, CRC surrounding normal tissue; CA, primary CRC; FC, fold change; FDR, adjusted *P* value with Benjamini-Hochberg method.
