# Supplementary figures and images for: Genome-wide methylation profiling identified novel differentially hypermethylated biomarker MPPED2 in colorectal cancer
Source: Clin Epigenetics. 2019 Mar 7;11:41. doi: 10.1186/s13148-019-0628-y (PMC6407227; doi:10.1186/s13148-019-0628-y)

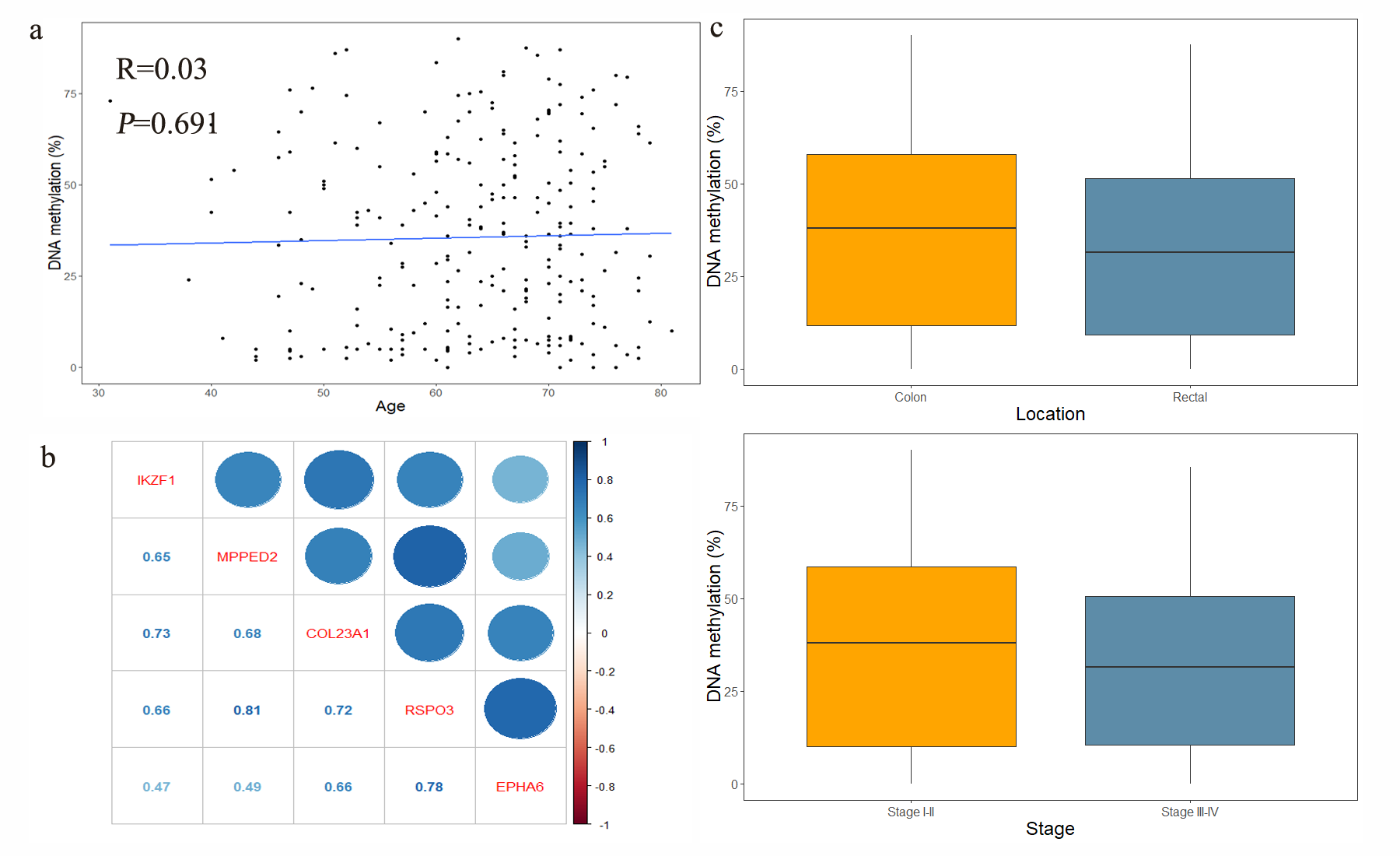

Supplement: Supplementary file 3 — Figure S1. a Pearson correlation analysis between age and methylation level of MPPED2. b Pearson correlation analysis between methylation levels of five selected genes c Comparisons of methylation level of MPPED2 in different stages and locations of CRC tissues in the validation phase. (TIF 628 kb) [file 13148_2019_628_MOESM3_ESM.tif]
