# Supplementary material for: Genome-wide methylation profiling identified novel differentially hypermethylated biomarker MPPED2 in colorectal cancer
Source: Clin Epigenetics. 2019 Mar 7;11:41. doi: 10.1186/s13148-019-0628-y (PMC6407227; doi:10.1186/s13148-019-0628-y)
Supplement: Supplementary file 4 — Table S3. Results of ROC curve analysis between CRC tissues and adjacent normal tissues. (DOCX 12 kb) [file 13148_2019_628_MOESM4_ESM.docx]

**Table S3.** Results of ROC curve analysis between CRC and paired normal tissues

| Gene symbol |  | Cutoff |  | Sensitivity |  | Specificity |  | AUC |
| --- | --- | --- | --- | --- | --- | --- | --- | --- |
| MPPED2 |  | 9.00 |  | 0.804 |  | 0.978 |  | 0.890 |
| RSPO3 |  | 8.00 |  | 0.761 |  | 0.978 |  | 0.875 |
| COL23A1 |  | 6.75 |  | 0.783 |  | 0.978 |  | 0.858 |
| IKZF1 |  | 5.50 |  | 0.783 |  | 0.978 |  | 0.840 |
| EPHA6 |  | 12.25 |  | 0.587 |  | 0.935 |  | 0.736 |
